# A transcriptomal analysis of bovine oviductal epithelial cells collected during the follicular phase versus the luteal phase of the estrous cycle

**DOI:** 10.1186/s12958-015-0077-1

**Published:** 2015-08-05

**Authors:** K. L. Cerny, E. Garrett, A.J. Walton, L.H. Anderson, P. J. Bridges

**Affiliations:** Department of Animal and Food Sciences, University of Kentucky, Lexington, 40546 KY USA

**Keywords:** Heifer, Oviduct epithelial cells, Ampulla, Isthmus, Estradiol, Progesterone

## Abstract

**Background:**

Reproductive success depends on a functional oviduct for gamete storage, maturation, fertilization, and early embryonic development. The ovarian-derived steroids estrogen and progesterone are key regulators of oviductal function. The objective of this study was to investigate luteal and follicular phase-specific oviductal epithelial cell function by using microarray-based transcriptional profiling, to increase our understanding of mRNAs regulating epithelial cell processes, and to identify novel genes and biochemical pathways that may be found to affect fertility in the future.

**Methods:**

Six normally cycling Angus heifers were assigned to either luteal phase (LP, *n* = 3) or follicular phase (FP, *n* = 3) treatment groups. Heifers in the LP group were killed between day 11 and 12 after estrus. Heifers in the FP group were treated with 25 mg PGF_2α_ (Lutalyse, Pfizer, NY) at 8 pm on day 6 after estrus and killed 36 h later. Transcriptional profiling by microarray and confirmation of selected mRNAs by real-time RT-PCR analyses was performed using total RNA from epithelial cells isolated from sections of the ampulla and isthmus collected from LP and FP treatment groups. Differentially expressed genes were subjected to gene ontology classification and bioinformatic pathway analyses.

**Results:**

Statistical one-way ANOVA using Benjamini-hochberg multiple testing correction for false discovery rate (FDR) and pairwise comparison of epithelial cells in the ampulla of FP versus LP groups revealed 972 and 597 transcripts up- and down-regulated, respectively (P < 0.05). Within epithelial cells of the isthmus in FP versus LP groups, 946 and 817 transcripts were up- and down-regulated, respectively (P < 0.05). Up-regulated genes from both ampulla and isthmus were found to be largely involved in cholesterol biosynthesis and cell cycle pathways, while down-regulated genes were found in numerous inflammatory response pathways.

**Conclusions:**

Microarray-based transcriptional profiling revealed phase of the cycle-dependent changes in the expression of mRNA within the epithelium of the oviducts’ ampulla and isthmus.

**Electronic supplementary material:**

The online version of this article (doi:10.1186/s12958-015-0077-1) contains supplementary material, which is available to authorized users.

## Background

Reproductive success depends on a functional oviduct for gamete storage and maturation, fertilization, and early embryonic development. Ovarian-derived steroids are well known regulators of oviductal function. Both estrogen and the progesterone receptors are abundant in the bovine oviducts’ mucosal epithelium [1–6], yet our understanding of how the steroidal environment affects the ability of the oviduct to function remains only partially understood. Maturation of gametes and breeding will occur in an estrogen dominant environment, fertilization and early cleavage after the steroidogenic shift and later stages of cleavage and formation of the morula occur within an oviduct exposed to increasing concentrations of circulating progesterone. Increasing our understanding of the steroidal control of oviductal function is critical to the design and implementation of interventions used to manage breeding and the establishment of a pregnancy.

Functionally, the oviduct is divided into two distinct segments; the upper ampulla situated immediately below the ovarian bursa and infundibulum, and the lower isthmus which spans the interval from the ampulla to the uterus. The epithelial mucosa within these two sections consists of ciliated and secretory simple columnar epithelial cells [7], the ratio of which is regulated by steroid hormones [8, 9]. Steroidal regulation of processes that facilitate sperm binding [10], sperm release [11], capacitation [12] and hyperactivation [13] are all established in the literature. Epithelial cells are also an active site of biosynthesis and secretion. A 3- to 5-fold increase in the rate of oviductal secretions can be expected around the time of estrus [14] and amino acids including glycine, glutamate, aspartate, alanine and lysine are all found in higher concentrations in oviductal fluid than in peripheral plasma [15]. Overall, steroid-regulated epithelial cell secretions can be considered an important mediator of the microenvironment that facilitates gamete and zygote health and early development.

While major efforts have been directed at investigating the ovary and uterus at the transcriptional level [16–18], less extensive investigation has been directed towards the oviduct. Suppressive subtractive hybridization was used in the detailed study of Bauersachs et al., [19], in which changes in gene expression within the oviductal epithelium were determined in heifers killed on the morning of estrus or 12 days thereafter; our report expanding on their analysis with the use of more current transcriptomal profiling technologies and the determination of spatial differences between the ampulla and the isthmus.

The objective of this descriptive study was therefore to determine global oviductal epithelial cell gene expression profiles during the follicular and luteal phases of the estrous cycle. Specifically, microarray-based transcriptional profiling was used to identify spatial and phase of the cycle-dependent changes in mRNA expression in epithelial cells isolated from the ampulla and isthmus, with the overall goals of increasing our understanding of epithelial processes and identifying novel genes that may be identified as key regulators of fertility in the future. Our results must be interpreted, however, with the knowledge that this analysis does not extract potential spatial (isthmus to ampulla and *visa versa*) signaling mechanisms that could affect oviductal epithelial cell gene expression profiles independent to phase of the estrous cycle. Given the size of the dataset generated by this analysis, our approach to this descriptive study is not to provide a detailed discussion of genes or processes affected by phase of the estrous cycle, but to summarize the results generated, provide our bioinformatic analyses (as Tables and Supplemental Tables) and make available our data for further analysis by others. The microarray raw data (*.cel files) collected with the GCOS software, plus the RMA-normalized and log2 transformed transcript data (Park Genomics Suite [20]), have been deposited into the Gene Expression Omnibus (National Center for Biotechnology Information, http://www.ncbi.nlm.nih.gov/geo) as accession number GSE63969.

## Methods

### Animals and tissue collection

Animal procedures involved in this study were approved by the University of Kentucky Animal Care and Use Committee. Six normally cycling Angus heifers were used and at least one spontaneous ovulation was observed in each animal prior to being included in the study. All animals were monitored for behavioral estrus (Day 0) and examined a minimum of every other day by trans-rectal ultrasonography throughout the study period, as described before [21, 22]. After ovulation was confirmed, heifers were assigned to either luteal phase (LP) or follicular phase (FP) treatment groups. Heifers in the LP group (*n* = 3) were killed between day 11 and 12 after estrus. The rationale for collection of LP oviducts on Day 11 or 12 post-estrus was to collect samples under a stable, high progestogenic environment, at a time when the oviduct is undergoing progesterone-dependent remodeling and repair. Heifers in the FP group (*n* = 3) were treated with 25 mg PGF_2α_ (Lutalyse, Pfizer, New York, NY) at 8 pm on day 6 after estrus and killed 36 h later. This is an established protocol in which the dominant follicle of the first follicular wave of the estrous cycle is induced to differentiate into a preovulatory follicle [21–24]. The diameter of the preovulatory follicle (FP group) and the corpus luteum (LP group) prior to retrieval of the tissues was 14.6 +/− 1.0 mm and 21.5 +/− 0.8 mm, respectively. Stage of the cycle was confirmed by visual appearance of the ovaries collected at slaughter.

Heifers were killed by stunning with a captive-bolt pistol and exsanguinated in the Department of Animal and Food Sciences’ USDA approved Meat Science Lab. Immediately after exsanguination, the oviduct ipsilateral to the corpus luteum (LP group) or preovulatory follicle (FP group) was dissected free from surrounding connective tissue. Epithelial cells were isolated from small sections of the ampulla and isthmus by using a well-established technique for the collection of bovine oviductal epithelial cells [25–27]. Briefly, sections of ampulla and isthmus were gently squeezed with fine forceps under a dissecting microscope to separate epithelial cells from residual stroma. Epithelial cells were briefly centrifuged to form a pellet and then snap-frozen in liquid N_2_ for later extraction of RNA. Small sections of the ampulla and isthmus were also fixed for 24 h in Bouin’s fixative (Sigma-Aldrich, St. Louis, MO) then processed, sectioned and stained by the University of Kentucky Imaging Facility. Tissues were processed through an ascending series of graded ethyl alcohols, xylene and paraffin then embedded in paraffin. Embedded tissues were sectioned on a microtome at 5 μm, floated on to SuperFrost Plus slides then heat-fixed for a minimum of 2 h at 48 °C on a slide warmer. Hematoxylin and Eosin staining was performed using a ThermoShandon GLX Slide Stainer with Hematoxylin (EKI hematoxylin solution Gills III) and Eosin Y.

### RNA Extraction and analysis

Epithelial cells were isolated from the ampulla and isthmus of the oviduct ispilateral to the ovary bearing the preovulatory follicle (FP group) or the corpus luteum (LP group). Total RNA was extracted from each sample of epithelial cells using TRIzol reagent (Invitrogen Corporation, Carlsbad, CA, USA) and purified with RNeasy columns (QIAGEN, Valencia, CA) according to the manufacturer’s instructions. RNA quality was analyzed by determining the RNA integrity number (RIN) using an Agilent 2100 Bioanalyzer (Agilent Technologies, Palo Alto, CA) at the University of Kentucky Microarray Core Facility. RNA integrity numbers were greater than 9.2 and 28S/18S rRNA absorbance ratios greater than 1.5 for all samples. RNA concentration was then determined via spectrophotometry using a NanoDrop 2000 (Thermo Fischer Scienctific-NanoDrop products, Wilmington, DE, USA). Spectrophotometry results revealed 260/280 absorbance ratios greater than 1.95 and 260/230 absorbance ratios greater than 1.5 for all samples.

### Microarray analysis

The Bovine gene 1.0 ST array (GeneChip; Affymetrix, Inc., Santa Clara, CA) was used. Microarray analysis was conducted according to the manufacturer’s instructions at the University of Kentucky Microarray Core Facility, as described before [28, 29]. Briefly, RNA (3 μg/sample) was reverse transcribed to cDNA using primers containing T7 RNA polymerase, so that the resulting cDNA contained the T7 sequence. In-vitro transcription was then used for the preparation and labeling of cRNA. The biotinylated cRNA were further fragmented and used as probes to hybridize the GeneChips in the GeneChip Hybridization Oven 640, using 1 chip per RNA sample. The raw expression intensity values generated by microarray hybridization were imported into Partek Genomics Suite 6.6 (Partek Inc., St. Louis, MO). Robust Multiarray Analysis algorithm, quantile normalization, and Median Polish were applied for GeneChip background correction, log base_2_ transformation, conversion of expression values and probeset summarization [30, 31]. Transcripts were annotated using NetAffx annotation database for the Bovine gene 1.0 ST array and last updated in June 2014.

After data were processed for background adjustment, normalization and log_2_ transformed, quality of data was assessed using light intensity expression values on a per chip and per gene basis. For statistical analysis, Partek Genomics Suite 6.6 (Partek Inc.) was used to complete an *F*-test on least-square means to determine significance of each transcript in each comparison. Benjamini-hochberg multiple testing correction for false discovery rate (FDR) was applied and significance set to FDR adjusted P-value < 0.05. A post-hoc pairwise comparison of FP compared to LP epithelial cells from the ampulla and isthmus was completed using Fisher’s Least Significant Difference (LSD) to determine which means differed [32, 33]. Only transcripts with a fold-change value of ≥ 1.5 were included in the results. The raw data (*.cel files), plus the RMA-normalized and log_2_ transformed transcript data (Park Genomics Suite [31], have been deposited into the Gene Expression Omnibus (National Center for Biotechnology Information [34]) as accession number GSE63969 (http://www.ncbi.nlm.nih.gov/geo/query/acc.cgi?acc=GSE63969).

### Real-time RT-PCR analysis

Real-time RT-PCR was performed using RNA samples from each tissue and phase of the estrous cycle to determine the expression of mRNA for neurotensin (NTS), binder of sperm 3 (BSP3), lactate dehydrogenase A (LDHA), cyclin D2 (CCND2), early growth response 1 (EGR1) and hydroxysteroid (17β) dehydrogenase 7 (HSD17B7) using an Eppendorf Mastercycler ep *realplex*^2^ system (Eppendorf, Hamburg, Germany) with iQ SYBR Green Supermix (Bio-RAD, Hercules, CA), as described before [29]. Additional validation of the Bovine gene 1.0 ST array has been reported by our laboratory [35]. The following oligonucleotide primer pairs (5’ to 3’) were used: NTS, F: GTG TGG AAA TGT GAC AGA GCA C and R: GGT AGG CTA GAC TTT GCG GT; BSP3, F: ATT CCT GTG GTG TTC CCT CG and R: GCT CAG AGC ATC ACC TTT GC; LDHA, F: CCA ACA TGG CAG CCT TTT CC and R: ACC GCT TTC CAC TGT TCC TT; CCND2, F: CCG ACA ACT CCA TCA AGC CT and R: TGA AGT AGT GGC GCA CAG AG; EGR1, F: AGA AAG TTT GCC AGG AGC GA and R: GGA GGG ACG GAG GAG TAT GT; HSD17B7, F: ACA GCT GAA GGA CTG CTG AC and R: CCA GAC AGT GCT TCT GTT CCA; and 18S, F: CGG GGA GGT AGT GAC GAA A and R: CCG CTC CCA AGA TCC AAC TA.

Briefly, cDNA was synthesized using the SuperScript III 1^st^ Strand Synthesis System (Invitrogen), with 0.5 μg of RNA used for each reverse transcription reaction. Real-time RT-PCR was performed with a total volume of 25 μL per reaction, with each reaction containing 5 μL of cDNA, 1 μL of a 10 μM stock of each primer (forward and reverse), 12.5 μL of 2× SYBR Green PCR Master Mix, and 5.5 μL of nuclease-free water. The typical dissociation curves of these cDNA, plus 18S as the housekeeping gene was confirmed. RT-PCR reactions were run in triplicate and gene expression was analyzed by the 2^ΔΔ^CT method [36].

### Gene ontology and pathway analysis

Differentially expressed transcripts were interrogated for their gene ontology classes using Partek Genomics Suite 6.6 (Partek Inc.). Partek derives biological processes, molecular functions and cellular components from geneontology.org and/or the affymetrix database. GO hierarchies leads to division of the gene list into significant classifications using Fischer’s exact test-right tailed. When the observed number of genes in a GO category is greater than expected, the GO category is enriched. Pathway analysis was completed by importing differentially expressed transcripts into QIAGEN’S Ingenuity Pathway Analysis (IPA, QIAGEN, Redwood City, CA, USA, www.qiagen.com/ingenuity). Ingenuity Pathway Analysis uses data from multiple databases to extrapolate significant pathways and Fischer’s exact test was used to determine significant pathways. Significance was set to P-value < 0.05.

## Results and discussion

### Real-time RT- PCR analysis of selected transcripts

The effect of stage of the estrous cycle and tissue on the expression of mRNA for NTS, BSP3, LDHA, CCND2, EGR1 and HSD17B7 was performed by real-time RT-PCR. A comparison of the results obtained by RT-PCR and microarray analysis is described in Table 1 as a validation of the microarray platform. Additional validation of this Bovine gene 1.0 ST array by real-time RT-PCR performed within our laboratory has been reported. Overall, RT-PCR revealed the same directional trends in gene expression as microarray analysis, with the magnitude of these changes typically lower after analysis using the microarray platform (ratio compression phenomena), as described by others [37]. For example, the expression of mRNA for NTS was found to increase by 32.4- and 150-fold within epithelial cells of the ampulla, and 21.6- and 88-fold within the epithelial cells of the isthmus, in the FP versus the LP groups by microarray analysis and real-time RT-PCR, respectively.

**Table 1:** Comparison of gene expression for selected mRNAs by microarray and real-time RT-PCR

Ampulla:	Microarray		Real-time RT-PCR					
Gene Symbol	FP vs. LP	FP vs. LP	FP Mean	SEM	LP Mean	SEM	Fold-Change	*P*-value
	Fold-Change	*P*-value						
NTS	32.44	<0.001	1.05	0.12	0.007	0.001	150	<0.001
BSP3	−1.09	0.794	ND					
LDHA	2.22	<0.001	1.13	0.20	0.43	0.10	2.63	0.002
CCND2	1.23	0.352	1.19	0.21	0.91	0.12	1.31	0.273
EGR1	−9.13	0.005	1.19	0.23	111.9	34.2	−94	<.001
HSD17B7	1.03	0.762	1.07	0.21	0.86	0.12	1.24	0.361
Isthmus:	Microarray		Real-time RT-PCR					
Gene Symbol	FP vs. LP	FP vs. LP	FP Mean	SEM	LP Mean	SEM	Fold-Change	*P*-value
	Fold-Change	*P*-value						
NTS	21.57	<0.001	1.51	0.47	0.017	0.008	88	<0.001
BSP3	−7.57561	<0.001	1.91	0.74	30.47	11.39	−16	0.013
LDHA	2.33775	<0.001	1.05	0.12	0.29	0.08	3.64	<0.001
CCND2	2.36116	0.003	1.09	0.18	0.80	0.17	1.38	0.238
EGR1	5.35334	0.019	1.07	0.13	108.4	35.2	−101	<0.001
HSD17B7	−1.13469	0.271	1.05	0.12	1.76	0.36	−1.67	0.251

For statistical analysis of the microarray dataset, an F-test on least-square means was used to determine significance of each transcript in each comparison. Benjamini-hochberg multiple testing correction for false discovery rate (FDR) was applied and significance set to FDR adjusted P-value < 0.05. A post-hoc pairwise comparison of FP compared to LP epithelial cells was completed using Fisher’s Least Significant Difference (LSD) to determine which means differed

NTS: neurotensin, BSP3: binder of sperm 3, LDHA: lactate dehydrogenase A, CCND2: cyclin D2, EGR1: early growth response 1, HSD17B7: hydroxysteroid (17β) dehydrogenase 7

For the analysis of gene expression by real-time RT-PCR, mean level of expression and SEM are indicated for FP and LP epithelial cells of the ampulla and isthmus, as well as fold change in relative expression (FP vs. LP). For statistical analysis of relative gene expression by real-time RT-PCR, P-values to determine the significance of each transcript were determined using the Student’s *t*-test

ND: Not detectable by real-time RT-PCR

### Microarray quality control and principal component analysis

**Fig. 1:**
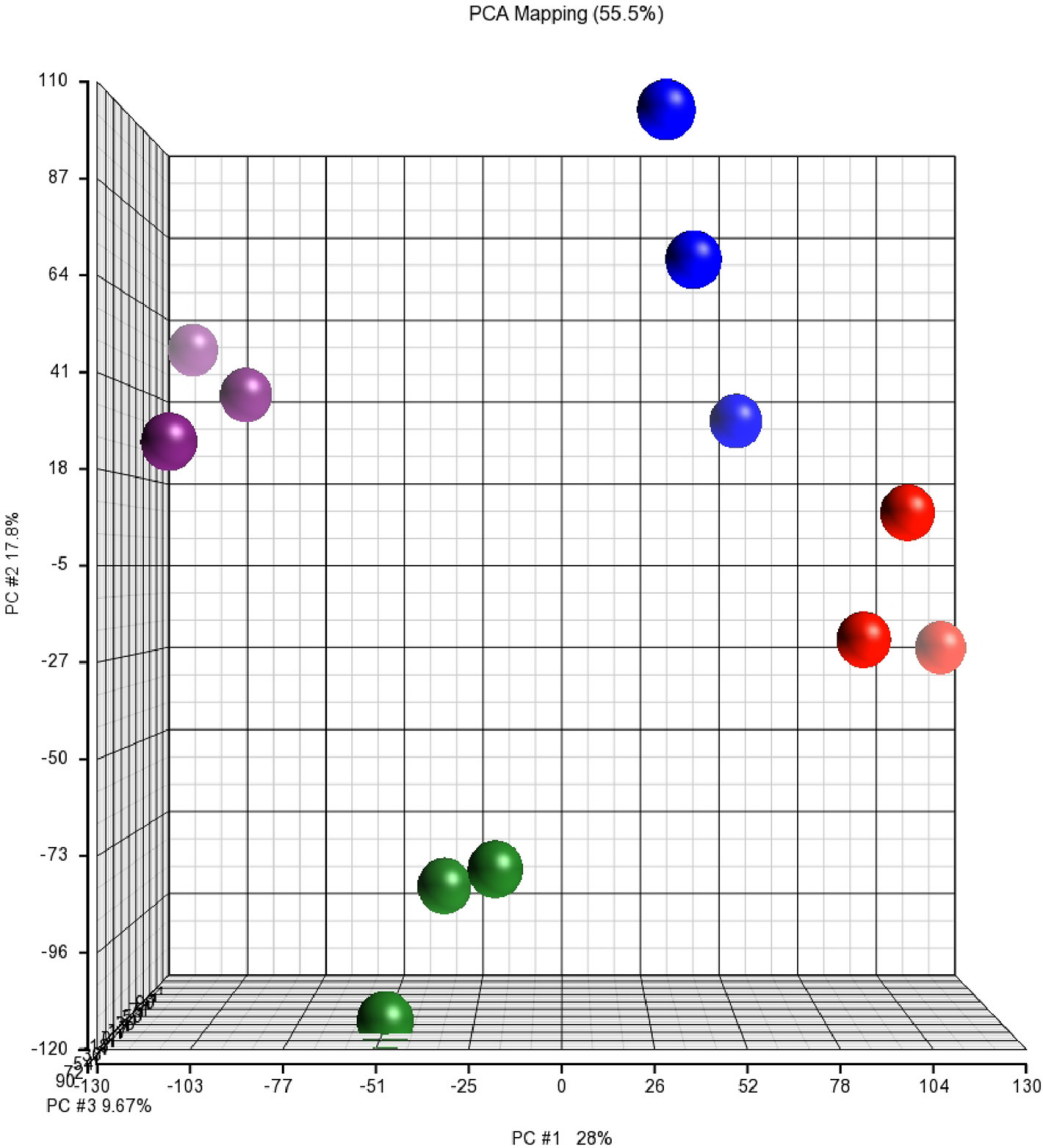
Principal component analysis of microarray transcriptome results of epithelial cells from the ampulla and isthmus taken from heifers killed in the follicular phase (FP) and luteal phase (LP) of the estrous cycle. Red: FP ampulla, Blue: FP isthmus, Green: LP ampulla, Purple: LP isthmus. Each Bovine 1.0 ST array includes 26,775 probesets

Box plots revealed mean intensity values were similar across all chips and overlapping histograms indicated the frequency of transcripts at specific intensity values for each chip were similar (Additional file 1: Figure S2 and Additional file 2: Figure S2). Quantification of signal intensity to noise revealed that spatial location and phase of the cycle, not error, accounted for the variation within the data set (mean F Ratio for attribute = 30.78, versus mean F value for error = 1.0). Consistent with this, principal component analysis (PCA), which allows for the visualization of patterns through the distribution of samples to highlight similarities and differences [31], revealed clear differences between LP and FP groups, as well as tissue-specific differences within the same phase of the estrous cycle (Fig. 1). Total variance (55.5 %) is the cumulative percent of variance accounted for in our datasets based upon eigenvector multivariate analysis. PC#1 (x-axis, 28 %) indicates that the largest proportion of variability is due to phase of the estrous cycle. PC#2 (y-axis, 17.8 %) indicates variability between the ampulla and isthmus. PC#3 (z-axis, 9.7 %) indicates the variability between phase of the estrous cycle and tissues.

### Pairwise comparisons

**Fig. 2:**
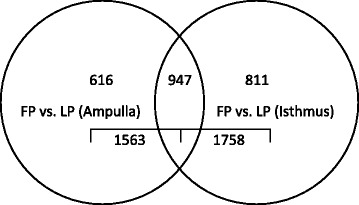
Venn diagram depicting the number of differentially expressed genes in follicular phase versus luteal phase epithelial cells from the ampulla and isthmus (FDR adjusted *P* < 0.05)

It is well known that function of the mammalian oviduct is influenced by estradiol and progesterone, with previous studies confirming the presence of mRNA and protein for estrogen receptor alpha (ERα) and beta (ERβ) as well as the nuclear and membrane progesterone receptors (PR, PGRMC1, PGRMC2) within this organ [6, 38]. After chip normalization, a statistical one-way ANOVA and pairwise comparison (LSD test) of gene expression in epithelial cells of the ampulla and isthmus in the FP and LP groups was performed to generate a list of 2374 differentially expressed genes (DEGs). Table 2 indicates the number of DEGs in each pairwise comparison. As expected, there were a large number of DEGs identified by FP (1563 DEGs) and LP (1758 DEGs) pairwise comparisons. Among these DEGs, 947 DEGs overlapped between contrasts (Fig. 2). By pairwise comparison, 616 DEGs were exclusive to the ampulla and 811 DEGs were exclusive to the isthmus. The complete list of these transcripts is provided in Additional file 3: Table S1 (DEGs exclusive to the ampulla or isthmus) and Additional file 4: Table S2 (DEGs in common among the ampulla and isthmus).

**Table 2:** Number of differentially expressed genes (DEGs) in epithelial cells of the ampulla and isthmus between follicular and luteal phase groups (FDR adjusted P < 0.05)

Parameter	DEGs	Up-regulated	Down-regulated
FP vs. LP (Ampulla)	1563	968 (62 %)	595 (38 %)
FP vs. LP (Isthmus)	1758	943 (54 %)	815 (46 %)

Heifers in the follicular phase group (*n* = 3) were treated with 25 mg PGF2_α_ at 8 pm on Day 6 of the estrous cycle and killed 36 h later. Heifers in the luteal phase group (*n* = 3) were killed on Day 11 or 12 of the estrous cycle. For statistical analysis, an F-test on least-square means was used to determine significance of each transcript in each comparison. Benjamini-hochberg multiple testing correction for false discovery rate (FDR) was applied and significance set to FDR adjusted *P*-value < 0.05

### DEGs in epithelial cells of the ampulla

**Fig. 3:**
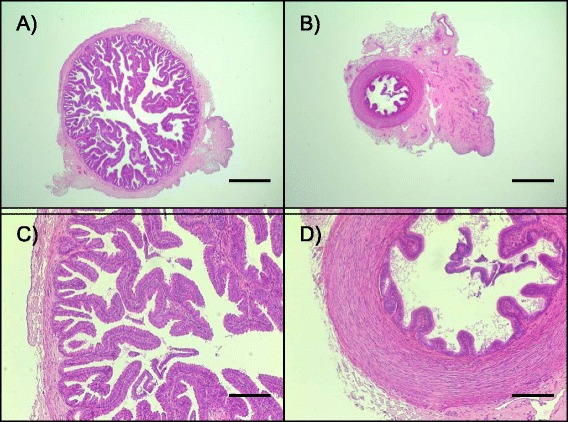
Representative images of the ampulla (**a**,**c**) and isthmus (**b**,**d**) of the bovine oviduct. Scale bar: A,B = 1000 μm; C,D = 250 μm. Images are from one heifer collected during the follicular phase of the estrous cycle. Sections were stained with hematoxylin and eosin

The ampulla is situated immediately below the ovarian bursa and infundibulum and is the site of fertilization. Histological evaluations have shown the ampulla to have an elaborate, extensively folded epithelial layer adjacent to a thin layer of smooth muscle (Fig. 3a). Evaluation of the oviducts of cattle [14, 39, 40] confirm that this is a dynamic tissue with multiple, ongoing biological processes that include cell proliferation [7] and tissue remodeling [41]. The most highly up- and down-regulated DEGs within epithelial cells of the ampulla in FP versus LP groups are provided as Table 3.

**Table 3:** Most highly up- and down-regulated DEGs within epithelial cells of the ampulla in follicular versus luteal phase groups (FDR adjusted *P* < 0.05)

Gene Symbol	Gene description	*P*-value	Fold-Change
NTS	Neurotensin	<0.001	32.44
PRND	prion protein 2 (dublet)	<0.001	18.30
CDC20B	cell division cycle 20 homolog B (S. cerevisiae)	<0.001	17.49
bTrappin-5	trappin 5	<0.001	11.14
TMEM45A	transmembrane protein 45A	0.004	10.69
CRELD2	cysteine-rich with EGF-like domains 2	<0.001	9.98
SLC2A10	solute carrier family 2 (facilitated glucose transporter), member 10	<0.001	9.54
SDF2L1	stromal cell-derived factor 2-like 1	<0.001	9.29
MIR449C	microRNA mir-449c	<0.001	9.20
KRT23	keratin 23 (histone deacetylase inducible)	0.004	8.40
DNAJB11	DnaJ (Hsp40) homolog, subfamily B, member 11	<0.001	6.92
MANF	mesencephalic astrocyte-derived neurotrophic factor	<0.001	6.90
MFSD2A	major facilitator superfamily domain containing 2A	<0.001	6.70
CLPH	calcium-binding protein, spermatid-specific 1 (CABS1)	<0.001	6.61
PLA2G4D	phospholipase A2, group IVD (cytosolic)	<0.001	6.61
CBLN4	cerebellin 4 precursor	<0.001	6.57
SERPINE3	serpin peptidase inhibitor, clade E (nexin, plasminogen activator inhibitor type 1), member 3	<0.001	6.54
RRM2	ribonucleotide reductase M2	<0.001	6.52
H4	histone H4	<0.001	6.30
INSIG1	insulin induced gene 1	<0.001	6.21
Gene Symbol	Gene Description	*P*-value	Fold-Change
EGR1	early growth response 1	0.005	−9.13
FOS	FBJ murine osteosarcoma viral oncogene homolog	0.002	−8.55
VCAM1	vascular cell adhesion molecule 1	<0.001	−7.11
GABRP	gamma-aminobutyric acid (GABA) A receptor, pi	<0.001	−6.76
GPR174	G protein-coupled receptor 174	0.003	−5.15
ZBTB16	zinc finger and BTB domain containing 16	0.001	−4.62
BMP4	bone morphogenetic protein 4	0.001	−4.44
LOC777601	uncharacterized LOC777601	<0.001	−4.37
FIGF	c-fos induced growth factor (vascular endothelial growth factor D)	<0.001	−4.12
KLRK1	killer cell lectin-like receptor subfamily K, member 1	0.003	−4.09
PDK4	pyruvate dehydrogenase kinase, isozyme 4	0.001	−3.93
LOC100297676	C-type lectin domain family 2 member G-like	0.003	−3.78
LOC100337183	contactin associated protein-like 3-like	0.008	−3.71
LOC768255	GTPase, IMAP family member 4-like	0.001	−3.64
TRAT1	T cell receptor associated transmembrane adaptor 1	0.007	−3.48
CAPN6	calpain 6	0.007	−3.48
CAV1	caveolin 1, caveolae protein, 22 kDa	0.005	−3.44
LOC541007	Similar to protein jade-2(PHD finger protein 15) (BT24231-RA)	<0.001	−3.34
CHRDL1	chordin-like 1	0.002	−3.31
MIR29C	microRNA mir-29c	0.001	−3.29

For statistical analysis, an *F*-test on least-square means was used to determine significance of each transcript in each comparison. Benjamini-hochberg multiple testing correction for false discovery rate 2 (FDR) was applied and significance set to FDR adjusted *P*-value < 0.05. A post-hoc pairwise comparison of FP compared to LP epithelial cells from the ampulla was completed using Fisher’s Least Significant Difference (LSD) to determine which means differed

As indicated above, a detailed discussion of the role for individual genes identified by this analysis is not our objective, however examples of consistency among our profiling results and more targeted analyses performed by others is warranted. Of the up-regulated genes, the expression of mRNA for neurotensin (NTS) was found to increase by 32.4-fold within epithelial cells of the ampulla in the FP versus the LP groups. Neurotensin has multiple functions and evidence suggests that NTS plays a role in gamete and embryo transport within the oviduct [42]. Receptors for NTS are expressed on spermatozoa, and increasing NTS administration facilitates sperm protein tyrosine phosphorylation, which is a measure of sperm capacitation [43]. Furthermore, the acrosome reaction is promoted in capacitated spermatozoa in the presence of increasing concentrations of NTS [43], consistent with the increased expression of NTS observed in the follicular phase herein. In contrast to this established role of NTS, and consistent with our objective to identify novel genes that may be revealed as critical mediators of function in the future, the transcription factor early growth response 1 (EGR1) was observed to display the highest fold-change (9.1-fold) among down-regulated genes within epithelial cells of the ampulla in the FP versus the LP groups. A gonadotropin-dependent induction of EGR1 has been reported prior to ovulation in bovine follicles [44], however no reports to date are apparent on the function, or importance, of this transcription factor in the epithelium of the oviducts’ ampulla.

Up- and down-regulated DEGs were analyzed for enriched gene ontology classifications. Gene ontology analysis of up-regulated DEGs within epithelial cells of the ampulla in the FP versus the LP groups resulted in 117 significant biological processes, 65 significant cellular components, and 46 significant molecular functions (*P* < 0.05,

Additional file 5: Table S3). Cell cycle, cholesterol biosynthetic process, cell division, mitosis and protein folding were the top biological processes identified, which is not surprising considering the cell proliferation and secretory activity required to prepare the ampulla for the arrival of the gametes. With respect to cholesterol biosynthesis, steroid-dependent effects on the oviduct are documented in the literature. Cholesterol will affect the ability of spermatozoa to fertilize an oocyte [45], with the process of capacitation well established to require efflux of cholesterol from the plasma membrane of spermatozoa (reviewed in [46]). High density lipoproteins are elevated in bovine oviductal fluid during the follicular phase of the estrous cycle [47], however the synthesis and release of cholesterol by oviductal epithelial cells appears to be greater under a progesterone dominant environment. Esterified-cholesterol containing lipid droplets from oviductal epithelial cells were observed in greater numbers when collected from luteal-phase cows [48] and the concentration of cholesterol was increased in isthmic but not ampullary oviductal fluid collected from the luteal phase versus non-luteal phase animals [49]. Further analysis of the biochemical relationship among the DEGs expressed within these top cholesterol-associated pathways may increase our understanding of the role of cholesterol during the processes of capacitation and fertilization, critical events that occur within the oviduct. Top cellular components of up-regulated DEGs within epithelial cells of the ampulla in the FP versus the LP groups included endoplasmic reticulum lumen and membrane, cytoplasm, and mitochondrion. Top molecular functions involved protein disulfide isomerase activity, FK506 binding, peptidyl-prolyl cis-trans isomerase activity, oxidoreductase activity, and dolichyl-diphosphooligosaccharide-protein glycotransferase activity.

Analysis of down-regulated DEGs within epithelial cells of the ampulla in the FP versus the LP revealed 118 significant biological processes, 18 significant cellular components, and 49 significant molecular functions (P < 0.05, Additional file 6: Table S4). The top biological processes were the innate immune response, response to nicotine, myoblast proliferation, negative regulation of MAP kinase activity, and bone morphogenetic protein (BMP) signaling pathway. With respect to immune responses, it can be postulated that increases in estradiol during the follicular phase decrease the induction of pro-inflammatory factors, with the results of our study consistent with an investigation on the effect of ovarian steroids on lipopolysaccharide (LPS)-induced responses in bovine oviductal epithelial cells in vitro [39]. In their report, estradiol reversed the effect of LPS on pro-inflammatory gene expression and we have previously demonstrated ESR1-dependent as well as cyclic changes in the expression of the hematopoetic form of prostaglandin D synthase, a putative regulator of inflammation, in the mouse oviduct [29]. Given that the oviductal epithelium must continually repair itself from any damage caused by exposure to gametes, seminal fluids and post-ovulatory follicular debris, steroid-dependent changes in inflammatory response mechanisms can be considered physiologically important biological processes. Lastly, top cellular components associated with the down-regulated DEGs were plasma membrane, cytoplasm, extracellular region, cytosol, and tight junction and top molecular functions were pancreatic ribonuclease activity, transmembrane signaling receptor activity, protein-L-isoaspartate (D-aspartate) O-methyltransferase activity, serine hydrolase activity, and phosphatidylinositol-3,4-bisphosphate binding.

Canonical pathway analysis of DEGs within epithelial cells of the ampulla in the FP versus the LP was then determined using QIAGEN’S Ingenuity Pathway Analysis (IPA, QIAGEN, Redwood City, www.qiagen.com/ingenuity). Top pathways up-regulated in the follicular phase largely reflected cholesterol biosynthesis (Superpathway of Cholesterol Biosynthesis, Cholesterol Biosynthesis I, Cholesterol Biosynthesis II (via 24,25-dihydrolanosterol), Cholesterol Biosynthesis III (via Desmosterol)), and Oxidative Phosphorylation (P < 0.05, Additional file 7: Table S5 and Additional file 8: Figure S3a), which was consistent with the results of the gene ontology analysis discussed above. The top pathways for down-regulated genes included Hepatic Fibrosis/Hepatic Stellate Cell Activation, Role of Pattern Recognition Receptors in Recognition of Bacteria and Viruses, Colorectal Cancer Metastasis Signaling, Ovarian Cancer Signaling, and Role of Macrophages, Fibroblasts and Endothelial Cells in Rheumatoid Arthritis (P < 0.05, Additional file 9: Table S6 and Additional file 8: Figure S3b). Again, this canonical pathway analysis is consistent with steroid-dependent regulation of inflammation and immune responses extracted by the gene ontology analysis. Worthy to note, given the increasing interest in oviductal epithelial cells as progenitors for ovarian cancer [50–52], down-regulation of the DEGs in the ovarian cancer signaling canonical pathway (MMP7, ARRB1, FZD4, FGF9, SMO, FIGF, CCND1, PDGFC, FZD7, EGFR and BCL2) could provide useful clues that can advance that important field of study. In addition, potential effects of the presence of embryos to the oviductal epithelium should not be overlooked. We observed down-regulation of 7 DEGs, including that of insulin-like growth factor binding protein 3 (IGFBP3) in epithelial cells of the ampulla in FP versus LP groups (Additional file 4: Table S2), whose expression in primary bovine oviductal epithelial cells in vitro is increased by the addition of embryos to their culture [53].

### DEGs in epithelial cells of the isthmus

Similar to the ampulla, the mucosa of the isthmus appears well defined in histological evaluations (Fig. 3b). However, in contrast to the ampulla, the mucosa of the isthmus is located adjacent to a prominent musculature with this smooth musculature playing a key role in the movement of gametes to the site of fertilization and passage of the early embryo to the uterus [54–56]. Several of the DEGs within epithelial cells of the isthmus in the FP versus the LP were similar to those identified within the ampulla, however, there were also many DEGs unique to this spatial location (Additional file 3: Tables S1 and Additional file 4: Table S2). Among the up-regulated DEGs in the epithelium of the isthmus (Table 4), the increased expression of mRNA encoding phospholipase A2, group IVD (cytosolic) (PLA2G4D) and phospholipase A2, group IVF (PLA2G4F) is interesting, especially when considering the key role for prostaglandins within the oviduct [55, 57, 58] and the noted regulation of prostaglandin synthesis by ovarian steroids as reported by us [29] and others [59, 60]. Both PLA2G4D and PLA2G4F belong to the phospholipase A2 (PLA2) family, group 4. The PLA2 family of enzymes catalyzes the hydrolysis of phospholipids to liberate free fatty acids, among other molecules. Of these free fatty acids, arachidonic acid released by PLA2 enzymes acts as the precursor for the synthesis of prostaglandins [61, 62]. Consistent with our results, in ovariectomized rabbits, PLA2 activity in epithelial cells of the ampulla is reported to be increased after treatment of rabbits with estradiol [63]. Given that PLA2G4D and PLA2G4F do not appear to be described within the oviduct, our results can be considered as new information on the local regulation of phospholipases and potentially prostaglandin secretion within this organ. Of the down-regulated DEGs expressed by epithelial cells of the isthmus, the expression of binder of sperm 3 (BSP3) is also identified as a novel transcript. BSP3 is reported to be secreted by seminal vesicles and binds to sperm [64], and identified within the epithelial cells of the isthmus herein. Again, the potential to modify the epithelial cell transcriptome by the presence of embryos must be acknowledged. We observed down-regulation of 10 DEGs, including that of the apoptosis regulator XIAP associated factor-1 (XAF1) in epithelial cells of the isthmus in FP versus LP groups (Additional file 4: Table S2), whose expression has been reported to be increased by the addition of embryos to cultures of primary bovine oviductal epithelial cells [53].

**Table 4:** Most highly up- and down-regulated DEGs within epithelial cells of the isthmus in follicular phase versus luteal phase groups (FDR adjusted *P* < 0.05)

Gene Symbol	Gene Description	*P*-value	Fold-Change
KRT23	keratin 23 (histone deacetylase inducible)	<0.001	22.57
NTS	neurotensin	<0.001	21.57
PRND	prion protein 2 (dublet)	<0.001	12.36
STRA6	stimulated by retinoic acid gene 6 homolog (mouse)	<0.001	11.22
TMEM45A	transmembrane protein 45A	0.004	10.56
PKHD1L1	polycystic kidney and hepatic disease 1 (autosomal recessive)-like 1	0.004	9.86
LPL	lipoprotein lipase	<0.001	9.40
SLC7A11	solute carrier family 7 (anionic amino acid transporter light chain, xc- system), member 11	<0.001	8.92
CLEC3A	C-type lectin domain family 3, member A	<0.001	8.69
GFAP	glial fibrillary acidic protein	<0.001	8.09
CRELD2	cysteine-rich with EGF-like domains 2	<0.001	7.69
CA2	carbonic anhydrase II	<0.001	7.68
PLA2G4D	phospholipase A2, group IVD (cytosolic)	<0.001	7.64
CHI3L1	chitinase 3-like 1 (cartilage glycoprotein-39)	0.001	7.34
SDF2L1	stromal cell-derived factor 2-like 1	<0.001	7.31
PPP2R2C	protein phosphatase 2, regulatory subunit B, gamma	0.000	7.30
P2RX2	purinergic receptor P2X, ligand-gated ion channel, 2	0.002	6.86
PLA2G4F	phospholipase A2, group IVF	<0.001	6.85
SLC2A10	solute carrier family 2 (facilitated glucose transporter), member 10	<0.001	6.69
CDC20B	cell division cycle 20 homolog B (S. cerevisiae)	0.001	6.35
Gene Symbol	Gene Description	*P*-value	Fold-Change
KLF17	Kruppel-like factor 17	<0.001	−14.70
KSR2	kinase suppressor of ras 2	<0.001	−8.94
LOC100337391	predicted protein-like	<0.001	−7.77
BSP3	binder of sperm 3	<0.001	−7.58
OR9Q2	olfactory receptor, family 9, subfamily Q, member 2	0.001	−6.87
ZBTB16	zinc finger and BTB domain containing 16	<0.001	−5.91
EGR1	early growth response 1	0.019	−5.35
LOC522479	ovalbumin-like	<0.001	−5.34
CWH43	cell wall biogenesis 43 C-terminal homolog (S. cerevisiae)	0.001	−5.09
TFF3	trefoil factor 3 (intestinal)	<0.001	−4.98
LOC100335668	mitochondrial import inner membrane translocase subunit Tim9 pseudogene	0.001	−4.94
LOC617981	family with sequence similarity 55, member C-like	<0.001	−4.92
MEGF10	multiple EGF-like-domains 10	<0.001	−4.81
LOC100337183	contactin associated protein-like 3-like	0.003	−4.75
AK5	adenylate kinase 5	<0.001	−4.74
FOS	FBJ murine osteosarcoma viral oncogene homolog	0.012	−4.54
CA10	carbonic anhydrase X	0.001	−4.40
NDP	Norrie disease (pseudoglioma)	<0.001	−4.34
SEMA5A	sema domain, seven thrombospondin repeats (type 1 and type 1- like), transmembrane domain (TM) and short cytoplasmic domain, (semaphorin) 5A	<0.001	−4.26
CAV1	caveolin 1, caveolae protein, 22 kDa	0.003	−4.14

For statistical analysis, an F-test on least-square means was used to determine significance of each transcript in each comparison. Benjamini-hochberg multiple testing correction for false discovery rate (FDR) was applied and significance set to FDR adjusted *P*-value < 0.05. A post-hoc pairwise comparison of FP compared to LP epithelial cells from the isthmus was completed using Fisher’s Least Significant Difference (LSD) to determine which means differed

Gene ontology classification of up-regulated DEGs within epithelial cells of the isthmus in the FP versus the LP revealed 97 significant biological processes with top biological processes being protein folding, cell cycle, cell division, mitosis, and electron transport chain (*P* < 0.05, Additional file 10: Table S7). Similar to the ampulla, there were 57 significant cellular components affected by stage of the cycle within epithelial cells of the isthmus, including endoplasmic reticulum lumen and membrane, cytoplasm, and mitochondrion and 46 significant molecular functions. Gene ontology analysis of the down-regulated DEGs within epithelial cells of the isthmus revealed 101 significant biological processes, 26 significant cellular components, and 45 significant molecular functions (P < 0.05, Additional file 11: Table S8). Top biological processes were negative regulation of fibroblast growth factor receptor signaling pathway, calcium-independent cell-cell adhesion, inactivation of MAPK activity, brown fat cell differentiation, and SMAD protein signal transduction. Top cellular components were similar to those observed within the ampulla and included cytoplasm, plasma membrane cytosol, extracellular region, and tight junction.

Again, QIAGEN’S Ingenuity Pathway Analysis (IPA, QIAGEN, Redwood City, www.qiagen.com/ingenuity) was used to identify the canonical pathways affected by stage of the cycle within epithelial cells of the isthmus. Up-regulated DEGs within epithelial cells of the isthmus in FP versus LP were oxidative phosphorylation, mitochondrial dysfunction, superpathway of cholesterol biosynthesis, superpathway of geranylgeranyldiphosphate biosynthesis I (via mevalonate), and melavonate pathway I (P < 0.05, Additional file 12: Table S9 and Additional file 13: Figure S4A). Similar to that observed in the ampulla, cholesterol biosynthesis stands out as a key pathway with gene expression increased under an estrogen dominant environment. Consistency in response was also observed among down-regulated DEGs; top pathways for down-regulated DEGs included molecular mechanisms of cancer, basal cell carcinoma signaling, role of osteoblasts, osteoclasts and chondrocytes in rheumatoid arthritis, Role of Macrophages, Fibroblasts and Endothelial Cells in Rheumatoid Arthritis, and Corticotropin Releasing Hormone Signaling, with the complete listing provided in Additional file 14: Table S10 and Additional file 13: Figure S4B (*P* < 0.05).

## Conclusions

At the genome level, estrous cycle stage-dependent effects on epithelial cell gene expression are not well defined. The current study therefore investigated changes in the expression of mRNA within the epithelium of the ampulla and isthmus of the bovine oviduct during the luteal and follicular phases of the estrous cycle. This transcriptomal profiling analysis was performed to increase our understanding of gene expression and potentially epithelial cell processes important for oviductal function and fertility, and to identify novel mRNA that may prove critical for fertility after analysis in the future.

## Additional files

Additional file 1:
**Supplementary Figure 1.** Box plot of the log2 expression signal for each sample (microarray chip). Additional file 2:
**Supplementary Figure 2.** Overlapping sample signal intensity histogram indicating the frequency of transcripts at specific signal intensity values. Additional file 3:
**Supplementary Table 1.** Differentially expressed transcripts between follicular and luteal phases of the estrous cycle in epithelial cells exclusive to the ampulla and isthmus (FDR adjusted P < 0.05). Additional file 4:
**Supplementary Table 2.** Differentially expressed transcripts in common between follicular and luteal phases of the estrous cycle in epithelial cells of the ampulla and isthmus (FDR adjusted P < 0.05). Additional file 5:
**Supplementary Table 3.** Enriched gene ontology classifications from up-regulated DEGS within epithelial cells of the ampulla in follicular versus luteal phase groups (P < 0.05). Additional file 6:
**Supplementary Table 4.** Enriched gene ontology classifications from down-regulated DEGS within epithelial cells of the ampulla in follicular versus luteal phase groups (P < 0.05). Additional file 7:
**Supplementary Table 5.** Canonical pathway analysis of up-regulated DEGS within epithelial cells of the ampulla in the follicular versus luteal phase groups (P < 0.05). Additional file 8:
**Supplementary Figure 3.** Top 6 Canonical pathways from up- and down-regulated differentially expressed genes within epithelial cells of the ampulla in the follicular versus luteal phase. Ingenuity Pathway Analysis software was used to determine significant pathways based on the number of significant genes expressed within the pathway using Fischer’s Exact test (P <0.05). A) Ampulla: Up-regulated pathways in the follicular versus luteal phases. B) Ampulla: Down-regulated pathways in the follicular versus luteal phases. Additional file 9:
**Supplementary Table 6.** Canonical pathway analysis of down-regulated DEGS within epithelial cells of the ampulla in the follicular versus luteal phase groups (P < 0.05). Additional file 10:
**Supplementary Table 7.** Enriched gene ontology classifications from up-regulated DEGS within epithelial cells of the isthmus in follicular versus luteal phase groups (P < 0.05). Additional file 11:
**Supplementary Table 8.** Enriched gene ontology classifications from down-regulated DEGS within epithelial cells of the isthmus in follicular versus luteal phase groups (P < 0.05). Additional file 12:
**Supplementary Table 9.** Canonical pathway analysis of up-regulated DEGS within epithelial cells of the isthmus in the follicular versus luteal phase groups (P < 0.05). Additional file 13:
**Supplementary Figure 4.** Top 6 Canonical pathways from up- and down-regulated differentially expressed genes within epithelial cells of the isthmus in the follicular versus luteal phase. Ingenuity Pathway Analysis software was used to determine significant pathways based on the number of significant genes expressed within the pathway using Fischer’s Exact test (P <0.05). A) Isthmus: Up-regulated pathways in the follicular versus luteal phases. B) Isthmus: Down-regulated pathways in the follicular versus luteal phases. Additional file 14:
**Supplementary Table 10.** Canonical pathways of down-regulated DEGs within epithelial cells of the isthmus in the follicular versus luteal phase groups (P < 0.05).

## Abbreviations

ANOVA: Analysis of variance
DEGs: Differentially expressed genes
FDR: False discovery rate
FP: Follicular phase
LP: Luteal phase
mRNA: messenger RNA
