# Supplementary material for: A transcriptomal analysis of bovine oviductal epithelial cells collected during the follicular phase versus the luteal phase of the estrous cycle
Source: Reprod Biol Endocrinol. 2015 Aug 5;13:84. doi: 10.1186/s12958-015-0077-1 (PMC4524109; doi:10.1186/s12958-015-0077-1)

Supplementary Figure 1. Box plot of the $\log_2$ expression signal for each sample (microarray chip).

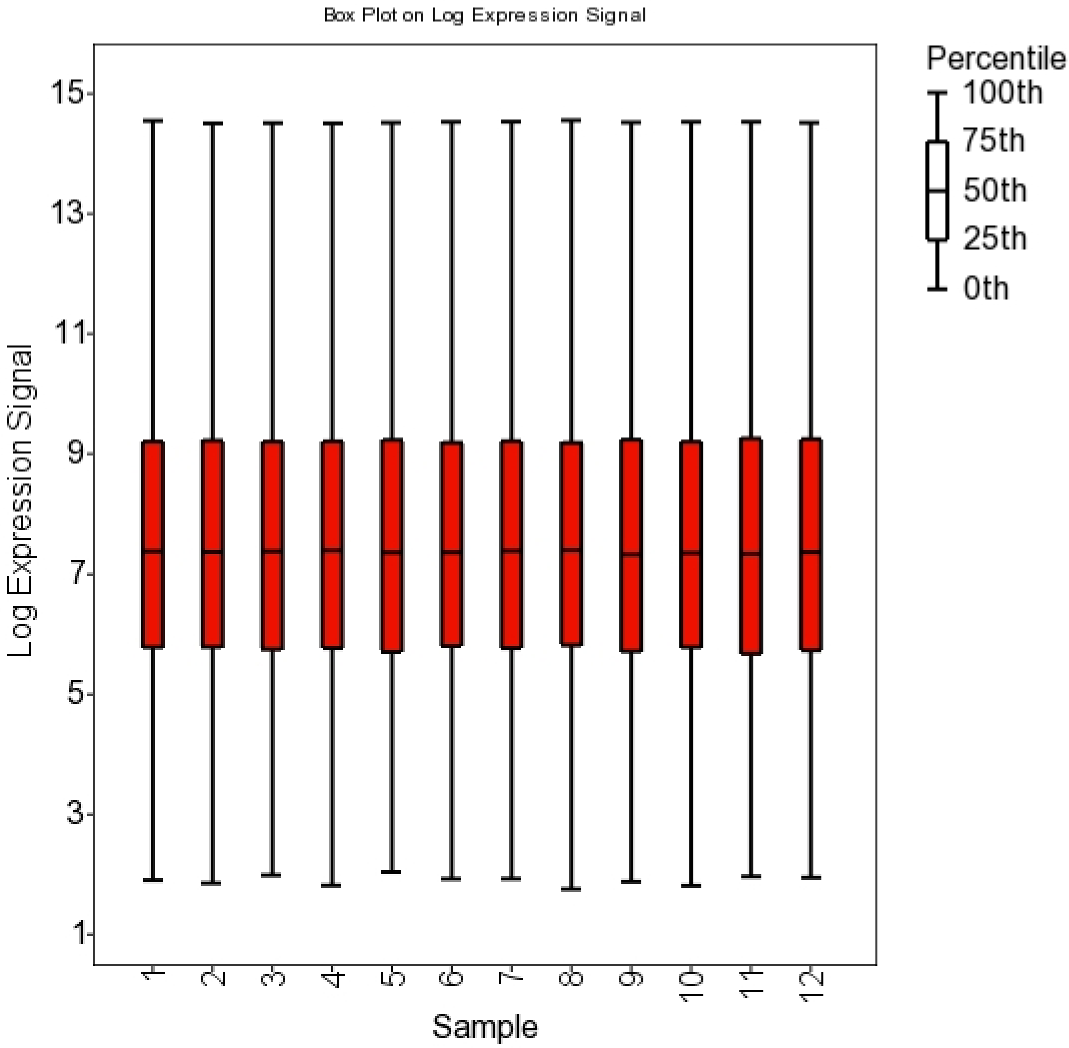

Supplement: Additional file 1: — Supplementary Figure 1. Box plot of the log2 expression signal for each sample (microarray chip). [file 12958_2015_77_MOESM1_ESM.pdf]
