# Supplementary material for: A transcriptomal analysis of bovine oviductal epithelial cells collected during the follicular phase versus the luteal phase of the estrous cycle
Source: Reprod Biol Endocrinol. 2015 Aug 5;13:84. doi: 10.1186/s12958-015-0077-1 (PMC4524109; doi:10.1186/s12958-015-0077-1)

Supplementary Figure 2. Overlapping sample signal intensity histogram indicating the frequency of transcripts at specific signal intensity values.

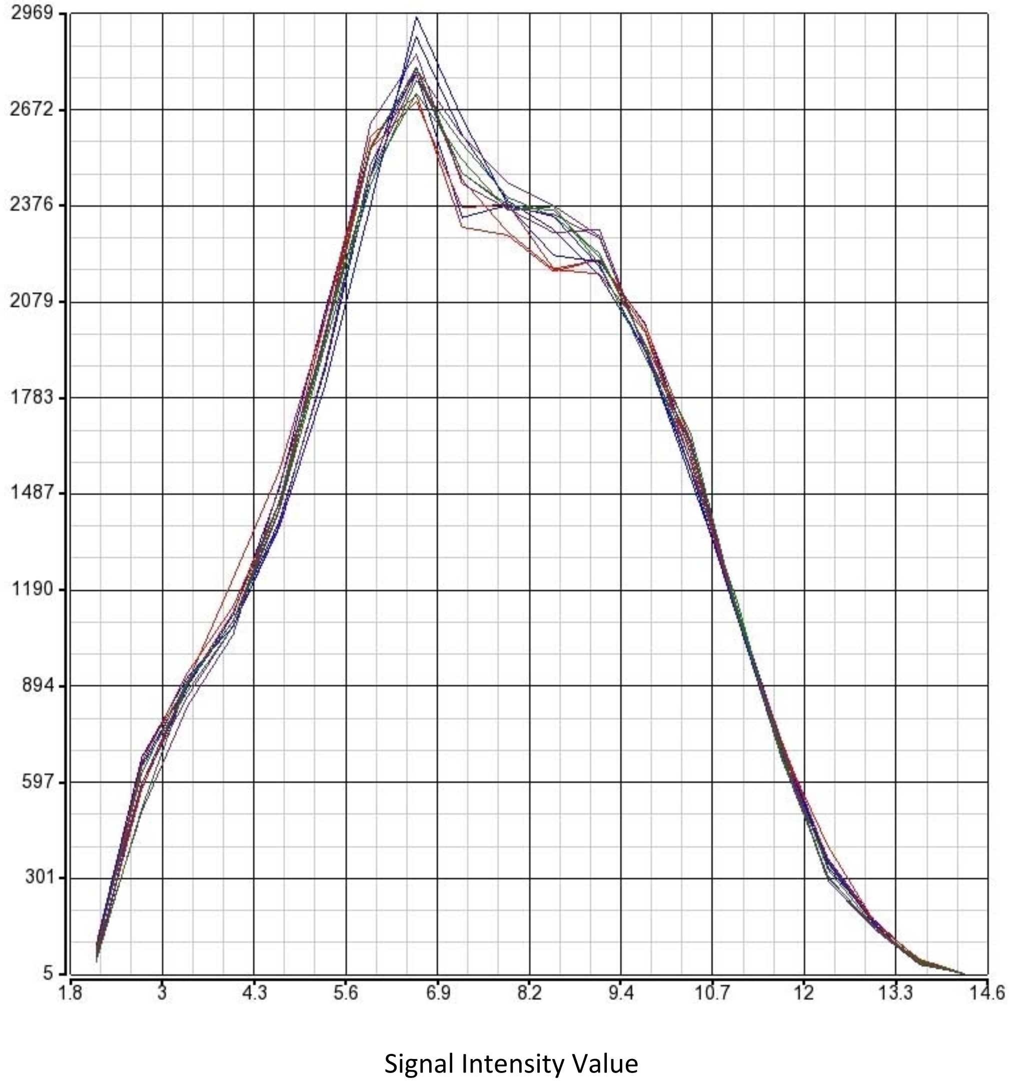

Supplement: Additional file 2: — Supplementary Figure 2. Overlapping sample signal intensity histogram indicating the frequency of transcripts at specific signal intensity values. [file 12958_2015_77_MOESM2_ESM.pdf]
