# Supplementary material for: A transcriptomal analysis of bovine oviductal epithelial cells collected during the follicular phase versus the luteal phase of the estrous cycle
Source: Reprod Biol Endocrinol. 2015 Aug 5;13:84. doi: 10.1186/s12958-015-0077-1 (PMC4524109; doi:10.1186/s12958-015-0077-1)
Supplement: Additional file 3: — Supplementary Table 1. Differentially expressed transcripts between follicular and luteal phases of the estrous cycle in epithelial cells exclusive to the ampulla and isthmus (FDR adjusted P < 0.05). [file 12958_2015_77_MOESM3_ESM.pdf]

Supplementary Table 1. Differentially expressed transcripts between follicular and luteal phases of the estrous cycle in epithelial cells exclusive to the ampulla and isthmus (FDR adjusted P < 0.05).

FP vs. LP (Ampulla)			
**Gene Symbol**	**Gene Description**	**P-value**	**Fold-Change**
A4GALT	alpha 1,4-galactosyltransferase	0.009	1.81
AAAS	achalasia, adrenocortical insufficiency, alacrimia	< 0.001	1.63
AANAT	arylalkylamine N-acetyltransferase	< 0.001	1.80
AARS	alanyl-tRNA synthetase	< 0.001	1.53
AASS	aminoadipate-semialdehyde synthase	0.002	-1.60
ABCB6	ATP-binding cassette, sub-family B (MDR/TAP), member 6	< 0.001	1.70
ABCB8	ATP-binding cassette, sub-family B (MDR/TAP), member 8	0.002	1.61
ABCC8	ATP-binding cassette, sub-family C (CFTR/MRP), member 8	0.041	2.16
ABHD12B	abhydrolase domain containing 12B	0.002	-1.52
ACACA	acetyl-CoA carboxylase alpha	< 0.001	1.71
ACRC	acidic repeat containing	0.001	-1.56
ACSL3	acyl-CoA synthetase long-chain family member 3	< 0.001	1.96
ACSS2	acyl-CoA synthetase short-chain family member 2	0.001	1.87
ACTL6A	actin-like 6A	< 0.001	1.55
ACTR1A	ARP1 actin-related protein 1 homolog A, centractin alpha (yeast)	< 0.001	1.56
ADHFE1	alcohol dehydrogenase, iron containing, 1	0.003	-1.52
ADSL	adenylosuccinate lyase	< 0.001	1.58
AFAP1L2	actin filament associated protein 1-like 2	< 0.001	-1.60
AGRN	agrin	< 0.001	-1.53
AIMP2	aminoacyl tRNA synthetase complex-interacting multifunctional protein 2	< 0.001	1.64
AKAP12	A kinase (PRKA) anchor protein 12	0.042	-1.64
ALDOC	aldolase C, fructose-bisphosphate	< 0.001	2.14
ALPK1	alpha-kinase 1	< 0.001	-1.79
ALS2CR12	amyotrophic lateral sclerosis 2 (juvenile) chromosome region, candidate 12	0.001	-1.57
AMACR	alpha-methylacyl-CoA racemase	0.015	1.55
AMY2A	amylase, alpha 2A (pancreatic)	0.008	-1.97
AMZ2	archaelysin family metallopeptidase 2	< 0.001	1.54
ANG2	angiogenin 2	0.008	-1.68
ANKRD10	ankyrin repeat domain 10	< 0.001	-1.52
ANKRD22	ankyrin repeat domain 22	0.002	1.59
ANKRD31	ankyrin repeat domain 31	0.026	-1.54
ANKRD32	ankyrin repeat domain 32	0.001	-1.73
ANO10	anoctamin 10	< 0.001	1.60
ANO5	anoctamin 5	0.003	-2.18
ANP32B	acidic (leucine-rich) nuclear phosphoprotein 32 family, member B	0.008	1.53

APEH	N-acylaminoacyl-peptide hydrolase	0.001	1.53
APLF	aprataxin and PNKP like factor	< 0.001	-1.62
APOA1BP	apolipoprotein A-I binding protein	< 0.001	1.60
APOL3	apolipoprotein L, 3	0.008	-1.50
AQP9	aquaporin 9	0.007	-1.84
ARFGAP3	ADP-ribosylation factor GTPase activating protein 3	< 0.001	1.71
ARFIP2	ADP-ribosylation factor interacting protein 2	< 0.001	1.81
ARHGAP15	Rho GTPase activating protein 15	0.005	-3.08
ARHGAP29	Rho GTPase activating protein 29	0.007	-1.84
ARHGEF12	Rho guanine nucleotide exchange factor (GEF) 12	< 0.001	1.52
ARL2	ADP-ribosylation factor-like 2	< 0.001	1.82
ARRB1	arrestin, beta 1	0.002	-1.67
ARSB	arylsulfatase B	< 0.001	1.76
ASPH	aspartate beta-hydroxylase	0.001	1.57
ATAD3A	ATPase family, AAA domain containing 3A	< 0.001	1.59
ATF4	activating transcription factor 4 (tax-responsive enhancer element B67)	< 0.001	1.73
ATF7IP2	activating transcription factor 7 interacting protein 2	0.038	2.32
ATP13A1	ATPase type 13A1	< 0.001	1.50
ATPBD4	ATP binding domain 4 (DPH6)	0.002	1.54
AURKA	aurora kinase A	0.001	1.95
AURKB	aurora kinase B	0.001	1.91
AVPI1	arginine vasopressin-induced 1	0.003	1.55
BACH2	BTB and CNC homology 1, basic leucine zipper transcription factor 2	0.001	-1.75
BAG3	BCL2-associated athanogene 3	0.012	1.55
BHLHB9	basic helix-loop-helix domain containing, class B, 9	0.006	-2.04
BHLHE41	basic helix-loop-helix family, member e41	0.003	-1.57
BID	BH3 interacting domain death agonist transcript-like	0.001	1.62
BLM	Bloom syndrome, RecQ helicase-like	0.001	1.60
BOSTAUV1 R402	vomeronasal 1 receptor bosTauV1R402	0.015	-1.50
BRI3BP	BRI3 binding protein	< 0.001	1.69
BTBD11	BTB (POZ) domain containing 11	0.004	-1.61
BTC	betacellulin	< 0.001	-1.57
bTrappin-5	trappin 5	< 0.001	11.14
BVD1.15	T cell receptor delta chain variable region BVd1.15	0.010	-1.52
BZW2	basic leucine zipper and W2 domains 2	< 0.001	1.62
C13H20ORF 108	family with sequence similarity 210, member B(FAM210B)	0.001	-1.77
C15H11orf5 9	late endosomal/lysosomal adaptor, MAPK and MTOR activator 1 (LAMTOR1)	< 0.001	1.53
C16H1ORF2 6	SWT1 RNA endoribonuclease homolog (S. cerevisiae)(SWT1)	< 0.001	-1.51

C18H19ORF63	chromosome 18 open reading frame (EMC10)	0.003	1.58
C27H8orf4	chromosome 27 open reading frame	0.044	-1.79
C5H12orf23	chromosome 5 open reading frame	< 0.001	1.68
C5H12orf32	chromosome 5 open reading frame (RHNO1)	< 0.001	1.79
C6H4orf34	small integral membrane protein 14(SMIM14)	0.002	1.65
C6H4orf36	chromosome 6 open reading frame	0.002	2.08
C7H19ORF43	chromosome 7 open reading frame	< 0.001	1.54
C7H19orf50	KxDL motif containing 1(KXD1)	0.011	2.07
C7H19orf70	chromosome 7 open reading frame	< 0.001	1.53
C9H6ORF115	ABRA C-terminal like(ABRACL)	0.009	1.74
CA9	carbonic anhydrase IX	0.001	1.59
CAMK1D	calcium/calmodulin-dependent protein kinase ID	0.001	-1.65
CAPN6	calpain 6	0.007	-3.48
CAPSL	calcyphosine-like	0.004	-1.51
CASP3	caspase 3, apoptosis-related cysteine peptidase	0.002	1.60
CBLN4	cerebellin 4 precursor	< 0.001	6.57
CCDC115	coiled-coil domain containing 115	0.002	2.00
CCDC34	coiled-coil domain containing 34	0.003	1.50
CCDC76	coiled-coil domain containing 76	< 0.001	-1.63
CCDC88A	coiled-coil domain containing 88A	0.012	1.65
CCNE2	cyclin E2	0.002	3.47
CD1E	CD1e molecule	0.006	-1.92
CD320	CD320 molecule	0.020	2.30
CD59	CD59 molecule, complement regulatory protein	0.002	-1.68
CDC25A	cell division cycle 25 homolog A (S. pombe)	0.002	2.21
CDCA8	cell division cycle associated 8	< 0.001	2.35
CDK6	cyclin-dependent kinase 6	< 0.001	-1.75
CELSR2	cadherin, EGF LAG seven-pass G-type receptor 2 (flamingo homolog, Drosophila)	0.001	-1.51
CENPH	centromere protein H	0.002	1.58
CENPQ	centromere protein Q	0.018	1.54
CFAP54	cilia and flagella associated 54	< 0.001	-1.72
CFL1	cofilin 1 (non-muscle)	0.001	1.56
CGRRF1	cell growth regulator with ring finger domain 1	< 0.001	1.57
CHAF1A	chromatin assembly factor 1, subunit A (p150)	< 0.001	1.56
CHP	calcium binding protein P22(aka CHP1)	< 0.001	1.53
CIART	circadian associated repressor of transcription	0.001	-1.82
CKS1B	CDC28 protein kinase regulatory subunit 1B	0.005	1.54
CLDN1	claudin 1	0.002	-1.92
CLDN10	claudin 10	0.008	-1.80

CLEC4A	C-type lectin domain family 4, member A	< 0.001	-2.04
CLEC7A	C-type lectin domain family 7, member A	0.003	-2.05
COPG	coatomer protein complex, subunit gamma(COPG1)	< 0.001	1.68
COPZ1	coatomer protein complex, subunit zeta 1	< 0.001	1.91
COX7A1	cytochrome c oxidase subunit VIIa polypeptide 1 (muscle)	0.002	1.66
CPA4	carboxypeptidase A4	0.039	-1.62
CPSF3L	cleavage and polyadenylation specific factor 3-like	0.002	1.50
CPSF4	cleavage and polyadenylation specific factor 4, 30kDa	< 0.001	1.64
CREB3L4	cAMP responsive element binding protein 3-like 4	0.021	1.94
CRIP3	cysteine-rich protein 3	< 0.001	1.66
CROT	carnitine O-octanoyltransferase	0.006	-1.50
CRTAP	cartilage associated protein	0.002	1.52
CSDC2	cold shock domain containing C2, RNA binding	< 0.001	-1.71
CSNK1G2	casein kinase 1, gamma 2	< 0.001	1.58
CSPG5	chondroitin sulfate proteoglycan 5 (neuroglycan C)	0.002	1.91
CTDSP2	CTD (carboxy-terminal domain, RNA polymerase II, polypeptide A) small phosphatase 2	< 0.001	-1.62
CTSS	cathepsin S	0.001	-1.50
CX3CR1	chemokine (C-X3-C motif) receptor 1	0.009	-2.13
CXCL2	chemokine (C-X-C motif) ligand 2	0.001	-1.56
CYB561D2	cytochrome b-561 domain containing 2	0.002	1.52
CYB5B	cytochrome b5 type B (outer mitochondrial membrane)	< 0.001	1.73
CYCS	cytochrome c, somatic	0.001	1.88
CYP27A1	cytochrome P450, family 27, subfamily A, polypeptide 1	0.002	1.54
CYP51A1	cytochrome P450, family 51, subfamily A, polypeptide 1	< 0.001	3.52
CYR61	cysteine-rich, angiogenic inducer, 61	0.004	-2.52
DAPK1	death-associated protein kinase 1	< 0.001	-1.50
DAPK3	death-associated protein kinase 3	0.003	-1.53
DAPP1	dual adaptor of phosphotyrosine and 3-phosphoinositides	0.014	-1.51
DCBLD1	discoidin, CUB and LCCL domain containing 1	0.001	1.53
DCLK2	doublecortin-like kinase 2	0.002	-1.58
DDX39	DEAD (Asp-Glu-Ala-Asp) box polypeptide 39A (DDX39A)	< 0.001	1.80
DHCR24	24-dehydrocholesterol reductase	< 0.001	2.85
DHCR7	7-dehydrocholesterol reductase	< 0.001	2.21
DHFR	dihydrofolate reductase	0.002	1.88
DHODH	dihydroorotate dehydrogenase (quinone)	< 0.001	1.51
DKKL1	dickkopf-like 1	0.006	1.64
DMXL2	Dmx-like 2	0.003	-1.60
DNAH9	dynein, axonemal, heavy chain 9	< 0.001	-1.55
DNAJB2	DnaJ (Hsp40) homolog, subfamily B, member 2	< 0.001	1.51
DOCK9	dedicator of cytokinesis 9	< 0.001	-2.47

DOLPP1	dolichyl pyrophosphate phosphatase 1	< 0.001	1.99
DPM2	dolichyl-phosphate mannosyltransferase polypeptide 2, regulatory subunit	< 0.001	1.66
DPM3	dolichyl-phosphate mannosyltransferase polypeptide 3	< 0.001	1.53
DPY19L2	probable C-mannosyltransferase DPY19L2-like	0.002	-1.50
DSC2	desmocollin 2	0.037	-3.02
DTYMK	deoxythymidylate kinase (thymidylate kinase)	< 0.001	1.79
DZIP1L	DAZ interacting zinc finger protein 1-like	0.002	-1.56
DZIP3	DAZ interacting protein 3, zinc finger	< 0.001	-1.64
E2F1	E2F transcription factor 1	0.001	2.05
E2F4	E2F transcription factor 4, p107/p130-binding	< 0.001	1.51
EBP	emopamil binding protein (sterol isomerase)	< 0.001	2.22
EEF1G	eukaryotic translation elongation factor 1 gamma	< 0.001	1.52
EFEMP1	EGF containing fibulin-like extracellular matrix protein 1	0.006	-1.72
EFHD2	EF-hand domain family, member D2	0.006	1.58
EFNA1	ephrin-A1	0.005	-1.50
EGFR	epidermal growth factor receptor	< 0.001	-1.50
EIF2C4	argonaute RISC catalytic component 4 (AGO4)	< 0.001	-1.58
EIF4GI	eukaryotic translation initiation factor 4 gamma, 1	< 0.001	1.72
ELOVL6	ELOVL fatty acid elongase 6	< 0.001	1.68
EMG1	EMG1 nucleolar protein homolog (S. cerevisiae)	< 0.001	1.53
EMID1	EMI domain containing 1	< 0.001	1.96
ENTPD3	ectonucleoside triphosphate diphosphohydrolase 3	0.003	-1.67
ENTPD4	ectonucleoside triphosphate diphosphohydrolase 4	< 0.001	1.73
F2R	coagulation factor II (thrombin) receptor	0.004	-1.97
FADS1	fatty acid desaturase 1	< 0.001	1.90
FADS2	fatty acid desaturase 2	< 0.001	2.33
FAM108A	family with sequence similarity 108, member A1 (ABHA17A)	< 0.001	1.51
FAM125A	family with sequence similarity 125, member A (MVB12A)	< 0.001	1.50
FAM169A	family with sequence similarity 169, member A	0.001	-1.54
FAM43A	family with sequence similarity 43, member A	0.001	-2.52
FAM57A	family with sequence similarity 57, member A	0.001	1.74
FAM65B	family with sequence similarity 65, member B	0.002	-2.49
FAM72A	family with sequence similarity 72, member A	0.007	2.14
FAM98A	family with sequence similarity 98, member A	< 0.001	1.52
FANCB	Fanconi anemia, complementation group B	0.003	1.75
FARSA	phenylalanyl-tRNA synthetase, alpha subunit	< 0.001	1.59
FAS	Fas (TNF receptor superfamily, member 6)	< 0.001	-1.69
FAU	Finkel-Biskis-Reilly murine sarcoma virus (FBR-MuSV) ubiquitously expressed	< 0.001	1.98
FBXL21	F-box and leucine-rich repeat protein 21	< 0.001	-1.81
FCHO2	FCH domain only protein 2-like	< 0.001	-1.65

FDFT1	farnesyl-diphosphate farnesyltransferase 1	< 0.001	3.40
FGF9	fibroblast growth factor 9	0.006	-1.52
FIBP	fibroblast growth factor (acidic) intracellular binding protein	< 0.001	1.77
FIGF	c-fos induced growth factor (vascular endothelial growth factor D)	< 0.001	-4.12
FITM2	fat storage-inducing transmembrane protein 2	< 0.001	1.65
FKBP14	FK506 binding protein 14, 22 kDa	0.005	1.51
FKBP3	FK506 binding protein 3, 25kDa	< 0.001	1.52
FLT3	fms-related tyrosine kinase 3	0.004	-2.03
FLVCR1	feline leukemia virus subgroup C cellular receptor 1	0.005	1.58
FMNL2	formin-like 2	0.004	-1.67
FNDC3B	fibronectin type III domain containing 3B	< 0.001	1.56
FOXO1	forkhead box O1	0.003	-1.60
FOXS1	forkhead box S1	< 0.001	-1.57
FRAS1	Fraser syndrome 1	0.002	-2.55
FST	follistatin	< 0.001	-2.88
FUT1	fucosyltransferase 1 (galactoside 2-alpha-L-fucosyltransferase, H blood group)	0.022	2.84
FZD4	frizzled family receptor 4	0.004	-1.53
FZD7	frizzled family receptor 7	0.006	-1.78
GABRP	gamma-aminobutyric acid (GABA) A receptor, pi	< 0.001	-6.76
GALNT1	UDP-N-acetyl-alpha-D-galactosamine:polypeptide N-acetylgalactosaminyltransferase 1 (GalNAc-T1)	0.001	1.75
GALT	galactose-1-phosphate uridylyltransferase	< 0.001	1.54
GANAB	glucosidase, alpha; neutral AB	< 0.001	1.63
GAR1	GAR1 ribonucleoprotein homolog (yeast)	0.003	1.55
GAS6	growth arrest-specific 6	0.003	-1.53
GCNT1	glucosaminyl (N-acetyl) transferase 1, core 2	0.045	-1.71
GCNT4	glucosaminyl (N-acetyl) transferase 4, core 2	0.022	-2.24
GDPD5	glycerophosphodiester phosphodiesterase domain containing 5	0.001	2.06
GFER	growth factor, augmenter of liver regeneration	< 0.001	1.59
GINS1	DNA replication complex GINS protein PSF1-like	< 0.001	2.09
GIPC2	GIPC PDZ domain containing family, member 2	0.033	-2.25
GNG10	guanine nucleotide binding protein (G protein), gamma 10	0.003	1.57
GNG13	guanine nucleotide binding protein (G protein), gamma 13	0.019	1.53
GNG3	guanine nucleotide binding protein (G protein), gamma 3	0.004	1.54
GORASP1	golgi reassembly stacking protein 1, 65kDa	< 0.001	1.53
GPR110	G protein-coupled receptor 110	0.015	-1.60
GPX4	glutathione peroxidase 4	< 0.001	2.02
GRAP2	GRB2-related adaptor protein 2	0.003	-1.84
GSS	glutathione synthetase	< 0.001	1.71
H2AFZ	H2A histone family, member Z	0.001	1.52
HAVCR2	hepatitis A virus cellular receptor 2	< 0.001	-1.69

HDLBP	high density lipoprotein binding protein	< 0.001	2.00
HDX	highly divergent homeobox	0.006	1.69
HEPACAM	hepatic and glial cell adhesion molecule	0.046	-1.74
HFE	hemochromatosis	0.002	-1.51
HGF	hepatocyte growth factor (hepapoietin A; scatter factor)	0.004	-1.86
HIST1H2AD	histone cluster 1, H2	0.001	1.56
HIST1H2AG	histone cluster 1, H2ag	< 0.001	2.74
HIST1H2AJ	histone cluster 1, H2aj	0.003	1.57
HIST2H2AB	histone cluster 2, H2ab	0.001	2.30
HK2	hexokinase 2	< 0.001	2.39
HMG20B	high mobility group 20B	0.001	2.02
HMOX1	heme oxygenase (decycling) 1	< 0.001	-1.88
HPDL	4-hydroxyphenylpyruvate dioxygenase-like	0.003	2.60
HPGD	hydroxyprostaglandin dehydrogenase 15-(NAD)	0.043	-2.19
HRH1	histamine receptor H1	0.002	-1.70
ICA1	islet cell autoantigen 1, 69kDa	< 0.001	1.68
IDH1	isocitrate dehydrogenase 1 (NADP+), soluble	< 0.001	1.98
IDH2	isocitrate dehydrogenase 2 (NADP+), mitochondrial	< 0.001	1.63
IER3IP1	immediate early response 3 interacting protein 1	< 0.001	1.52
IGFBP3	insulin-like growth factor binding protein 3	0.046	-1.68
IGSF6	immunoglobulin superfamily, member 6	< 0.001	-1.54
IL15	interleukin 15	0.017	-1.51
IMP4	IMP4, U3 small nucleolar ribonucleoprotein, homolog (yeast)	0.001	1.72
IQGAP3	IQ motif containing GTPase activating protein 3	0.001	1.83
ITGB6	integrin, beta 6	< 0.001	-1.77
JMY	junction mediating and regulatory protein, p53 cofactor	< 0.001	-1.52
KCNK1	potassium channel, subfamily K, member 1	< 0.001	1.80
KCNK6	potassium channel, subfamily K, member 6	0.001	1.59
KCNRG	potassium channel regulator	< 0.001	-1.87
KDELC1	KDEL (Lys-Asp-Glu-Leu) containing 1	< 0.001	1.61
KDELR1	KDEL (Lys-Asp-Glu-Leu) endoplasmic reticulum protein retention receptor 1	< 0.001	1.51
KDELR2	KDEL (Lys-Asp-Glu-Leu) endoplasmic reticulum protein retention receptor 2	< 0.001	1.56
KDELR3	KDEL (Lys-Asp-Glu-Leu) endoplasmic reticulum protein retention receptor 3	0.002	1.54
KIF13B	kinesin family member 13B	< 0.001	-1.60
KIF22	kinesin family member 22	0.001	2.26
KIF2C	kinesin family member 2C	0.003	1.76
KPNB1	karyopherin (importin) beta 1	< 0.001	1.63
KRT20	keratin 20	0.001	1.71
KRTAP26-1	keratin associated protein 26-1	0.003	-1.52

KYNU	kynureninase	0.002	-1.94
LASS3	ceramide synthase 3 (CERS3)	< 0.001	1.96
LDLR	low density lipoprotein receptor	< 0.001	3.13
LEPRE1	leucine proline-enriched proteoglycan (leprecan) 1	< 0.001	1.60
LMNB2	lamin B2	< 0.001	1.95
LOC100138671	uncharacterized LOC100138671	< 0.001	-1.59
LOC100138815	Ig lambda chain V-I region BL2-like	< 0.001	-1.77
LOC100139764	WW domain-binding protein 4-like	0.001	-1.54
LOC100139771	phosphatidic acid phosphatase type 2B-like	0.001	2.31
LOC100141258	interleukin-32-like	0.005	-1.53
LOC100295432	uncharacterized LOC100295432	0.003	-1.53
LOC100295767	uncharacterized LOC100295767	0.001	-1.70
LOC100297662	uncharacterized LOC100297662	0.003	-1.61
LOC100335357	uncharacterized LOC100335357	0.001	-1.69
LOC100335743	uncharacterized LOC100335743	0.001	1.57
LOC100336014	protease-like	0.004	1.63
LOC100336279	F-box protein 42 (FBXO42)	< 0.001	1.67
LOC100336628	mannosidase, alpha, class 1B, member 1-like	0.004	1.50
LOC100336753	mitochondrial import receptor subunit TOM6 homolog pseudogene	0.006	1.64
LOC509972	C-type lectin domain family 2 member D11-like	0.006	-3.23
LOC525863	histone cluster 1, H4i-like	0.001	1.73
LOC527083	Cytochrome P450 2G1-like	0.016	-1.53
LOC530348	chondroitin sulfate proteoglycan 4-like	0.005	-1.59
LOC540312	protein DDX26B-like	0.008	-1.54
LOC613739	pregnancy-associated glycoprotein 2-like	0.010	-2.25
LOC614423	phosphoglycerate mutase 1 (brain) pseudogene	< 0.001	1.73
LOC614669	PPPDE peptidase domain containing 2-like	< 0.001	1.67
LOC615247	aDAM metallopeptidase domain 20-like	0.001	-1.58
LOC617833	myelin and lymphocyte protein-like	0.011	3.56
LOC618012	histone H2B type 1-like	0.009	1.61
LOC784270	uncharacterized LOC784270	< 0.001	1.51
LOC785277	olfactory receptor, family 2, subfamily H, member 1-like	0.001	-1.74
LOC787709	X antigen family member 3-like	0.006	-1.62
LPCAT3	lysophosphatidylcholine acyltransferase 3	< 0.001	2.45

LRIG1	leucine-rich repeats and immunoglobulin-like domains 1	< 0.001	-1.57
LRRC36	leucine rich repeat containing 36	0.002	-1.53
LRRC39	leucine rich repeat containing 39	0.012	-1.51
LRRFIP1	leucine rich repeat (in FLII) interacting protein 1	< 0.001	-1.76
LSM11	LSM11, U7 small nuclear RNA associated	0.011	-1.50
LSM2	LSM2 homolog, U6 small nuclear RNA associated (S. cerevisiae)	< 0.001	1.58
LSMD1	LSM domain containing 1	< 0.001	1.70
LY6G5B	lymphocyte antigen 6 complex, locus G5B	0.001	-1.62
LYPLA1	lysophospholipase I	< 0.001	1.63
MACROD2	MACRO domain containing 2	0.001	-1.59
MAD2L1BP	MAD2L1 binding protein	0.001	1.54
MAGT1	magnesium transporter 1	< 0.001	1.54
MAN1B1	mannosidase, alpha, class 1B, member 1	0.001	1.54
MAP2K3	mitogen-activated protein kinase kinase 3	< 0.001	1.76
MCM5	minichromosome maintenance complex component 5	0.004	1.87
MCMDC2	minichromosome maintenance domain containing 2	0.001	-1.88
MDM4	Mdm4 p53 binding protein homolog (mouse)	0.001	-1.59
MEA1	male-enhanced antigen 1	< 0.001	1.77
MEIOB	meiosis specific with OB domains	0.006	-2.03
MESDC2	mesoderm development candidate 2	< 0.001	1.50
METTL13	methyltransferase like 13	< 0.001	1.71
MFGE8	milk fat globule-EGF factor 8 protein	0.001	1.56
MGC133950	spermatogenesis associated 6-like (SPATA6L)	0.001	-1.78
MGC137725	uncharacterized protein MGC137725	0.001	-1.52
MGC142695	RIKEN cDNA 2700097O09Rik-like	0.002	-1.52
MGC143209	family with sequence similarity 105, member A (FAM105A)	< 0.001	-2.89
MGC148938	protein phosphatase, Mg2+/Mn2+ dependent, 1N (putative) (PPM1N)	0.001	1.51
MGC152396	dynein, axonemal, heavy chain 3 (DNAH3)	0.005	-1.54
MGC152484	single-pass membrane protein with coiled-coil domains 1 (SMCO1)	0.002	-1.65
MGC157082	uncharacterized LOC788038	0.004	-2.23
MGC165715	chromosome 17 open reading frame, human C4orf29 (C17H4orf29)	0.003	-1.53
MIF	macrophage migration inhibitory factor (glycosylation-inhibiting factor)	0.006	1.61
MIF4GD	MIF4G domain containing	0.001	1.56
MIR218-1	microRNA mir-218-1	0.001	-1.70
MIR2329-1	microRNA mir-2329-1	0.002	1.90
MIR2340	microRNA mir-2340	0.013	-1.66
MIR2414	microRNA mir-2414	0.001	-1.56
MIR2431	microRNA mir-2431	0.012	-1.54
MIR2465	microRNA mir-2465	< 0.001	-1.79

MIR340	microRNA mir-340	< 0.001	-1.67
MIR877	microRNA mir-877	< 0.001	3.15
MIRLET7C	microRNA let-7c	0.010	-1.93
MIS18A	MIS18 kinetochore protein homolog A (S. pombe)	0.001	2.01
MLL	lysine (K)-specific methyltransferase 2A (KMT2A)	< 0.001	-1.51
MLLT11	myeloid/lymphoid or mixed-lineage leukemia (trithorax homolog, Drosophila); translocated to, 11	< 0.001	1.66
MMAB	methylmalonic aciduria (cobalamin deficiency) cblB type	< 0.001	1.71
MME	membrane metallo-endopeptidase	0.049	-1.63
MMS22L	MMS22-like, DNA repair protein	< 0.001	1.53
MON1A	MON1 homolog A (yeast)	< 0.001	1.79
MP68	chromosome 21 open reading frame (C21H14orf2)	0.001	1.52
MPND	MPN domain containing	0.005	1.51
MRI1	methylthioribose-1-phosphate isomerase homolog (S. cerevisiae)	< 0.001	1.53
MRPL4	mitochondrial ribosomal protein L4	< 0.001	1.54
MRPL48	mitochondrial ribosomal protein L48	0.001	1.80
MRPS11	mitochondrial ribosomal protein S11	< 0.001	1.60
MRPS28	mitochondrial ribosomal protein S28	< 0.001	1.62
MRTO4	mRNA turnover 4 homolog (S. cerevisiae)	< 0.001	1.73
MS4A7	membrane-spanning 4-domains, subfamily A, member 7	< 0.001	-1.69
MSH2	mutS homolog 2, colon cancer, nonpolyposis type 1 (E. coli)	0.007	1.59
MSR1	macrophage scavenger receptor 1	0.001	-1.68
MTX1	metaxin 1	0.001	1.56
MUC4	mucin 4, cell surface associated	0.030	-2.28
MVD	mevalonate (diphospho) decarboxylase	< 0.001	2.84
MYBBP1A	MYB binding protein (P160) 1a	0.001	1.50
N4BP2	NEDD4 binding protein 2	0.005	1.54
N4BP2L1	NEDD4 binding protein 2-like 1	0.004	-1.70
NAALADL2	N-acetylated alpha-linked acidic dipeptidase-like 2	< 0.001	-1.56
NAP1L5	nucleosome assembly protein 1-like 5	0.006	1.51
NCAPG2	non-SMC condensin II complex, subunit G2	0.003	1.63
NCKAP1L	NCK-associated protein 1-like	0.009	-1.90
NCLN	nicalin	< 0.001	1.67
NDRG2	NDRG family member 2	0.001	1.58
NDUFA7	NADH dehydrogenase (ubiquinone) 1 alpha subcomplex, 7	< 0.001	1.51
NDUFB11	NADH dehydrogenase (ubiquinone) 1 beta subcomplex, 11	< 0.001	1.52
NEU1	sialidase 1 (lysosomal sialidase)	< 0.001	1.56
NFIC	nuclear factor I/C (CCAAT-binding transcription factor)	< 0.001	1.96
NHS	Nance-Horan syndrome (congenital cataracts and dental anomalies)	< 0.001	-1.76
NKPD1	NTPase, KAP family P-loop domain containing 1	0.003	-1.60
NOB1	NIN1/RPN12 binding protein 1 homolog (S. cerevisiae)	0.001	1.62

NOL6	nucleolar protein family 6 (RNA-associated)	0.001	1.63
NOLC1	nucleolar and coiled-body phosphoprotein 1	0.007	1.50
NR1D1	nuclear receptor subfamily 1, group D, member 1	0.001	-1.63
NR1D2	nuclear receptor subfamily 1, group D, member 2	< 0.001	-1.64
NRF1	nuclear respiratory factor 1	< 0.001	-1.54
NRM	nurim (nuclear envelope membrane protein)	0.001	2.02
NUBP2	nucleotide binding protein 2	< 0.001	1.95
NUDT6	nudix (nucleoside diphosphate linked moiety X)-type motif 6	< 0.001	-1.67
OGFOD3	2-oxoglutarate and iron-dependent oxygenase domain containing 3	< 0.001	1.75
OIP5	Opa interacting protein 5	0.001	1.93
OSMR	oncostatin M receptor	0.016	-1.53
P2RY12	purinergic receptor P2Y, G-protein coupled, 12	0.001	-1.94
PABPC5	polyadenylate-binding protein 5-lik	0.023	1.91
PAG1	pregnancy-associated glycoprotein 1	0.001	-1.51
PARP8	poly (ADP-ribose) polymerase family, member 8	0.003	-1.67
PARPBP	PARP1 binding protein	0.003	2.15
PCDH20	protocadherin 20	0.022	-1.80
PCYT2	phosphate cytidylyltransferase 2, ethanolamine	< 0.001	2.46
PDCL3	phosducin-like 3	0.007	1.51
PDE4DIP	phosphodiesterase 4D interacting protein	< 0.001	1.51
PDGFC	platelet derived growth factor C	0.027	-1.52
PDIA3	protein disulfide isomerase family A, member 3	< 0.001	1.69
PER2	period homolog 2 (Drosophila)	0.007	-1.62
PFKL	phosphofructokinase, liver	0.001	1.63
PGAM1	phosphoglycerate mutase 1 (brain)	0.013	1.63
PGLS	6-phosphogluconolactonase	< 0.001	1.63
PGM1	phosphoglucomutase 1	0.001	1.56
PHACTR2	phosphatase and actin regulator 2	< 0.001	-1.65
PHGDH	phosphoglycerate dehydrogenase	0.011	2.52
PIK3R3	phosphoinositide-3-kinase, regulatory subunit 3 (gamma)	0.001	2.51
PION	pigeon homolog (Drosophila)	0.011	-1.55
PIP	prolactin-induced protein	0.024	1.76
PLA1A	phospholipase A1 member A	0.002	-1.63
PLEK2	pleckstrin 2	0.001	1.74
PLEKHA1	pleckstrin homology domain containing, family A (phosphoinositide binding specific) member 1	0.001	-1.51
PLXDC2	plexin domain containing 2	0.001	-1.53
PMM2	phosphomannomutase 2	< 0.001	1.72
PNPLA4	patatin-like phospholipase domain containing 4-like	0.002	-1.51
PNRC1	proline-rich nuclear receptor coactivator 1	< 0.001	-1.60
POLR2E	polymerase (RNA) II (DNA directed) polypeptide E, 25kDa	0.001	1.52

POLR2G	polymerase (RNA) II (DNA directed) polypeptide G	< 0.001	1.75
PPP1R1A	protein phosphatase 1, regulatory (inhibitor) subunit 1A	< 0.001	5.90
PRAF2	PRA1 domain family, member 2	< 0.001	1.96
PRDX2	peroxiredoxin 2	< 0.001	1.55
PREB	prolactin regulatory element binding	< 0.001	1.82
PRKACA	protein kinase, cAMP-dependent, catalytic, alpha	0.001	1.52
PRKAG3	protein kinase, AMP-activated, gamma 3 non-catalytic subunit	0.001	-1.64
PROSER2	proline and serine-rich protein 2	< 0.001	-1.50
PRRC1	proline-rich coiled-coil 1	< 0.001	1.64
PSMB6	proteasome (prosome, macropain) subunit, beta type, 6	< 0.001	1.70
PSRC1	proline/serine-rich coiled-coil 1	0.001	1.62
PTGES2	prostaglandin E synthase 2	0.003	1.55
PTHLH	parathyroid hormone-like hormone	0.003	1.55
PTN	pleiotrophin	0.036	1.95
PTPN13	protein tyrosine phosphatase, non-receptor type 13 (APO-1/CD95 (Fas)-associated phosphatase)	< 0.001	-1.66
PTPRT	protein tyrosine phosphatase, receptor type, T	0.017	1.66
PTPRU	protein tyrosine phosphatase, receptor type, U	< 0.001	-1.69
PURG	purine-rich element binding protein G	0.006	-1.65
PVALB	parvalbumin	0.002	2.80
QPRT	quinolinate phosphoribosyltransferase	< 0.001	1.62
RAB1A	RAB1A, member RAS oncogene family	< 0.001	1.53
RALGAPA2	Ral GTPase activating protein, alpha subunit 2 (catalytic)	0.002	-1.63
RAMP2	receptor (G protein-coupled) activity modifying protein 2	0.001	1.74
RAPGEFL1	Rap guanine nucleotide exchange factor (GEF)-like 1	0.019	1.51
RBBP7	retinoblastoma binding protein 7	< 0.001	1.54
RBMS2	RNA binding motif, single stranded interacting protein 2	< 0.001	-1.64
RECK	reversion-inducing-cysteine-rich protein with kazal motifs	0.006	-1.81
RFWD3	ring finger and WD repeat domain 3	0.001	1.61
RGS2	regulator of G-protein signaling 2, 24kDa	0.036	-1.54
RIC8B	resistance to inhibitors of cholinesterase 8 homolog B (C. elegans)	0.003	-1.58
RIPK4	receptor-interacting serine-threonine kinase 4	< 0.001	-1.58
RNASE6	ribonuclease, RNase A family, k6	0.001	-2.31
RNASEH2A	ribonuclease H2, subunit A	0.002	1.77
RNF149	ring finger protein 149	0.001	-2.03
RNF181	ring finger protein 181	< 0.001	1.52
RNPC3	RNA-binding region (RNP1, RRM) containing 3	0.002	-1.56
RORA	RAR-related orphan receptor A	< 0.001	-1.90
RP1	retinitis pigmentosa 1 (autosomal dominant)	0.002	-1.53
RPL38	ribosomal protein L38	< 0.001	1.71
RPL7A	ribosomal protein L7a	< 0.001	1.91

RPL8	ribosomal protein L8	0.003	1.52
RRAGB	Ras-related GTP binding B	< 0.001	-2.00
RRM1	ribonucleotide reductase M1	0.002	1.58
RRP9	ribosomal RNA processing 9, small subunit (SSU) processome component, homolog (yeast)	0.001	1.57
SAA3	serum amyloid A 3	0.020	-2.72
SAMSN1	SAM domain, SH3 domain and nuclear localization signals 1	0.004	-1.95
SC4MOL	methylsterol monooxygenase 1 (MSMO1)	< 0.001	2.58
SCAND1	SCAN domain containing 1	< 0.001	1.56
SCNN1G	sodium channel, nonvoltage-gated 1, gamma	0.001	-2.57
SDR16C5	short chain dehydrogenase/reductase family 16C, member 5	< 0.001	-1.62
SEC13	SEC13 homolog (S. cerevisiae)	< 0.001	1.50
SEC14L3	SEC14-like 3 (S. cerevisiae)	0.025	-1.95
SEC23B	Sec23 homolog B (S. cerevisiae)	< 0.001	1.57
SEC61A1	Sec61 alpha 1 subunit (S. cerevisiae)	< 0.001	1.93
SELENBP1	selenium binding protein 1	0.004	-1.83
SELPLG	selectin P ligand	0.008	-1.57
SEPP1	selenoprotein P, plasma, 1	< 0.001	-1.78
SEPX1	selenoprotein X, 1	< 0.001	1.61
SERPING1	serpin peptidase inhibitor, clade G (C1 inhibitor), member 1	0.021	-2.82
SGCD	sarcoglycan, delta (35kDa dystrophin-associated glycoprotein)	0.004	2.13
SH3BGRL3	SH3 domain binding glutamic acid-rich protein like 3	0.002	1.67
SHC1	SHC (Src homology 2 domain containing) transforming protein 1	< 0.001	1.59
SHC4	SHC (Src homology 2 domain containing) family, member 4	0.006	-1.67
SHKBP1	SH3KBP1 binding protein 1	0.005	1.51
SIVA1	SIVA1, apoptosis-inducing factor	0.002	1.58
SKA3	spindle and kinetochore associated complex subunit 3	0.001	1.88
SLC12A8	solute carrier family 12 (potassium/chloride transporters), member 8	< 0.001	2.06
SLC13A5	solute carrier family 13 (sodium-dependent citrate transporter), member 5	0.003	2.96
SLC16A1	solute carrier family 16, member 1 (monocarboxylic acid transporter 1)	0.004	2.24
SLC18B1	solute carrier family 18, subfamily B, member 1	< 0.001	-1.64
SLC25A22	solute carrier family 25 (mitochondrial carrier: glutamate), member 22	0.001	1.51
SLC25A25	solute carrier family 25 (mitochondrial carrier; phosphate carrier), member 25	< 0.001	1.66
SLC25A39	solute carrier family 25, member 39	0.001	1.64
SLC25A6	solute carrier family 25 (mitochondrial carrier; adenine nucleotide translocator), member 6	0.007	1.59
SLC27A4	solute carrier family 27 (fatty acid transporter), member 4	0.001	1.72
SLC2A8	solute carrier family 2 (facilitated glucose transporter), member 8	< 0.001	1.59
SLC34A2	solute carrier family 34 (sodium phosphate), member 2	0.002	2.01

SLC35D2	solute carrier family 35, member D2	0.001	1.54
SLC38A1	solute carrier family 38, member 1	< 0.001	1.89
SLC39A12	solute carrier family 39 (zinc transporter), member 12	< 0.001	1.68
SLC39A7	solute carrier family 39 (zinc transporter), member 7	< 0.001	1.84
SLC41A1	solute carrier family 41, member 1	0.001	1.53
SLC45A2	solute carrier family 45, member 2	< 0.001	4.68
SLC46A2	solute carrier family 46, member 2	0.002	1.89
SLC5A1	solute carrier family 5 (sodium/glucose cotransporter), member 1	0.004	-1.99
SLC5A3	solute carrier family 5 (sodium/myo-inositol cotransporter), member 3	< 0.001	-1.74
SLC5A6	solute carrier family 5 (sodium-dependent vitamin transporter), member 6	0.001	1.89
SLC7A1	solute carrier family 7 (cationic amino acid transporter, y+ system), member 1	< 0.001	1.58
SLCO3A1	solute carrier organic anion transporter family, member 3A1	0.015	-1.86
SMARCB1	SWI/SNF related, matrix associated, actin dependent regulator of chromatin, subfamily b, member 1	< 0.001	1.60
SMO	smoothened, frizzled family receptor	0.001	-1.53
SND1	staphylococcal nuclease and tudor domain containing 1	< 0.001	1.50
SOCS4	suppressor of cytokine signaling 4	< 0.001	1.62
SPATA7	spermatogenesis associated 7	0.001	-1.64
SPC24	NDC80 kinetochore complex component, homolog (S. cerevisiae)	0.003	1.90
SPDEF	SAM pointed domain containing ets transcription factor	0.004	1.71
SPIN4	spindlin family, member 4	0.022	1.60
SRCIN1	SRC kinase signaling inhibitor	0.001	-1.67
SREBF1	sterol regulatory element binding transcription factor 1	< 0.001	1.59
SREBF2	sterol regulatory element binding transcription factor 2	< 0.001	1.57
SRF	serum response factor (c-fos serum response element-binding transcription factor)	< 0.001	1.61
SRSF5	serine/arginine-rich splicing factor 5	< 0.001	-1.84
SSLP1	secreted seminal-vesicle Ly-6 protein 1	0.008	-2.18
SSTR1	540013somatostatin receptor 1	0.002	2.07
STAP2	signal transducing adaptor family member 2	0.002	1.91
STK16	serine/threonine kinase 16	< 0.001	1.71
STOML2	stomatin (EPB72)-like 2	< 0.001	1.58
STX5	syntaxin 5	< 0.001	1.63
SULT1C4	sulfotransferase family, cytosolic, 1C, member 4	0.004	-1.72
SYNE1	spectrin repeat containing, nuclear envelope 1	0.002	-1.58
SYNE2	spectrin repeat containing, nuclear envelope 2	< 0.001	-1.62
SYNGR1	synaptogyrin 1	< 0.001	1.79
TAF1A	TATA box binding protein (TBP)-associated factor, RNA polymerase I, A, 48kDa	0.001	-1.58
TARBP2	TAR (HIV-1) RNA binding protein 2	< 0.001	1.50
TBC1D1	TBC1 (tre-2/USP6, BUB2, cdc16) domain family, member 1	< 0.001	1.62

TBC1D2B	TBC1 domain family, member 2B	0.002	-1.64
TBCB	tubulin folding cofactor B	< 0.001	1.51
TCAM1	testicular cell adhesion molecule 1	0.002	-1.58
TCEAL8	transcription elongation factor A (SII)-like 8	< 0.001	1.51
TCP11	t-complex 11 homolog (mouse)	0.001	-1.77
TECR	trans-2,3-enoyl-CoA reductase	< 0.001	1.53
TENC1	tensin like C1 domain containing phosphatase (tensin 2)	0.001	-1.56
TFG	TRK-fused gene	< 0.001	1.55
TGFBR2	transforming growth factor, beta receptor II (70/80kDa)	0.002	-1.52
TGM2	transglutaminase 2 (C polypeptide, protein-glutamine-gamma-glutamyltransferase)	0.007	-2.18
TIMM8B	translocase of inner mitochondrial membrane 8 homolog B (yeast)	< 0.001	1.57
TIMP3	TIMP metallopeptidase inhibitor 3	0.017	-1.83
TLR7	toll-like receptor 7	0.002	-1.75
TM7SF2	transmembrane 7 superfamily member 2	< 0.001	2.16
TMBIM1B	transmembrane Bax inhibitor motif containing 1B	0.006	1.57
TMEM106A	transmembrane protein 106A	0.012	-1.54
TMEM116	transmembrane protein 116	0.004	-1.80
TMEM125	transmembrane protein 125	< 0.001	1.60
TMEM165	transmembrane protein 165	< 0.001	1.71
TMEM171	transmembrane protein 171	0.008	-2.14
TMEM216	transmembrane protein 216	0.009	-1.52
TMEM217	transmembrane protein 217	0.001	3.25
TMUB1	transmembrane and ubiquitin-like domain containing 1	< 0.001	1.55
TNFRSF10A	tumor necrosis factor receptor superfamily, member 10a	0.005	1.64
TOMM40	translocase of outer mitochondrial membrane 40 homolog (yeast)	< 0.001	1.69
TOR1A	torsin family 1, member A (torsin A)	0.001	1.63
TPM1	tropomyosin 1 (alpha)	0.004	-1.71
TRAF5	TNF receptor-associated factor 5	0.014	-1.70
TRAPPC2	trafficking protein particle complex 2	0.001	1.51
TRAT1	T cell receptor associated transmembrane adaptor 1	0.007	-3.48
TRIB2	tribbles homolog 2 (Drosophila)	0.008	1.79
TRIM32	tripartite motif containing 32	0.001	1.59
TRIOBP	TRIO and F-actin binding protein	0.002	1.52
TRMT6	tRNA methyltransferase 6 homolog (S. cerevisiae)	< 0.001	1.53
TSPAN13	tetraspanin 13	0.001	1.57
TSPAN17	tetraspanin 17	0.003	1.78
TUBB6	tubulin, beta 6 class V	< 0.001	1.59
TUBD1	tubulin, delta 1	< 0.001	-1.94
TXNIP	thioredoxin interacting protein	0.001	-1.53
TYROBP	TYRO protein tyrosine kinase binding protein	0.001	-1.59

UBA5	ubiquitin-like modifier activating enzyme 5	0.001	1.59
UBE2J1	ubiquitin-conjugating enzyme E2, J1, U	< 0.001	2.05
UBE2M	ubiquitin-conjugating enzyme E2M	< 0.001	1.60
UCK2	uridine-cytidine kinase 2	0.007	1.51
UNC13B	unc-13 homolog B (C. elegans)	0.004	1.88
UNC13C	unc-13 homolog C (C. elegans)	0.006	4.07
UNC13D	unc-13 homolog D (C. elegans)	0.027	1.55
UPP1	uridine phosphorylase 1	< 0.001	2.09
URM1	ubiquitin related modifier 1	< 0.001	1.62
USE1	unconventional SNARE in the ER 1 homolog (S. cerevisiae)	0.001	1.52
USP5	ubiquitin specific peptidase 5 (isopeptidase T)	< 0.001	1.74
VAMP4	vesicle-associated membrane protein 4	< 0.001	-1.51
VARS	valyl-tRNA synthetase	< 0.001	1.78
VAV3	vav 3 guanine nucleotide exchange factor	< 0.001	-2.56
VCP	valosin containing protein	< 0.001	1.58
VIM	vimentin	0.022	-1.58
VRK1	vaccinia related kinase 1	0.005	1.53
WDR89	WD repeat domain 89	0.012	1.58
WDR96	WD repeat domain 96	0.001	-1.67
WIPI1	WD repeat domain, phosphoinositide interacting 1	< 0.001	1.54
YIF1A	Yip1 interacting factor homolog A (S. cerevisiae)	< 0.001	1.63
YIF1B	Yip1 interacting factor homolog B (S. cerevisiae)	< 0.001	1.54
YRDC	yrdC domain containing (E. coli)	< 0.001	1.52
ZC3H6	zinc finger CCCH-type containing 6	< 0.001	-1.70
ZCRB1	zinc finger CCHC-type and RNA binding motif 1	0.012	1.54
ZFP36L1	zinc finger protein 36, C3H type-like 1	< 0.001	-1.77
ZKSCAN5	zinc finger with KRAB and SCAN domains 5	< 0.001	-1.57
ZNF274	zinc finger protein 274	0.001	-1.59
ZNF292	zinc finger protein 292	< 0.001	-1.58
ZNF502	zinc finger protein 502	< 0.001	-1.52
ZNF593	zinc finger protein 593	< 0.001	1.52
ZNF605	zinc finger protein 605	< 0.001	-1.53
ZNF704	zinc finger protein 704	< 0.001	-1.52
ZNF772	zinc finger protein 17 (ZNF17)	0.004	-1.56
ZNF804B	zinc finger protein 804B	< 0.001	3.57
ZNF850	zinc finger protein 850	0.012	-1.57
ZNF862	zinc finger protein 862	< 0.001	-1.60
**FP vs. LP (Isthmus)**			
**Gene Symbol**	**Gene Description**	**p-value**	**Fold-Change**
MARCH1	membrane-associated ring finger (C3HC4) 1	0.004	-1.86
MARCH2	membrane-associated ring finger (C3HC4) 2	0.004	1.55

MARCH8	membrane-associated ring finger (C3HC4) 8	< 0.001	-1.51
A2ML1	alpha-2-macroglobulin-like 1	0.046	-1.63
ABAT	4-aminobutyrate aminotransferase	< 0.001	-1.61
ABCA5	ATP-binding cassette, sub-family A (ABC1), member 5	< 0.001	-1.60
ABCC1	ATP-binding cassette, sub-family C (CFTR/MRP), member 1	0.008	-1.89
ABCE1	ATP-binding cassette, sub-family E (OABP), member 1	< 0.001	1.53
ABCG2	ATP-binding cassette, sub-family G (WHITE), member 2	0.012	1.73
ABHD2	abhydrolase domain containing 2	0.001	-1.52
ABLIM2	actin binding LIM protein family, member 2	< 0.001	-2.31
ACER2	alkaline ceramidase 2	0.001	-1.51
ACMSD	aminocarboxymuconate semialdehyde decarboxylase	0.004	-1.56
ACOT13	acyl-CoA thioesterase 13	< 0.001	1.55
ACOX3	acyl-CoA oxidase 3, pristanoyl	< 0.001	-2.75
ACP1	acid phosphatase 1, soluble	0.006	1.53
ACP5	acid phosphatase 5, tartrate resistant	0.002	-1.56
ACSF2	acyl-CoA synthetase family member 2	0.005	-1.56
ACSL1	acyl-CoA synthetase long-chain family member 1	< 0.001	-1.53
ACSS1	acyl-CoA synthetase short-chain family member 1	0.004	-1.51
ACVR1C	activin A receptor, type IC	0.005	-2.30
ADAMTS16	ADAM metallopeptidase with thrombospondin type 1 motif, 16	0.005	-1.72
ADAMTS18	ADAM metallopeptidase with thrombospondin type 1 motif, 18	0.018	-1.67
ADAMTS3	ADAM metallopeptidase with thrombospondin type 1 motif, 3	0.007	2.95
ADAMTS5	ADAM metallopeptidase with thrombospondin type 1 motif, 5	0.001	3.79
ADAP2	ArfGAP with dual PH domains 2	0.010	-1.57
ADCK1	aarF domain containing kinase 1	0.002	-1.77
ADCY6	adenylate cyclase 6	< 0.001	-1.53
ADCY9	adenylate cyclase 9	< 0.001	-1.51
ADK	adenosine kinase	< 0.001	1.76
AFAP1	actin filament associated protein 1	< 0.001	-1.71
AGPAT4	1-acylglycerol-3-phosphate O-acyltransferase 4 (lysophosphatidic acid acyltransferase, delta)	0.001	2.07
AK5	adenylate kinase 5	< 0.001	-4.74
AKAP5	A kinase (PRKA) anchor protein 5	< 0.001	-3.68
AKAP8	A kinase (PRKA) anchor protein 8	< 0.001	-1.65
AKR1B1	aldo-keto reductase family 1, member B1 (aldose reductase)	< 0.001	-2.65
AKTIP	AKT interacting protein	< 0.001	1.61
ALAD	aminolevulinate dehydratase	0.003	-1.58
ALDH1A1	aldehyde dehydrogenase 1 family, member A1	0.001	2.33
ALDH6A1	aldehyde dehydrogenase 6 family, member A1	0.001	-1.62
ALG14	asparagine-linked glycosylation 14 homolog (S. cerevisiae)	0.006	1.51
AMD1	adenosylmethionine decarboxylase 1	< 0.001	1.85

AMIGO1	adhesion molecule with Ig-like domain 1	< 0.001	-2.28
ANKS1B	ankyrin repeat and sterile alpha motif domain containing 1B	0.001	2.43
ANKS4B	ankyrin repeat and sterile alpha motif domain containing 4B	0.014	1.93
ANKUB1	ankyrin repeat and ubiquitin domain containing 1	< 0.001	-2.38
ANO1	anoctamin 1, calcium activated chloride channel	0.001	4.45
ANO3	anoctamin 3	0.004	2.49
ANTXR2	anthrax toxin receptor 2	0.015	-1.60
ANXA1	annexin A1	0.001	1.54
APBA1	amyloid beta (A4) precursor protein-binding, family A, member 1	0.011	-1.73
APC	adenomatous polyposis coli	< 0.001	-1.60
APMAP	adipocyte plasma membrane associated protein	0.001	-1.66
APOBEC3B	apolipoprotein B mRNA editing enzyme, catalytic polypeptide-like 3B	< 0.001	-1.71
AQP4	aquaporin 4	< 0.001	-1.79
AQP5	aquaporin 5	0.002	1.57
AR	androgen receptor	0.001	1.63
ARHGAP31	Rho GTPase activating protein 31	0.001	-1.64
ARHGAP44	Rho GTPase activating protein 44	0.048	-2.60
ARHGEF2	Rho/Rac guanine nucleotide exchange factor (GEF) 2	< 0.001	-1.62
ARHGEF26	Rho guanine nucleotide exchange factor (GEF) 26	< 0.001	-2.76
ARL4A	ADP-ribosylation factor-like 4A	0.004	-1.61
ARSA	arylsulfatase A	< 0.001	-1.76
ASGR2	asialoglycoprotein receptor 2	0.026	1.67
ATAD2	ATPase family, AAA domain containing 2	< 0.001	1.75
ATF7	activating transcription factor 7	< 0.001	-1.60
ATG2B	autophagy related 2B	< 0.001	-1.58
ATP2A3	ATPase, Ca++ transporting, ubiquitous	0.001	-2.40
ATP2B1	ATPase, Ca++ transporting, plasma membrane 1	0.001	1.81
ATP2B2	ATPase, Ca++ transporting, plasma membrane 2	< 0.001	2.58
ATP2C2	ATPase, Ca++ transporting, type 2C, member 2	0.004	2.39
ATP5E	ATP synthase, H+ transporting, mitochondrial F1 complex, epsilon subunit	< 0.001	1.52
ATRNL1	attractin-like 1	0.026	3.52
ATXN3	ataxin 3	< 0.001	-1.59
ATXN7L1	ataxin 7-like 1	< 0.001	-1.53
AUTS2	autism susceptibility candidate 2	< 0.001	-2.11
B3GALT2	UDP-Gal:betaGlcNAc beta 1,3-galactosyltransferase, polypeptide 2	0.023	-2.06
B4GALNT1	beta-1,4-N-acetyl-galactosaminyl transferase 1	< 0.001	1.92
B4GALT4	UDP-Gal:betaGlcNAc beta 1,4- galactosyltransferase, polypeptide 4	0.003	1.64
B4GALT6	UDP-Gal:betaGlcNAc beta 1,4- galactosyltransferase, polypeptide 6	0.008	-2.13

BAMBI	BMP and activin membrane-bound inhibitor homolog (Xenopus laevis)	< 0.001	-2.79
BCAS3	breast carcinoma amplified sequence 3	< 0.001	-1.50
BCL11A	B-cell CLL/lymphoma 11A (zinc finger protein)	0.004	1.61
BCL2L15	BCL2-like 15	0.009	-2.26
BCL7A	B-cell CLL/lymphoma 7A	< 0.001	-1.77
BEND4	BEN domain containing 4	0.047	1.59
BEND7	BEN domain containing 7	< 0.001	-1.51
BET3L	BET3 like (S. cerevisiae)	0.003	5.27
BLOC1S3	biogenesis of lysosomal organelles complex-1, subunit 3	0.015	-1.87
BMP2	bone morphogenetic protein 2	< 0.001	-1.63
BNC2	basonuclin 2	0.010	3.60
BNIP3	BCL2/adenovirus E1B 19kDa interacting protein 3	< 0.001	-1.59
BNIPL	BCL2/adenovirus E1B 19kD interacting protein like	0.002	3.42
BoLA	MHC class I heavy chain	0.002	-1.85
BOLA3	bolA homolog 3 (E. coli)	< 0.001	1.71
BOSTAUV1R416	vomeronasal 1 receptor bosTauV1R416	0.010	-2.10
BRAP	BRCA1 associated protein	< 0.001	-1.97
BREH1	retinyl ester hydrolase type 1	0.004	2.90
BSC1	solute carrier family 12 (sodium/potassium/chloride transporters), member 1 (SLC12A1)	0.046	2.01
BSP3	binder of sperm 3	< 0.001	-7.58
BTG3	BTG family, member 3	0.001	1.71
C10H5orf13	chromosome 10 open reading frame	0.027	-1.75
C11H2ORF7	protease-associated domain containing 1 (PRADC1)	0.004	1.76
C12H13orf27	testis expressed 30 (TEX30)	0.005	1.65
C16H1orf115	chromosome 16 open reading frame	0.009	-1.58
C1H21ORF91	chromosome 1 open reading frame	< 0.001	1.61
C1QTNF5	C1q and tumor necrosis factor related protein 5	0.046	1.64
C22H3ORF64	chromosome 22 open reading frame (EOGT)	0.003	-1.66
C27H8orf48	chromosome 27 open reading frame	0.002	1.79
C27H8ORF79	putative methyltransferase KIAA1456 homolog (KIAA1456)	< 0.001	-1.77
C28H10orf99	chromosome 28 open reading frame	0.015	-1.63
C3H1orf189	chromosome 3 open reading frame	0.012	1.59
C3H1orf216	chromosome 3 open reading frame	0.003	1.50
C5	complement component 5	0.004	-2.20
C5H12orf35	chromosome 5 open reading frame	< 0.001	1.50
C5orf28	chromosome 20 open reading frame (C20H5orf28)	0.002	-1.52

C6H4orf48	chromosome 6 open reading frame	0.004	1.61
C8H9orf21	AhpC/TSA antioxidant enzyme domain containing 1 (AAED1)	< 0.001	1.94
CA10	carbonic anhydrase X	0.001	-4.40
CA11	carbonic anhydrase XI	0.004	1.63
CA5B	carbonic anhydrase VB, mitochondrial	< 0.001	-2.10
CABLES1	Cdk5 and Abl enzyme substrate 1	0.006	-1.57
CAMK2N1	calcium/calmodulin-dependent protein kinase II inhibitor 1	0.001	-2.51
CAMTA1	calmodulin binding transcription activator 1	0.005	1.52
CAPN2	calpain 2, (m/II) large subunit	0.002	1.60
CBR1	carbonyl reductase 1	< 0.001	-1.58
CCDC101	coiled-coil domain containing 101	< 0.001	-1.60
CCDC68	coiled-coil domain containing 68	0.019	-1.85
CCDC8	coiled-coil domain containing 8	0.009	1.54
CCL26	chemokine (C-C motif) ligand 26	0.043	2.86
CCNA1	cyclin A1	0.001	1.78
CCND2	cyclin D2	0.003	2.36
CCND3	cyclin D3	< 0.001	-2.11
CCNG2	cyclin G2	< 0.001	-1.65
CCT7	chaperonin containing TCP1, subunit 7 (eta)	< 0.001	1.58
CDC34	cell division cycle 34 homolog (S. cerevisiae)	< 0.001	-1.52
CDC42BPA	CDC42 binding protein kinase alpha (DMPK-like)	< 0.001	1.55
CDH22	cadherin 22, type 2	0.001	2.31
CDH6	cadherin 6, type 2, K-cadherin (fetal kidney)	0.024	1.86
CDH7	cadherin 7, type 2	0.020	-1.60
CDK14	cyclin-dependent kinase 14	0.038	1.51
CDK7	cyclin-dependent kinase 7	0.001	-1.54
CDKN1A	cyclin-dependent kinase inhibitor 1A (p21, Cip1)	0.010	1.56
CDKN1C	cyclin-dependent kinase inhibitor 1C (p57, Kip2)	0.048	-1.56
CDO1	cysteine dioxygenase, type I	< 0.001	2.32
CENPF	centromere protein F, 350/400kDa (mitosin)	0.037	1.83
CFH	complement factor H	0.003	1.92
CHDH	choline dehydrogenase	0.008	1.52
CHI3L1	chitinase 3-like 1 (cartilage glycoprotein-39)	0.001	7.34
CHIC1	cysteine-rich hydrophobic domain 1	0.001	-1.58
CHRNA5	cholinergic receptor, nicotinic, alpha 5	0.009	1.59
CHRNA6	cholinergic receptor, nicotinic, alpha 6	0.004	1.69
CISH	cytokine inducible SH2-containing protein	< 0.001	1.55
CKAP2L	cytoskeleton associated protein 2-like	0.005	1.52
CLCN5	chloride channel 5	0.003	2.00
CLCN6	chloride channel 6	< 0.001	-1.58
CLCN7	chloride channel 7	< 0.001	-1.60

CLDN3	claudin 3	< 0.001	-1.76
CLDN4	claudin 4	< 0.001	-2.61
CLEC3A	C-type lectin domain family 3, member A	< 0.001	8.69
CLEC4E	C-type lectin domain family 4, member E	0.041	1.96
CLIC2	chloride intracellular channel 2	0.003	2.06
CLIP4	CAP-GLY domain containing linker protein family, member 4	0.003	-3.10
CMAS	cytidine monophosphate N-acetylneuraminic acid synthetase	0.001	1.55
CMPK2	cytidine monophosphate (UMP-CMP) kinase 2, mitochondrial	0.005	-1.69
CMTM3	CKLF-like MARVEL transmembrane domain containing 3	0.006	1.57
CMTM8	CKLF-like MARVEL transmembrane domain containing 8	< 0.001	1.72
COBL	cordon-bleu homolog (mouse)	0.002	-1.65
COL15A1	collagen, type XV, alpha 1	0.001	2.50
COL25A1	collagen, type XXV, alpha 1	< 0.001	-1.61
COL6A6	collagen, type VI, alpha 6	< 0.001	-2.61
COX11	COX11 cytochrome c oxidase assembly homolog (yeast)	0.001	1.54
COX6A1	cytochrome c oxidase subunit VIa polypeptide 1	0.009	1.51
COX6B1	cytochrome c oxidase subunit VIb polypeptide 1 (ubiquitous)	0.002	1.69
COX6C	cytochrome c oxidase subunit VIc	< 0.001	1.56
CPE	carboxypeptidase E	0.048	-1.84
CPEB3	cytoplasmic polyadenylation element binding protein 3	< 0.001	-1.62
CPN1	carboxypeptidase N, polypeptide 1	0.002	-3.08
CPT1B	carnitine palmitoyltransferase 1B (muscle)	0.004	1.55
CRLF3	cytokine receptor-like factor 3	< 0.001	-1.65
CRTAC1	cartilage acidic protein 1	< 0.001	1.71
CRTC1	CREB regulated transcription coactivator 1	< 0.001	-2.06
CRTC3	CREB regulated transcription coactivator 3	0.001	-1.52
CRYAB	crystallin, alpha B	< 0.001	2.30
CTNNAL1	catenin (cadherin-associated protein), alpha-like 1	< 0.001	-1.93
CTSL2	cathepsin L2	< 0.001	1.81
CTTN	cortactin	0.001	1.54
CYB5R1	cytochrome b5 reductase 1	< 0.001	2.20
CYBRD1	cytochrome b reductase 1	0.004	-1.91
CYLC1	cylicin, basic protein of sperm head cytoskeleton 1	< 0.001	-4.03
CYP1B1	cytochrome P450, family 1, subfamily B, polypeptide 1	0.023	1.98
CYP2S1	cytochrome P450, family 2, subfamily S, polypeptide 1	< 0.001	2.17
CYYR1	cysteine/tyrosine-rich 1	0.009	-1.63
DAAM2	dishevelled associated activator of morphogenesis 2	0.009	-2.13
DAB2IP	DAB2 interacting protein	0.001	1.64
DCLK1	doublecortin-like kinase 1	0.001	4.08
DDO	D-aspartate oxidase	< 0.001	-1.93
DEGS1	degenerative spermatocyte homolog 1, lipid desaturase	< 0.001	1.61

	(Drosophila)		
DENND2C	DENN/MADD domain containing 2C	0.018	1.60
DENND3	DENN/MADD domain containing 3	0.001	-1.53
DEPDC6	DEP domain containing MTOR-interacting protein (DEPTOR)	0.001	-1.69
DHRS3	dehydrogenase/reductase (SDR family) member 3	0.020	-1.60
DHRS7	dehydrogenase/reductase (SDR family) member 7	0.018	1.97
DIMT1L	DIM1 dimethyladenosine transferase 1 homolog (S. cerevisiae)	< 0.001	1.57
DIO2	deiodinase, iodothyronine, type II	< 0.001	1.64
DLC1	deleted in liver cancer 1	0.010	-1.80
DLGAP1	discs, large (Drosophila) homolog-associated protein 1	0.001	-1.74
DLL4	delta-like 4 (Drosophila)	0.001	-2.05
DMRT2	doublesex and mab-3 related transcription factor 2	0.002	2.09
DNAJC19	DnaJ (Hsp40) homolog, subfamily C, member 19	< 0.001	1.64
DNASE1L1	deoxyribonuclease I-like 1	< 0.001	1.60
DNMT3A	DNA (cytosine-5-)-methyltransferase 3 alpha	< 0.001	-1.51
DPP10	dipeptidyl-peptidase 10 (non-functional)	0.001	-3.75
DSG3	desmoglein 3	< 0.001	-1.96
DTX3L	deltex 3-like (Drosophila)	0.004	-1.58
DTX4	deltex homolog 4 (Drosophila)	< 0.001	-1.76
DUSP6	dual specificity phosphatase 6	0.004	-1.63
DYNLT1	dynein, light chain, Tctex-type 1	< 0.001	1.52
EBPL	emopamil binding protein-like	< 0.001	1.95
ECE2	endothelin converting enzyme 2	0.001	1.59
EEF1B2	eukaryotic translation elongation factor 1 beta 2	< 0.001	1.57
EFHD1	EF-hand domain family, member D1	0.004	-1.62
EFNA4	ephrin-A4	< 0.001	-1.77
EFNA5	ephrin-A5	0.033	1.61
EGFLAM	EGF-like, fibronectin type III and laminin G domains	0.033	1.66
EHD3	EH-domain containing 3	0.010	2.10
ELF5	E74-like factor 5 (ets domain transcription factor)	0.048	1.83
ENPP4	ectonucleotide pyrophosphatase/phosphodiesterase 4 (putative)	< 0.001	-1.87
ERAP2	endoplasmic reticulum aminopeptidase 2	0.007	-1.51
ERGIC2	ERGIC and golgi 2	< 0.001	1.58
ERRFI1	ERBB receptor feedback inhibitor 1	0.001	-1.67
ESRRG	estrogen-related receptor gamma	0.012	1.89
ETV1	ets variant 1	0.002	-2.47
EXPH5	exophilin 5	0.004	-1.50
EXTL3	exostoses (multiple)-like 3	< 0.001	1.85
FAIM	Fas apoptotic inhibitory molecule	0.007	-1.53
FAM102B	family with sequence similarity 102, member B	0.015	1.96
FAM124B	family with sequence similarity 124B	0.002	1.89

FAM125B	multivesicular body subunit 12B (MVB12B)	0.004	-1.53
FAM20C	family with sequence similarity 20, member C	0.001	-1.51
FAM55C	neurexophilin and PC-esterase domain family, member 3 (NXPE3)	0.001	-1.73
FAM59A	GRB2 associated, regulator of MAPK1 (GAREM)	< 0.001	-2.04
FBLN1	fibulin 1	0.003	-2.07
FCGRT	Fc fragment of IgG, receptor, transporter, alpha	0.006	1.59
FERMT1	fermitin family member 1	< 0.001	-1.91
FGF13	fibroblast growth factor 13	0.016	-1.51
FGF14	fibroblast growth factor 14	0.002	-1.97
FGFRL1	fibroblast growth factor receptor-like 1	0.007	-1.56
FJX1	four jointed box 1 (Drosophila)	0.002	2.00
FKBP15	FK506 binding protein 15, 133kDa	< 0.001	-1.59
FKBP4	FK506 binding protein 4, 59kDa	0.001	1.52
FOSL2	FOS-like antigen 2	0.001	1.77
FOXP1	forkhead box P1	< 0.001	-1.55
FXYD6	FXYD domain containing ion transport regulator 6	0.001	2.41
FZD10	frizzled family receptor 10	0.002	-1.77
FZD5	frizzled family receptor 5	0.014	-1.77
G6PD	glucose-6-phosphate dehydrogenase	< 0.001	1.54
GABBR2	gamma-aminobutyric acid (GABA) B receptor, 2	0.003	2.36
GAL3ST2	galactose-3-O-sulfotransferase 2	< 0.001	-3.02
GAT	glycine-N-acyltransferase-like	0.005	-2.18
GATA5	GATA binding protein 5	< 0.001	-1.53
GATSL2	GATS protein-like 2	0.005	-1.53
GDF10	growth differentiation factor 10	0.002	-1.59
GGT1	gamma-glutamyltransferase 1	0.001	-1.51
GJB5	gap junction protein, beta 5, 31.1kDa	0.002	-1.89
GLIS3	GLIS family zinc finger 3	< 0.001	-1.79
GM2A	GM2 ganglioside activator	0.012	-1.67
GNAI1	guanine nucleotide binding protein (G protein), alpha inhibiting activity polypeptide 1	0.006	-1.85
GNAL	guanine nucleotide binding protein (G protein), alpha activating activity polypeptide, olfactory type	< 0.001	-2.73
GNAO1	guanine nucleotide binding protein (G protein), alpha activating activity polypeptide O	< 0.001	-1.97
GNL3	guanine nucleotide binding protein-like 3 (nucleolar)	0.002	1.52
GPC6	glypican 6	0.017	-1.66
GPR162	G protein-coupled receptor 162	0.020	1.53
GRIP1	glutamate receptor interacting protein 1	< 0.001	2.23
GSN	gelsolin	0.002	-1.70
GSTA1	glutathione S-transferase alpha 2#N/A	0.002	-3.86
GSTT3	glutathione S-transferase, theta 3	0.007	-1.79

GTF3C4	general transcription factor IIIC, polypeptide 4, 90kDa	< 0.001	-1.51
GUCY1A3	guanylate cyclase 1, soluble, alpha 3	0.011	-1.55
GUCY2C	guanylate cyclase 2C (heat stable enterotoxin receptor)	0.011	-1.55
GULP1	GULP, engulfment adaptor PTB domain containing 1	< 0.001	-2.52
GUSB	glucuronidase, beta	< 0.001	1.82
GYLTL1B	glycosyltransferase-like 1B	0.001	-1.70
GYPC	glycophorin C (Gerbich blood group)	0.013	2.22
H2AFY2	H2A histone family, member Y2	0.003	1.62
HAS3	hyaluronan synthase 3	0.003	-1.80
HDHD2	haloacid dehalogenase-like hydrolase domain containing 2	< 0.001	1.57
HECW2	HECT, C2 and WW domain containing E3 ubiquitin protein ligase 2	0.001	2.25
HEG1	HEG homolog 1 (zebrafish)	0.009	-1.53
HEPHL1	hephaestin-like 1	0.002	-2.58
HEY1	hairy/enhancer-of-split related with YRPW motif 1	0.005	-2.16
HEY2	hairy/enhancer-of-split related with YRPW motif 2	0.005	2.27
HHIPL2	HHIP-like 2	0.001	-1.57
HIGD1D	HIG1 domain family, member 1D	< 0.001	1.54
HNF1B	HNF1 homeobox B	< 0.001	-1.70
HOXA2	homeobox A2	0.001	-2.30
HOXB8	homeobox B8	0.002	-1.95
HPRT1	hypoxanthine phosphoribosyltransferase 1	0.001	1.72
HPSE	heparanase	0.003	3.18
HRK	harakiri, BCL2 interacting protein (contains only BH3 domain)	< 0.001	-2.00
HSBP1L1	heat shock factor binding protein 1-like 1	0.006	-1.58
HSD17B12	hydroxysteroid (17-beta) dehydrogenase 12	0.001	1.51
HSPB8	heat shock 22kDa protein 8	< 0.001	1.76
HSPC159	lectin, galactoside-binding-like (LGALSL)	0.001	1.55
HSPE1	heat shock 10kDa protein 1 (chaperonin 10)	< 0.001	1.57
HTRA3	HtrA serine peptidase 3	< 0.001	-3.81
HUNK	hormonally up-regulated Neu-associated kinase	0.036	1.80
HYAL1	hyaluronoglucosaminidase 1	0.005	-1.55
ID2	inhibitor of DNA binding 2, dominant negative helix-loop-helix protein	0.001	1.82
IFI30	interferon, gamma-inducible protein 30	0.002	3.00
IFITM2	interferon induced transmembrane protein 2	0.004	-1.85
IFNW1	interferon, omega 1	0.002	-1.85
IGFBP2	insulin-like growth factor binding protein 2, 36kDa	0.034	2.13
IHH	Indian hedgehog	< 0.001	-2.55
IKZF1	IKAROS family zinc finger 1 (Ikaros)	0.003	2.13
IL15RA	interleukin 15 receptor, alpha	0.001	-1.70
IL6R	interleukin 6 receptor	0.007	-1.52

ILDR1	immunoglobulin-like domain containing receptor 1	< 0.001	-1.54
INPP1	inositol polyphosphate-1-phosphatase	0.001	-1.78
INPP5K	inositol polyphosphate-5-phosphatase K	< 0.001	-1.67
IP6K2	inositol hexakisphosphate kinase 2	0.001	-1.51
ISLR2	immunoglobulin superfamily containing leucine-rich repeat 2	0.003	1.92
ISM1	isthmin 1 homolog (zebrafish)	0.002	1.67
IST1	increased sodium tolerance 1 homolog (yeast)	< 0.001	-1.51
ITFG3	integrin alpha FG-GAP repeat containing 3	< 0.001	-2.05
ITGB4	integrin, beta 4	< 0.001	-1.95
JAM2	junctional adhesion molecule 2	0.003	4.13
JAM3	junctional adhesion molecule 3	0.002	1.62
KCND3	potassium voltage-gated channel, Shal-related subfamily, member 3	0.014	2.01
KCNH8	potassium voltage-gated channel, subfamily H (eag-related), member 8	0.010	-2.46
KCNIP3	Kv channel interacting protein 3, calsenilin	0.004	-2.72
KDM2B	lysine (K)-specific demethylase 2B	< 0.001	-1.57
KHDRBS3	KH domain containing, RNA binding, signal transduction associated 3	0.005	2.25
KIAA 1644	KIAA1644 ortholog	0.019	-1.89
KIAA0195	KIAA0195 ortholog	< 0.001	1.64
KIAA1147	KIAA1147 ortholog	0.002	-1.60
KIAA1462	KIAA1462 ortholog	0.001	3.29
KIAA2022	KIAA2022 ortholog	0.019	-1.60
KIT	v-kit Hardy-Zuckerman 4 feline sarcoma viral oncogene homolog	0.006	1.63
KLF11	Kruppel-like factor 11	< 0.001	-1.57
KLF17	Kruppel-like factor 17	< 0.001	-14.70
KLF3	Kruppel-like factor 3	< 0.001	-1.62
KLF5	Kruppel-like factor 5 (intestinal)	< 0.001	-1.57
KLHL5	kelch-like 5 (Drosophila)	0.008	-1.58
KLK12	kallikrein-related peptidase 12	0.001	-2.42
KLRF1	killer cell lectin-like receptor subfamily F, member 1	0.006	-1.83
KRT7	keratin 7	< 0.001	2.21
KSR2	kinase suppressor of ras 2	< 0.001	-8.94
LAMA1	laminin, alpha 1	0.005	1.76
LARP4	La ribonucleoprotein domain family, member 4	< 0.001	1.51
LCMT2	leucine carboxyl methyltransferase 2	< 0.001	1.71
LCOR	ligand dependent nuclear receptor corepressor	< 0.001	-1.61
LDLRAD1	low density lipoprotein receptor class A domain containing 1	0.002	-1.51
LEF1	lymphoid enhancer-binding factor 1	0.013	-1.99
LIFR	leukemia inhibitory factor receptor alpha	0.002	-1.54
LIN7A	lin-7 homolog A (C. elegans)	0.002	2.61

LMAN1L	lectin, mannose-binding, 1 like	0.004	5.54
LMNA	lamin A/C	< 0.001	1.73
LOC100125 412	differential display clone 8	0.007	-1.60
LOC100125 949	uncharacterized LOC100125949	0.022	-2.08
LOC100138 354	uncharacterized LOC100138354	< 0.001	-2.30
LOC100174 924	uncharacterized LOC100174924	0.008	1.50
LOC100296 081	uncharacterized LOC100296081	< 0.001	-2.77
LOC100296 441	uncharacterized LOC100296441	0.013	1.96
LOC100296 778	killer cell lectin-like receptor subfamily K, member 1-like	0.002	-1.65
LOC100297 336	uncharacterized LOC100297336	< 0.001	-1.76
LOC100301 416	melanoma antigen family B, 4-like	0.007	1.73
LOC100302 389	uncharacterized LOC100302389	0.001	-2.68
LOC100335 980	thymosin beta-4-like	0.025	-2.02
LOC100336 606	granulocyte-macrophage colony-stimulating factor receptor subunit alpha-like	< 0.001	-2.32
LOC100336 777	uncharacterized LOC100336777	< 0.001	-1.51
LOC100337 268	uncharacterized LOC100337268	< 0.001	-1.62
LOC100337 365	zinc finger protein 502-like	0.001	2.31
LOC100337 391	predicted protein-like	< 0.001	-7.77
LOC506694	delta-like protein 1-like	0.001	-1.62
LOC510716	natural resistance-associated macrophage protein 2-like	< 0.001	3.10
LOC513099	olfactory receptor, family 13, subfamily C, member 2-like	0.001	-2.01
LOC516108	CG2446-like	< 0.001	1.50
LOC518495	apolipoprotein L3-like	< 0.001	-1.87
LOC522479	ovalbumin-like	< 0.001	-5.34
LOC523139	olfactory receptor 481-like	0.002	-1.59
LOC526431	OTU domain containing 7A-like	0.001	1.63
LOC528722	olfactory receptor, family 5, subfamily D, member 13-like	0.010	-1.78
LOC531699	aldehyde oxidase 1-like	0.013	1.78
LOC531822	zinc finger protein 385D-like	0.003	-1.91
LOC537017	cytidine monophosphate-N-acetylneuraminic acid hydroxylase-like	0.015	-1.64
LOC537655	dystrophin-like	0.021	1.56
LOC615808	olfactory receptor, family 9, subfamily I, member 1-like	< 0.001	-2.62
LOC616840	ribosomal protein L3 pseudogene	0.001	-1.52

LOC617737	chromosome unknown open reading frame	0.035	-1.70
LOC781059	RAN, member RAS oncogene family pseudogene	0.002	2.03
LOC782470	tropomyosin 1 (alpha) pseudogene	0.005	1.60
LOC782521	proteasome (prosome, macropain) activator subunit 1 (PA28 alpha) pseudogene	0.015	1.54
LOC782865	olfactory receptor 481-like	0.006	-2.01
LOC783399	major allergen Equ c 1-like	0.043	2.41
LOC783604	guanylate-binding protein 4-like	< 0.001	2.80
LOC783649	trace amine-associated receptor 6-like	0.007	1.69
LOC784026	uncharacterized LOC784026	0.001	1.61
LOC785386	ribosomal protein L10-like	0.001	-1.75
LOC785429	transcriptional adaptor 2A pseudogene	0.007	-1.89
LOC785630	zinc finger protein 480-like	< 0.001	-2.05
LOC785717	sulfotransferase family, cytosolic, 1C, member 1-like	0.017	1.53
LOC785857	myosin VIIA and Rab interacting protein-like	0.015	-2.68
LOC787369	olfactory receptor, family 5, subfamily D, member 13-like	0.002	-1.68
LOC788874	olfactory receptor, family 5, subfamily F, member 1-like	0.003	-1.54
LOC789103	pRAME family member 9-like	0.001	1.67
LOC789201	olfactory receptor, family 4, subfamily A, member 47-like	< 0.001	1.64
LOC789815	olfactory receptor, family 13, subfamily C, member 2-like	< 0.001	-2.34
LOC789943	olfactory receptor, family 13, subfamily C, member 2-like	0.002	-3.12
LONRF3	LON peptidase N-terminal domain and ring finger 3	0.001	3.34
LOXL4	lysyl oxidase-like 4	0.008	-1.64
LPCAT2	lysophosphatidylcholine acyltransferase 2	0.001	1.91
LRAT	lecithin retinol acyltransferase (phosphatidylcholine--retinol O-acyltransferase)	0.011	-1.53
LRCH1	leucine-rich repeats and calponin homology (CH) domain containing 1	< 0.001	-1.63
LRP4	low density lipoprotein receptor-related protein 4	0.004	1.64
LRP5	low density lipoprotein receptor-related protein 5	< 0.001	-1.50
LRPAP1	low density lipoprotein receptor-related protein associated protein 1	< 0.001	1.75
LRRC26	leucine rich repeat containing 26	0.022	-1.58
LRRN1	leucine rich repeat neuronal 1	0.049	-1.87
LTA4H	leukotriene A4 hydrolase	0.001	1.53
LTBP3	latent transforming growth factor beta binding protein 3	0.001	-1.51
LYRM2	LYR motif containing 2	0.001	1.67
LYVE1	lymphatic vessel endothelial hyaluronan receptor 1	0.022	-1.56
MAGEE2	melanoma antigen family E, 2	0.009	1.55
MAGI1	membrane associated guanylate kinase, WW and PDZ domain containing 1	< 0.001	-1.58
MAML2	mastermind-like 2 (Drosophila)	0.001	-1.76
MANBA	mannosidase, beta A, lysosomal	< 0.001	-1.71

MANSC1	MANSC domain containing 1	0.001	1.55
MAP2	microtubule-associated protein 2	< 0.001	-2.95
MAP3K13	mitogen-activated protein kinase kinase kinase 13	< 0.001	-1.68
MAP4K3	mitogen-activated protein kinase kinase kinase kinase 3	0.001	-1.53
MARK2	MAP/microtubule affinity-regulating kinase 2	< 0.001	-1.65
MASP1	mannan-binding lectin serine peptidase 1 (C4/C2 activating component of Ra-reactive factor)	0.026	1.95
MBTD1	mbt domain containing 1	< 0.001	-1.70
MCF2L2	MCF.2 cell line derived transforming sequence-like 2	0.008	-1.51
MCTP1	multiple C2 domains, transmembrane 1	0.002	-1.96
MDK	midkine (neurite growth-promoting factor 2)	0.028	-2.19
MEIS2	Meis homeobox 2	< 0.001	1.92
MERTK	c-mer proto-oncogene tyrosine kinase	< 0.001	-2.23
MFAP4	microfibrillar-associated protein 4	0.032	2.31
MFAP5	microfibrillar associated protein 5	0.009	3.49
MGAT3	mannosyl (beta-1,4-)-glycoprotein beta-1,4-N-acetylglucosaminyltransferase	0.022	-1.69
MGAT4C	mannosyl (alpha-1,3-)-glycoprotein beta-1,4-N-acetylglucosaminyltransferase, isozyme C (putative)	< 0.001	-3.18
MGC133804	uncharacterized protein MGC133804	0.001	4.76
MGC134087	UPF0686 protein C11orf1 homolog-like	0.002	1.68
MGC148609	ankyrin repeat domain 65 (ANKRD65)	0.012	1.73
MGC151586	suppressor APC domain containing 1 (SAPCD1)	0.018	1.77
MGC165939	KIAA0226-like ortholog (KIAA0226L)	0.010	-2.45
MIR2305	microRNA mir-2305	< 0.001	-2.00
MIR2332	microRNA mir-2332	0.009	-1.67
MIR24-1	microRNA mir-24-1	0.005	-2.58
MIR2419	microRNA mir-2419	0.005	-1.68
MIR2440	microRNA mir-2440	< 0.001	-2.65
MIR30E	microRNA mir-30e	< 0.001	-1.65
MIR429	microRNA mir-429	0.003	-2.60
MIR452	microRNA mir-452	0.046	-1.74
MIR487B	microRNA mir-487b	< 0.001	-1.53
MIR582	microRNA mir-582	0.013	-2.81
MIR592	microRNA mir-592	0.006	1.73
MIR763	microRNA mir-763	< 0.001	-1.67
MKL2	MKL/myocardin-like 2	< 0.001	-1.61
MMRN2	multimerin 2	0.011	-1.69
MOBP	myelin-associated oligodendrocyte basic protein	0.011	-1.58
MORC4	MORC family CW-type zinc finger 4	0.006	1.62
MOXD1	monooxygenase, DBH-like 1	0.035	-1.66
MPHOSPH6	M-phase phosphoprotein 6	< 0.001	1.77

MPP6	membrane protein, palmitoylated 6 (MAGUK p55 subfamily member 6)	< 0.001	1.60
MPTX	putative mucosal pentraxin homolog	0.002	-3.40
MSH5	mutS homolog 5 (E. coli)	0.010	1.77
MSH6	mutS homolog 6 (E. coli)	< 0.001	-1.66
MSI1	musashi homolog 1 (Drosophila)	0.004	-1.75
MSLN	mesothelin	0.029	2.20
MTHFD1L	methylenetetrahydrofolate dehydrogenase (NADP+ dependent) 1-like	< 0.001	1.92
MTHFD2L	methylenetetrahydrofolate dehydrogenase (NADP+ dependent) 2-like	0.004	-1.52
MTHFR	methylenetetrahydrofolate reductase (NAD(P)H)	< 0.001	-1.94
MTRR	5-methyltetrahydrofolate-homocysteine methyltransferase reductase	< 0.001	-1.52
MX1	myxovirus (influenza virus) resistance 1, interferon-inducible protein p78 (mouse)	0.005	-2.52
MXI1	MAX interactor 1	< 0.001	-1.56
MXRA7	matrix-remodelling associated 7	0.001	-1.74
MYCN	v-myc myelocytomatosis viral related oncogene, neuroblastoma derived (avian)	< 0.001	-1.64
MYH11	myosin, heavy chain 11, smooth muscle	0.001	-2.40
MYL12B	myosin, light chain 12B, regulatory	< 0.001	1.59
MYO10	myosin X	< 0.001	-2.28
MYO9A	myosin IXA	0.004	-1.55
MYOM3	myomesin family, member 3	< 0.001	3.58
MZT1	mitotic spindle organizing protein 1	0.002	1.52
N4BP3	NEDD4 binding protein 3	< 0.001	1.52
NAGLU	N-acetylglucosaminidase, alpha	0.002	1.57
NCOA1	nuclear receptor coactivator 1	< 0.001	-1.69
NDP	Norrie disease (pseudoglioma)	< 0.001	-4.34
NDRG4	NDRG family member 4	< 0.001	-2.93
NDUFA1	NADH dehydrogenase (ubiquinone) 1 alpha subcomplex, 1, 7.5kDa	< 0.001	1.62
NDUFA12	NADH dehydrogenase (ubiquinone) 1 alpha subcomplex, 12	< 0.001	1.59
NDUFB5	NADH dehydrogenase (ubiquinone) 1 beta subcomplex, 5, 16kDa	< 0.001	1.56
NDUFS4	NADH dehydrogenase (ubiquinone) Fe-S protein 4, 18kDa (NADH-coenzyme Q reductase)	< 0.001	1.58
NENF	neudesin neurotrophic factor	0.006	1.54
NIM1	serine/threonine-protein kinase NIM1	0.007	-2.01
NIPA1	non imprinted in Prader-Willi/Angelman syndrome 1	< 0.001	-1.90
NIPAL2	NIPA-like domain containing 2	< 0.001	1.82
NIPAL3	NIPA-like domain containing 3	0.001	-1.57
NKAIN1	Na+/K+ transporting ATPase interacting 1	0.002	-2.73
NMB	neuromedin B	0.006	1.94
NOS1	nitric oxide synthase 1 (neuronal)	< 0.001	-1.99

NOSTRIN	nitric oxide synthase trafficker	0.005	-1.88
NOTCH1	notch 1	0.001	-1.73
NP aka PNP	purine nucleoside phosphorylase	< 0.001	-1.92
NPC2	Niemann-Pick disease, type C2	< 0.001	1.52
NRAP	nebulin-related anchoring protein	0.007	-2.38
NRG3	neuregulin 3	0.033	1.63
NRGN	neurogranin (protein kinase C substrate, RC3)	0.009	-1.58
NTM	neurotrimin	0.003	4.57
NTN1	netrin 1	0.003	-1.57
NTN4	netrin 4	0.005	-2.13
NTRK2	neurotrophic tyrosine kinase, receptor, type 2	0.004	4.00
NTRK3	neurotrophic tyrosine kinase, receptor, type 3	0.002	2.10
NUAK2	NUAK family, SNF1-like kinase, 2	< 0.001	-1.92
NUDT4	nudix (nucleoside diphosphate linked moiety X)-type motif 4	< 0.001	1.51
NUDT5	nudix (nucleoside diphosphate linked moiety X)-type motif 5	< 0.001	1.56
NUMB	numb homolog (Drosophila)	< 0.001	-1.52
NUP205	nucleoporin 205kDa	< 0.001	1.56
NUPR1	nuclear protein, transcriptional regulator, 1	0.014	-1.57
OAT	ornithine aminotransferase	0.001	2.08
OCA2	P protein-like	0.005	-2.80
OCLN	occludin	0.003	-1.52
ODZ1	odz, odd Oz/ten-m homolog 1 (Drosophila)	0.026	-1.87
ODZ3	odz, odd Oz/ten-m homolog 3 (Drosophila)	< 0.001	-1.72
OMG	oligodendrocyte myelin glycoprotein	0.029	-1.98
OPTC	opticin	0.007	1.58
OR5D13	olfactory receptor, family 5, subfamily D, member 13	< 0.001	-2.23
OR6Q1	olfactory receptor, family 6, subfamily Q, member 1	< 0.001	-2.13
OR9Q2	olfactory receptor, family 9, subfamily Q, member 2	0.001	-6.87
ORAI2	ORAI calcium release-activated calcium modulator 2	< 0.001	-2.18
OSBPL3	oxysterol binding protein-like 3	0.025	-1.62
OST4	oligosaccharyltransferase 4 homolog (S. cerevisiae)	< 0.001	1.70
OSTN	osteocrin	< 0.001	5.02
P2RX2	purinergic receptor P2X, ligand-gated ion channel, 2	0.002	6.86
P2RY2	purinergic receptor P2Y, G-protein coupled, 2	< 0.001	-1.74
PACRGL	PARK2 co-regulated-like	< 0.001	1.88
PACS1	phosphofurin acidic cluster sorting protein 1	< 0.001	-1.80
PARM1	prostate androgen-regulated mucin-like protein 1	0.002	-1.89
PAX6	paired box 6	0.040	1.82
PCGF2	polycomb group ring finger 2	< 0.001	-1.53
PDE1C	phosphodiesterase 1C, calmodulin-dependent 70kDa	0.003	1.98
PDE4D	phosphodiesterase 4D, cAMP-specific	0.002	-2.23

PDGFA	platelet-derived growth factor alpha polypeptide	< 0.001	-1.83
PDK1	pyruvate dehydrogenase kinase, isozyme 1	0.002	1.79
PDSS1	prenyl (decaprenyl) diphosphate synthase, subunit 1	0.003	1.64
PDXK	pyridoxal (pyridoxine, vitamin B6) kinase	0.003	-1.76
PDYN	prodynorphin	0.042	2.36
PDZK1	PDZ domain containing 1	0.013	-3.77
PDZRN3	PDZ domain containing ring finger 3	0.001	-1.93
PEG3	paternally expressed 3	< 0.001	-1.53
PET100	PET100 homolog (S. cerevisiae)	< 0.001	1.55
PFKFB1	6-phosphofructo-2-kinase/fructose-2,6-biphosphatase 1	0.001	-1.87
PGK1	phosphoglycerate kinase 1	< 0.001	1.77
PHB	prohibitin	< 0.001	1.97
PIK3C2G	phosphoinositide-3-kinase, class 2, gamma polypeptide	0.002	-3.00
PIK3IP1	phosphoinositide-3-kinase interacting protein 1	< 0.001	-1.61
PIR	pirin (iron-binding nuclear protein)	0.002	1.85
PITX1	paired-like homeodomain 1	0.004	1.82
PIWIL1	piwi-like RNA-mediated gene silencing 1	0.002	-1.74
PKHD1L1	polycystic kidney and hepatic disease 1 (autosomal recessive)-like 1	0.004	9.86
PKIB	protein kinase (cAMP-dependent, catalytic) inhibitor beta	0.001	1.87
PKM2	pyruvate kinase, muscle	< 0.001	1.61
PKP4	plakophilin 4	< 0.001	-1.52
PLA2G6	phospholipase A2, group VI (cytosolic, calcium-independent)	< 0.001	-1.52
PLAU	plasminogen activator, urokinase	0.003	2.01
PLBD2	phospholipase B domain containing 2	< 0.001	1.56
PLCB1	phospholipase C, beta 1 (phosphoinositide-specific)	0.026	-1.86
PLCE1	phospholipase C, epsilon 1	0.002	1.97
PLD1	phospholipase D1, phosphatidylcholine-specific	< 0.001	-2.19
PLEKHG1	pleckstrin homology domain containing, family G (with RhoGef domain) member 1	0.007	1.87
PLLP	plasmolipin	0.002	-1.70
PLOD2	procollagen-lysine, 2-oxoglutarate 5-dioxygenase 2	< 0.001	2.07
PLUNC	BPI fold containing family A, member 1 (BPIFA1)	0.001	-3.81
POLR3B	polymerase (RNA) III (DNA directed) polypeptide B	< 0.001	1.52
PPEF1	protein phosphatase, EF-hand calcium binding domain 1	0.004	1.75
PPEF2	protein phosphatase, EF-hand calcium binding domain 2	0.008	-1.70
PPIL1	peptidylprolyl isomerase (cyclophilin)-like 1	0.001	1.60
PPM1H	protein phosphatase, Mg2+/Mn2+ dependent, 1H	0.001	-1.61
PPP1R13B	protein phosphatase 1, regulatory subunit 13B	< 0.001	-1.99
PPP2R2B	protein phosphatase 2, regulatory subunit B, beta	0.012	2.15
PPP2R2C	protein phosphatase 2, regulatory subunit B, gamma	< 0.001	7.30
PRCP	prolylcarboxypeptidase (angiotensinase C)	0.001	1.55

PRDM1	PR domain containing 1, with ZNF domain	0.002	-3.95
PRDX5	peroxiredoxin 5	< 0.001	1.54
PRELP	proline/arginine-rich end leucine-rich repeat protein	< 0.001	2.44
PRKCE	protein kinase C, epsilon	< 0.001	-1.68
PRLR	prolactin receptor	< 0.001	5.17
PROM2	prominin 2	0.017	-1.78
PRR5L	proline rich 5 like	0.001	-2.19
PRSS16	protease, serine, 16 (thymus)	0.011	-3.12
PRSS23	protease, serine, 23	0.001	2.77
PSMB3	proteasome (prosome, macropain) subunit, beta type, 3	< 0.001	1.55
PSMD6	proteasome (prosome, macropain) 26S subunit, non-ATPase, 6	< 0.001	1.50
PTCH2	patched 2	0.004	-1.62
PTDSS1	phosphatidylserine synthase 1	< 0.001	1.58
PTPRM	protein tyrosine phosphatase, receptor type, M	< 0.001	-1.79
PVR	poliovirus receptor	< 0.001	1.69
PVRL1	poliovirus receptor-related 1 (herpesvirus entry mediator C)	< 0.001	1.85
PXK	PX domain containing serine/threonine kinase	0.001	1.63
QPCT	glutaminyl-peptide cyclotransferase	0.003	1.87
RAB24	RAB24, member RAS oncogene family	< 0.001	-1.66
RABEP1	rabaptin, RAB GTPase binding effector protein 1	< 0.001	-1.54
RACGAP1	Rac GTPase activating protein 1	0.015	1.77
RAD18	RAD18 homolog (S. cerevisiae)	0.002	1.62
RAI2	retinoic acid induced 2	0.002	-1.58
RALGPS1	Ral GEF with PH domain and SH3 binding motif 1	< 0.001	-1.60
RAP1GDS1	RAP1, GTP-GDP dissociation stimulator 1	< 0.001	1.50
RAPGEF4	Rap guanine nucleotide exchange factor (GEF) 4	0.002	1.69
RAPGEF5	Rap guanine nucleotide exchange factor (GEF) 5	< 0.001	1.72
RARRES2	retinoic acid receptor responder (tazarotene induced) 2	0.005	-1.56
RASA3	RAS p21 protein activator 3	< 0.001	1.56
RASGRP3	RAS guanyl releasing protein 3 (calcium and DAG-regulated)	0.010	-2.41
RASSF6	Ras association (RalGDS/AF-6) domain family member 6	< 0.001	-1.57
RBFOX3	RNA binding protein, fox-1 homolog (C. elegans) 3	0.003	1.58
RBM11	RNA binding motif protein 11	< 0.001	1.54
RELN	reelin	0.005	-2.16
REV3L	REV3-like, catalytic subunit of DNA polymerase zeta (yeast)	< 0.001	-1.82
RFK	riboflavin kinase	0.007	1.57
RGMB	RGM domain family, member B	0.001	-1.68
RGN	regucalcin (senescence marker protein-30)	0.002	-1.56
RGNEF	190 kDa guanine nucleotide exchange factor	< 0.001	-1.89
RGS11	regulator of G-protein signaling 11	< 0.001	-1.56
RGS9	regulator of G-protein signaling 9	0.001	-1.53

RHOBTB1	Rho-related BTB domain containing 1	0.003	1.59
RHOJ	ras homolog gene family, member J	0.041	-2.17
RHOQ	ras homolog gene family, member Q	0.008	1.52
RIN2	Ras and Rab interactor 2	< 0.001	-1.91
RNASE1	ribonuclease, RNase A family, 1 (pancreatic)	0.044	-1.88
RNASE11	ribonuclease, RNase A family, 11 (non-active)	0.002	-2.53
RNF157	ring finger protein 157	< 0.001	-1.72
RNF38	ring finger protein 38	< 0.001	-1.57
RNF43	ring finger protein 43	0.004	-1.84
RNLS	renalase, FAD-dependent amine oxidase	0.002	1.50
ROBO1	roundabout, axon guidance receptor, homolog 1 (Drosophila)	0.002	-2.83
ROR1	receptor tyrosine kinase-like orphan receptor 1	0.041	1.54
ROR2	receptor tyrosine kinase-like orphan receptor 2	0.004	1.77
RPA3	replication protein A3, 14kDa	< 0.001	1.66
RRAS	related RAS viral (r-ras) oncogene homolog	0.001	1.64
S100A11	S100 calcium binding protein A11	0.003	1.81
S100A16	S100 calcium binding protein A16	0.003	4.60
S100B	S100 calcium binding protein B	0.003	1.92
S100G	S100 calcium binding protein G	0.019	-2.90
SAMD12	sterile alpha motif domain containing 12	0.002	1.53
SAMD5	sterile alpha motif domain containing 5	0.004	-1.80
SAT2	spermidine/spermine N1-acetyltransferase family member 2	0.003	-1.58
SCD5	stearoyl-CoA desaturase 5	0.001	1.52
SCN3A	sodium channel, voltage-gated, type III, alpha subunit	0.022	-2.41
SCUBE1	signal peptide, CUB domain, EGF-like 1	< 0.001	-1.68
SDC4	syndecan 4	0.002	-1.72
SEC14L1	SEC14-like 1 (S. cerevisiae)	0.001	-1.96
SEH1L	SEH1-like (S. cerevisiae)	< 0.001	1.56
SELH	chromosome 15 open reading frame, human C11orf31	0.004	-1.67
SEMA3A	sema domain, immunoglobulin domain (Ig), short basic domain, secreted, (semaphorin) 3A	0.001	4.50
SEMA4C	sema domain, immunoglobulin domain (Ig), transmembrane domain (TM) and short cytoplasmic domain, (semaphorin) 4C	0.001	-2.01
SEMA4D	sema domain, immunoglobulin domain (Ig), transmembrane domain (TM) and short cytoplasmic domain, (semaphorin) 4D	< 0.001	-2.02
SEMA4G	sema domain, immunoglobulin domain (Ig), transmembrane domain (TM) and short cytoplasmic domain, (semaphorin) 4G	< 0.001	-1.52
SEMA5A	sema domain, seven thrombospondin repeats (type 1 and type 1-like), transmembrane domain (TM) and short cytoplasmic domain, (semaphorin) 5A	< 0.001	-4.26
SEMA6D	sema domain, transmembrane domain (TM), and cytoplasmic domain, (semaphorin) 6D	0.001	-2.43
SERPINB5	serpin peptidase inhibitor, clade B (ovalbumin), member 5	< 0.001	1.63
SERPINB7	serpin peptidase inhibitor, clade B (ovalbumin), member 7	0.025	-3.86

SERTAD4	SERTA domain containing 4	0.001	-1.78
SETD7	SET domain containing (lysine methyltransferase) 7	< 0.001	-1.53
SETX	senataxin	< 0.001	-2.22
SGCZ	sarcoglycan, zeta	0.006	-1.84
SGSM1	small G protein signaling modulator 1	< 0.001	-1.82
SH3RF2	protein phosphatase 1, regulatory subunit 39 (PPP1R39)	0.001	-1.57
SIRPA	signal-regulatory protein alpha	0.005	-1.55
SIRT2	sirtuin 2	0.015	1.56
SLAMF9	SLAM family member 9	0.036	-2.32
SLC12A6	solute carrier family 12 (potassium/chloride transporters), member 6	0.003	-1.56
SLC12A7	solute carrier family 12 (potassium/chloride transporters), member 7	0.001	-1.54
SLC16A11	solute carrier family 16, member 11 (monocarboxylic acid transporter 11)	0.001	-1.72
SLC18A2	solute carrier family 18 (vesicular monoamine), member 2	0.014	1.83
SLC1A4	solute carrier family 1 (glutamate/neutral amino acid transporter), member 4	0.011	-1.64
SLC22A5	solute carrier family 22 (organic cation/carnitine transporter), member 5	< 0.001	-1.59
SLC24A6	solute carrier family 24 (sodium/potassium/calcium exchanger), member 6	0.008	-1.57
SLC25A42	solute carrier family 25, member 42	0.001	-1.69
SLC27A2	solute carrier family 27 (fatty acid transporter), member 2	0.019	-1.70
SLC2A12	solute carrier family 2 (facilitated glucose transporter), member 12	0.004	-1.67
SLC30A10	solute carrier family 30, member 10	0.005	-1.53
SLC31A2	solute carrier family 31 (copper transporters), member 2	< 0.001	-4.06
SLC35F3	solute carrier family 35, member F3	0.003	-2.52
SLC37A1	solute carrier family 37 (glycerol-3-phosphate transporter), member 1	0.004	-1.52
SLC38A7	solute carrier family 38, member 7	< 0.001	-1.56
SLC39A14	solute carrier family 39 (zinc transporter), member 14	0.004	2.90
SLC5A9	solute carrier family 5 (sodium/glucose cotransporter), member 9	0.016	-1.68
SLC7A6	solute carrier family 7 (amino acid transporter light chain, y+L system), member 6	0.003	1.81
SLC7A7	solute carrier family 7 (amino acid transporter light chain, y+L system), member 7	< 0.001	-1.54
SLCO4A1	solute carrier organic anion transporter family, member 4A1	0.004	1.57
SLITRK2	SLIT and NTRK-like family, member 2	0.009	2.17
SLMO2	slowmo homolog 2 (Drosophila)	< 0.001	1.53
SMPX	small muscle protein, X-linked	0.001	-2.56
SMS	spermine synthase	0.003	-1.71
SNAP91	synaptosomal-associated protein, 91kDa homolog (mouse)	0.005	-2.21
SNX33	sorting nexin 33	< 0.001	-1.56
SORBS2	sorbin and SH3 domain containing 2	< 0.001	-1.60

SOX4	SRY (sex determining region Y)-box 4	< 0.001	-1.51
SOX6	SRY (sex determining region Y)-box 6	0.002	-1.73
SPARC	secreted protein, acidic, cysteine-rich (osteonectin)	0.003	4.99
SPATA5L1	spermatogenesis associated 5-like 1	< 0.001	-1.72
SPON1	spondin 1, extracellular matrix protein	< 0.001	4.70
SPRY2	sprouty homolog 2 (Drosophila)	0.001	-1.57
SPRY3	sprouty homolog 3 (Drosophila)	< 0.001	-3.50
SRGAP2	SLIT-ROBO Rho GTPase activating protein 2	< 0.001	-1.69
ST3GAL1	ST3 beta-galactoside alpha-2,3-sialyltransferase 1	< 0.001	3.91
ST5	suppression of tumorigenicity 5	< 0.001	-1.52
ST6GAL1	ST6 beta-galactosamide alpha-2,6-sialyltranferase 1	0.001	2.64
ST6GALNAC6	ST6 (alpha-N-acetyl-neuraminyl-2,3-beta-galactosyl-1,3)-N-acetylgalactosaminide alpha-2,6-sialyltransferase 6	0.003	1.61
STARD13	StAR-related lipid transfer (START) domain containing 13	0.003	-1.65
STARD4	StAR-related lipid transfer (START) domain containing 4	< 0.001	1.53
STEAP3	STEAP family member 3, metalloreductase	< 0.001	1.74
STK17B	serine/threonine kinase 17b	< 0.001	-1.77
STK39	serine threonine kinase 39	0.008	1.64
STT3B	STT3, subunit of the oligosaccharyltransferase complex, homolog B (S. cerevisiae)	< 0.001	1.53
SULF2	sulfatase 2	< 0.001	1.92
SULT1A1	sulfotransferase family, cytosolic, 1A, phenol-preferring, member 1	< 0.001	-2.79
SWAP70	SWAP switching B-cell complex 70kDa subunit	0.004	-1.81
SYT10	synaptotagmin X	0.002	1.79
SYTL5	synaptotagmin-like 5	0.001	-1.83
TAC1	tachykinin, precursor 1	0.027	2.48
TBL1X	transducin (beta)-like 1X-linked	0.001	1.83
TBX3	T-box 3	0.007	2.21
TCF7	transcription factor 7 (T-cell specific, HMG-box)	0.001	-2.25
TCF7L1	transcription factor 7-like 1 (T-cell specific, HMG-box)	0.027	-1.53
TEC	tec protein tyrosine kinase	0.001	-1.53
TFPI	tissue factor pathway inhibitor (lipoprotein-associated coagulation inhibitor)	0.007	-2.79
TGFA	transforming growth factor, alpha	< 0.001	2.05
TGFB3	transforming growth factor, beta 3	0.001	2.87
TGFBI	transforming growth factor, beta-induced, 68kDa	0.011	2.11
TGS1	trimethylguanosine synthase 1	< 0.001	1.55
THBS1	thrombospondin 1	0.017	-2.20
THBS2	thrombospondin 2	< 0.001	1.72
THEM5	thioesterase superfamily member 5	0.032	1.81
TLR3	toll-like receptor 3	0.009	-1.56
TLR5	toll-like receptor 5	0.002	2.72

TM4SF1	transmembrane 4 L six family member 1	0.008	2.15
TM7SF3	transmembrane 7 superfamily member 3	< 0.001	-1.51
TMED6	transmembrane emp24 protein transport domain containing 6	0.007	-1.84
TMED8	transmembrane emp24 protein transport domain containing 8	< 0.001	-1.54
TMEM120B	transmembrane protein 120B	0.003	1.95
TMEM150C	transmembrane protein 150C	0.013	5.14
TMEM154	transmembrane protein 154	0.013	1.68
TMEM195	alkylglycerol monooxygenase (AGMO)	0.020	1.67
TMEM204	transmembrane protein 204	0.015	1.62
TMEM213	transmembrane protein 213	0.001	3.47
TMEM52	transmembrane protein 52	0.008	1.64
TMTC1	transmembrane and tetratricopeptide repeat containing 1	< 0.001	-2.11
TMTC2	transmembrane and tetratricopeptide repeat containing 2	0.023	-1.71
TNFRSF18	tumor necrosis factor receptor superfamily, member 18	0.023	1.70
TOX	thymocyte selection-associated high mobility group box	0.009	-1.71
TPM4	tropomyosin 4	0.002	1.96
TRAF3IP2	TRAF3 interacting protein 2	< 0.001	-1.57
TRAM1	translocation associated membrane protein 1	< 0.001	1.51
TRIM21	tripartite motif containing 21	< 0.001	-1.57
TRIM68	tripartite motif containing 68	0.001	-1.65
TRIM6-TRIM34	tripartite motif containing 6- tripartite motif containing 34	< 0.001	-1.53
TRIM9	tripartite motif containing 9	< 0.001	-2.74
TRIO	triple functional domain (PTPRF interacting)	< 0.001	-1.54
TRIP10	thyroid hormone receptor interactor 10	< 0.001	-1.61
TRIP6	thyroid hormone receptor interactor 6	< 0.001	1.50
TSC1	tuberous sclerosis 1	< 0.001	-1.54
TSC22D3	TSC22 domain family, member 3	0.001	-1.72
TSKU	tsukushi small leucine rich proteoglycan homolog (Xenopus laevis)	0.002	-1.75
TSPAN12	tetraspanin 12	0.001	-2.22
TSPAN2	tetraspanin 2	0.015	-1.76
TTC38	tetratricopeptide repeat domain 38	< 0.001	1.71
TTC39A	tetratricopeptide repeat domain 39A	0.013	-1.52
TTF1	transcription termination factor, RNA polymerase I	0.001	-1.50
TXN	thioredoxin	< 0.001	1.70
UACA	uveal autoantigen with coiled-coil domains and ankyrin repeats	0.001	-1.60
UBALD2	UBA-like domain containing 2	< 0.001	-1.74
UBE2N	ubiquitin-conjugating enzyme E2N	< 0.001	1.75
UBTD1	ubiquitin domain containing 1	< 0.001	1.51
UGT1A1	UDP glucuronosyltransferase 1 family, polypeptide A1	< 0.001	-3.67
UNC5B	unc-5 homolog B (C. elegans)	0.003	-2.08

USMG5	up-regulated during skeletal muscle growth 5 homolog (mouse)	0.003	1.57
USP18	ubiquitin specific peptidase 18	0.001	-1.81
USP2	ubiquitin specific peptidase 2	0.001	1.60
USP20	ubiquitin specific peptidase 20	< 0.001	-1.52
VAT1L	vesicle amine transport protein 1 homolog (T. californica)-like	< 0.001	5.22
VSTM2A	V-set and transmembrane domain containing 2A	0.006	-1.50
WARS	tryptophanyl-tRNA synthetase	0.018	1.78
WDFY2	WD repeat and FYVE domain containing 2	0.017	-1.51
WDR87	WD repeat domain 87	0.001	-1.95
WIF1	WNT inhibitory factor 1	0.042	-1.99
WLS	wntless homolog (Drosophila)	0.002	-1.77
WNK3	WNK lysine deficient protein kinase 3	0.009	-1.76
WNT5A	wingless-type MMTV integration site family, member 5A	0.001	-2.02
WSF1	Wolfram syndrome 1 (wolframin)	< 0.001	2.15
WT1	Wilms tumor 1	0.005	2.68
XAF1	XIAP associated factor 1	< 0.001	-1.69
XG	Xg blood group	0.002	-1.60
XK	X-linked Kx blood group (McLeod syndrome)	0.003	1.74
YPEL1	yippee-like 1 (Drosophila)	0.001	-1.77
ZAR1L	zygote arrest 1-like	< 0.001	-1.55
ZDHHC21	zinc finger, DHHC-type containing 21	< 0.001	1.52
ZFYVE9	zinc finger, FYVE domain containing 9	< 0.001	-1.82
ZMYM3	zinc finger, MYM-type 3	< 0.001	-1.63
ZNF114	zinc finger protein 114	0.002	1.63
ZNF235	zinc finger protein 235	< 0.001	-1.87
ZNF354A	zinc finger protein 354A	0.001	-1.50
ZNF395	zinc finger protein 395	< 0.001	-1.52
ZNF398	zinc finger protein 398	< 0.001	-1.75
ZNF454	zinc finger protein 454	0.003	-1.58
ZNF639	zinc finger protein 639	0.004	-1.58
ZNHIT6	zinc finger, HIT-type containing 6	0.001	1.62
ZSCAN21	zinc finger and SCAN domain containing 21	< 0.001	-1.53
ZSWIM1	zinc finger, SWIM-type containing 1	0.002	-1.55
ZSWIM5	zinc finger, SWIM-type containing 5	0.001	-1.70
