# Supplementary material for: A transcriptomal analysis of bovine oviductal epithelial cells collected during the follicular phase versus the luteal phase of the estrous cycle
Source: Reprod Biol Endocrinol. 2015 Aug 5;13:84. doi: 10.1186/s12958-015-0077-1 (PMC4524109; doi:10.1186/s12958-015-0077-1)
Supplement: Additional file 4: — Supplementary Table 2. Differentially expressed transcripts in common between follicular and luteal phases of the estrous cycle in epithelial cells of the ampulla and isthmus (FDR adjusted P < 0.05). [file 12958_2015_77_MOESM4_ESM.pdf]

Supplementary Table 2. Differentially expressed transcripts in common between follicular and luteal phases of the estrous cycle in epithelial cells of the ampulla and isthmus (FDR adjusted P < 0.05).

		**FP vs. LP (Ampulla)**		**FP vs. LP (Isthmus)**	
**Gene Symbol**	**Gene Description**	**P-value**	**Fold-Change**	**P-value**	**Fold-change**
AACS	acetoacetyl-CoA synthetase	< 0.001	2.47	< 0.001	1.82
AADAC	arylacetamide deacetylase (esterase)	0.003	-1.84	0.008	-1.68
AADACL3	uncharacterized LOC530613	0.026	-1.59	0.003	-2.00
ABCA1	ATP-binding cassette, sub-family A (ABC1), member 1	0.001	-1.53	< 0.001	-3.56
ABCA6	ATP-binding cassette, sub-family A (ABC1), member 6	0.001	-2.51	0.036	-1.53
ABCA9	ATP-binding cassette, sub-family A (ABC1), member 9	< 0.001	4.57	< 0.001	4.37
ABCC4	ATP-binding cassette, sub-family C (CFTR/MRP), member 4	0.006	2.43	< 0.001	6.35
ABCC5	ATP-binding cassette, sub-family C (CFTR/MRP), member 5	< 0.001	-1.78	< 0.001	-1.72
ABCF2	ATP-binding cassette, sub-family F (GCN20), member 2	0.001	1.54	0.001	1.56
ABHD1	abhydrolase domain containing 1	0.001	-1.64	< 0.001	-2.20
ABHD5	abhydrolase domain containing 5	< 0.001	1.59	< 0.001	1.56
ABTB1	ankyrin repeat and BTB (POZ) domain containing 1	< 0.001	-2.28	< 0.001	-2.02
ACAT2	acetyl-CoA acetyltransferase 2	< 0.001	3.35	0.006	1.61
ACE	angiotensin I converting enzyme	0.003	-1.52	0.001	-1.62
ACTA2	actin, alpha 2, smooth muscle, aorta	< 0.001	2.32	0.002	1.96
ADA	adenosine deaminase	0.003	2.30	0.004	2.19
ADAM28	ADAM metallopeptidase domain 28	0.021	-1.80	0.004	-2.30
ADAMDEC1	ADAM-like, decysin 1	0.036	-2.24	0.018	-2.60
ADORA2B	adenosine A2b receptor	0.012	1.66	< 0.001	2.46
AGR2	anterior gradient homolog 2 (Xenopus laevis)	< 0.001	3.66	< 0.001	6.25
AGR3	anterior gradient 3 homolog (Xenopus laevis)	0.010	1.84	< 0.001	2.85
AGT	angiotensinogen (serpin peptidase inhibitor, clade A, member 8)	< 0.001	-1.81	< 0.001	-1.87
AK2	adenylate kinase 2	< 0.001	2.10	0.001	1.77
AKAP8L	A kinase (PRKA) anchor protein 8-like	< 0.001	-1.53	< 0.001	-1.75
ALCAM	activated leukocyte cell adhesion molecule	0.005	-1.51	0.001	-1.73
ALG13	asparagine-linked glycosylation 13 homolog (S. cerevisiae)	< 0.001	-2.43	0.001	-2.05
ALG3	asparagine-linked glycosylation 3, alpha-1,3-mannosyltransferase homolog (S. cerevisiae)	< 0.001	2.23	< 0.001	1.92
ALG5	asparagine-linked glycosylation 5, dolichyl-phosphate beta-glucosyltransferase homolog (S. cerevisiae)	< 0.001	1.91	< 0.001	1.91

ALOX12	arachidonate 12-lipoxygenase	0.046	2.07	0.005	3.33
ALS2CL	ALS2 C-terminal like	0.002	-1.56	0.001	-1.74
AMIGO3	adhesion molecule with Ig-like domain 3	< 0.001	3.78	< 0.001	2.49
ANG	angiogenin, ribonuclease, RNase A family, 5	0.020	-1.90	0.005	-2.34
ANK2	ankyrin 2, neuronal	0.001	-2.03	0.005	-1.69
ANKRD33B	ankyrin repeat domain 33B	0.001	-1.62	0.002	-1.55
ANKRD37	ankyrin repeat domain 37	< 0.001	-1.56	< 0.001	-1.52
ANXA6	annexin A6	< 0.001	4.39	< 0.001	4.31
AP3S1	adaptor-related protein complex 3, sigma 1 subunit	< 0.001	1.90	< 0.001	1.92
APH1B	anterior pharynx defective 1 homolog B (C. elegans)	0.006	-1.77	0.007	-1.72
APITD1	apoptosis-inducing, TAF9-like domain 1	0.002	1.71	0.003	1.62
APOD	apolipoprotein D	0.004	-2.04	0.014	-1.73
APOO	apolipoprotein O	0.002	1.56	0.001	1.67
APPL2	adaptor protein, phosphotyrosine interaction, PH domain and leucine zipper containing 2	< 0.001	-1.58	< 0.001	-1.62
ARF4	ADP-ribosylation factor 4	< 0.001	1.78	< 0.001	1.91
ARGLU1	arginine and glutamate rich 1	0.003	-1.54	0.001	-1.67
ARHGAP11 A	Rho GTPase activating protein 11A	0.014	2.37	0.016	2.33
ARHGAP20	Rho GTPase activating protein 20	0.002	-1.58	0.001	-1.68
ARHGDIA	Rho GDP dissociation inhibitor (GDI) alpha	< 0.001	1.85	< 0.001	1.63
ARPC3	actin related protein 2/3 complex, subunit 3, 21kDa	0.022	1.57	0.021	1.58
ARPC5L	actin related protein 2/3 complex, subunit 5-like	0.001	1.56	< 0.001	1.80
ARSD	arylsulfatase D	0.002	-2.15	0.004	-1.92
ASAM	CXADR-like membrane protein (CLMP)	0.002	1.60	0.003	1.54
ASF1B	ASF1 anti-silencing function 1 homolog B (S. cerevisiae)	< 0.001	2.86	0.012	1.78
ASNA1	arsA arsenite transporter, ATP-binding, homolog 1 (bacterial)	< 0.001	1.70	< 0.001	1.55
ASNS	asparagine synthetase (glutamine-hydrolyzing)	0.001	1.69	< 0.001	2.40
ASPA	aspartoacylase	0.001	-1.58	0.001	-1.60
ASS1	argininosuccinate synthase 1	0.003	1.94	0.007	1.76
ATG14	ATG14 autophagy related 14 homolog (S. cerevisiae)	0.001	-1.65	< 0.001	-1.78
ATG9B	autophagy related 9B	0.012	-1.79	0.009	-1.85
ATOX1	ATX1 antioxidant protein 1 homolog (yeast)	0.002	2.45	0.005	2.11
ATP13A4	ATPase type 13A4	0.003	-1.69	< 0.001	-3.40
ATP1B2	ATPase, Na+/K+ transporting, beta 2 polypeptide	0.042	-1.65	0.005	-2.19
ATP5G1	ATP synthase, H+ transporting, mitochondrial Fo complex, subunit C1 (subunit 9)	< 0.001	2.41	< 0.001	2.31
ATP5G3	ATP synthase, H+ transporting, mitochondrial Fo complex, subunit C3 (subunit 9)	< 0.001	1.83	< 0.001	1.81

ATP5I	ATP synthase, H+ transporting, mitochondrial Fo complex, subunit E	< 0.001	1.84	< 0.001	1.58
ATP5J	ATP synthase, H+ transporting, mitochondrial Fo complex, subunit F6	< 0.001	1.55	< 0.001	1.53
ATP5J2	ATP synthase, H+ transporting, mitochondrial Fo complex, subunit F2	< 0.001	1.54	< 0.001	1.67
ATP6V1B1	ATPase, H+ transporting, lysosomal 56/58kDa, V1 subunit B1	< 0.001	-2.64	< 0.001	-2.03
ATP8A1	ATPase, aminophospholipid transporter (APLT), class I, type 8A, member 1	< 0.001	-1.95	< 0.001	-1.95
B3GALT5	UDP-Gal:betaGlcNAc beta 1,3-galactosyltransferase, polypeptide 5	0.003	-1.63	0.006	-1.54
B3GAT2	beta-1,3-glucuronyltransferase 2	0.007	-1.59	0.007	-1.59
B3GNT8	UDP-GlcNAc:betaGal beta-1,3-N-acetylglucosaminyltransferase 8	< 0.001	1.80	< 0.001	1.94
B9D1	B9 protein domain 1	< 0.001	1.90	< 0.001	2.02
BAIAP2L2	BAI1-associated protein 2-like 2	0.027	-1.58	< 0.001	-2.62
BAX	BCL2-associated X protein	< 0.001	1.68	< 0.001	1.52
BCAT2	branched chain amino-acid transaminase 2, mitochondrial	< 0.001	2.28	< 0.001	1.67
BCCIP	BRCA2 and CDKN1A interacting protein	0.002	1.58	0.003	1.51
BCL2	B-cell CLL/lymphoma 2	< 0.001	-2.08	< 0.001	-2.13
BCL6	B-cell CLL/lymphoma 6	< 0.001	-1.95	< 0.001	-2.88
BCS1L	BCS1-like (S. cerevisiae)	< 0.001	1.89	< 0.001	1.78
BDH1	3-hydroxybutyrate dehydrogenase, type 1	< 0.001	3.48	< 0.001	2.56
BDKRB1	bradykinin receptor B1	< 0.001	-1.86	< 0.001	-1.68
BEX5	brain expressed, X-linked 5	0.004	1.52	0.001	1.68
BIN1	bridging integrator 1	0.001	1.79	< 0.001	2.57
BIRC5	baculoviral IAP repeat containing 5	0.006	1.75	0.028	1.51
BLNK	B-cell linker	0.008	-2.28	0.010	-2.19
BMP4	bone morphogenetic protein 4	0.001	-4.44	0.023	-2.32
BMPR1B	bone morphogenetic protein receptor, type IB	< 0.001	-1.53	< 0.001	-1.67
BORA	bora, aurora kinase A activator	< 0.001	1.77	< 0.001	1.65
BPIL2	BPI fold containing family C	0.005	-1.71	0.010	-1.59
BRB	brain ribonuclease	0.006	-2.20	0.019	-1.87
BRP44L	brain protein 44-like	< 0.001	1.60	< 0.001	1.63
BSPRY	B-box and SPRY domain containing	< 0.001	1.89	0.001	1.63
BTNL9	butyrophilin-like 9	0.002	-1.61	< 0.001	-1.59
BUB1	budding uninhibited by benzimidazoles 1 homolog (yeast)	0.003	1.70	0.002	1.84
BUB1B	budding uninhibited by benzimidazoles 1 homolog beta (yeast)	0.010	2.77	0.005	3.24
BYSL	bystin-like	< 0.001	1.65	< 0.001	1.64
C10H14ORF1	chromosome 10 open reading frame	< 0.001	2.51	< 0.001	1.83

C11H9orf16	chromosome 11 open reading frame	< 0.001	2.03	< 0.001	1.74
C13H20ORF27	chromosome 13 open reading frame	< 0.001	1.88	< 0.001	1.66
C18H16ORF61	COX assembly mitochondrial protein 2 homolog (S. cerevisiae) (CMC2)	0.003	1.61	< 0.001	2.28
C19H17orf37	migration and invasion enhancer 1 (MIEN1)	< 0.001	2.07	0.002	1.71
C19H17orf49	chromosome 19 open reading frame	< 0.001	1.70	< 0.001	1.64
C1orf110	coiled-coil domain-containing protein C1orf110 homolog	< 0.001	-2.06	< 0.001	-2.12
C1QA	complement component 1, q subcomponent, A chain	0.017	-1.76	0.010	-1.89
C1QB	complement component 1, q subcomponent, B chain	0.004	-2.32	0.006	-2.19
C1QTNF3	C1q and tumor necrosis factor related protein 3	0.023	1.77	0.011	1.97
C23H6orf129	coiled-coil domain containing 167 (CCDC167)	< 0.001	2.26	0.002	1.90
C29H11orf10	chromosome 29 open reading frame (TMEM258)	< 0.001	2.00	< 0.001	1.92
C3AR1	complement component 3a receptor 1	0.002	-2.44	0.022	-1.75
C3H1orf113	SH3 domain containing 21 (SH3D21)	< 0.001	2.70	0.001	1.79
C7H19orf10	chromosome 7 open reading frame	< 0.001	3.30	< 0.001	2.90
C9H6orf163	chromosome 9 open reading frame,	0.023	-1.55	0.003	-1.95
C9HXorf21	chromosome 9 open reading frame	0.010	-1.78	0.036	-1.54
CA13	carbonic anhydrase XIII	0.035	-1.62	0.001	-2.66
CA2	carbonic anhydrase II	0.002	3.89	< 0.001	7.68
CALCOCO1	calcium binding and coiled-coil domain 1	< 0.001	-1.66	< 0.001	-1.85
CALML4	calmodulin-like 4	< 0.001	1.67	< 0.001	1.56
CALR	calreticulin	< 0.001	2.40	< 0.001	2.04
CALU	calumenin	< 0.001	2.54	< 0.001	2.02
CAPN14	calpain 14	0.002	3.41	0.032	1.99
CASC5	cancer susceptibility candidate 5	0.004	2.48	0.004	2.43
CAV1	caveolin 1, caveolae protein, 22kDa	0.005	-3.44	0.003	-4.14
CBX7	chromobox homolog 7	< 0.001	-2.01	< 0.001	-2.24
CCDC122	coiled-coil domain containing 122	0.002	-2.23	0.011	-1.79
CCDC134	coiled-coil domain containing 134	< 0.001	3.63	< 0.001	2.53
CCDC149	coiled-coil domain containing 149	0.001	1.79	0.002	1.69
CCDC28A	coiled-coil domain containing 28A	< 0.001	-1.90	< 0.001	-1.70
CCDC56	coiled-coil domain containing 56 (COA3)	0.009	1.53	0.005	1.62
CCDC85A	coiled-coil domain containing 85A	0.006	1.80	0.001	2.14
CCDC99	coiled-coil domain containing 99 (SPDL1)	0.001	2.22	0.002	2.16
CCNA2	cyclin A2	0.001	1.88	0.008	1.52
CCND1	cyclin D1	0.018	-1.99	0.015	-2.06

CCNE1	cyclin E1	< 0.001	2.69	0.004	1.63
CCNYL1	cyclin Y-like 1	< 0.001	1.84	< 0.001	1.65
CCR8	chemokine (C-C motif) receptor 8	< 0.001	-1.92	< 0.001	-2.24
CCT3	chaperonin containing TCP1, subunit 3 (gamma)	< 0.001	1.62	0.001	1.54
CCT5	chaperonin containing TCP1, subunit 5 (epsilon)	< 0.001	1.68	< 0.001	1.82
CCT6A	chaperonin containing TCP1, subunit 6A (zeta 1)	< 0.001	1.76	< 0.001	1.82
CCT8	chaperonin containing TCP1, subunit 8 (theta)	< 0.001	1.55	< 0.001	1.73
CD244	CD244 molecule, natural killer cell receptor 2B4	0.004	-2.01	0.027	-1.61
CD63	CD63 molecule	< 0.001	1.63	< 0.001	1.74
CD7	CD7 molecule	0.009	-1.85	0.020	-1.69
CDA	cytidine deaminase	0.004	-1.72	0.002	-1.86
CDC20	cell division cycle 20 homolog (S. cerevisiae)	0.004	2.66	0.047	1.78
CDC20B	cell division cycle 20 homolog B (S. cerevisiae)	< 0.001	17.49	0.001	6.35
CDC25C	cell division cycle 25 homolog C (S. pombe)	0.001	1.80	0.002	1.72
CDC6	cell division cycle 6 homolog (S. cerevisiae)	0.001	4.53	0.004	3.25
CDCA2	cell division cycle associated 2	0.001	2.49	0.002	2.21
CDK1	cyclin-dependent kinase 1	0.003	1.88	0.002	1.98
CDK2AP2	cyclin-dependent kinase 2 associated protein 2	< 0.001	3.10	0.001	1.88
CDKN3	cyclin-dependent kinase inhibitor 3	0.002	3.61	0.002	3.62
CEACAM8	carcinoembryonic antigen-related cell adhesion molecule 1 (biliary glycoprotein)	0.017	-1.86	0.004	2.26
CENPK	centromere protein K	< 0.001	3.85	< 0.001	4.13
CENPN	centromere protein N	< 0.001	3.14	< 0.001	2.54
CENPP	centromere protein P	0.004	1.88	0.028	1.53
CENPW	centromere protein W	0.001	2.98	0.005	3.13
CEP55	centrosomal protein 55kDa	0.002	2.36	0.016	1.74
CGN	cingulin	< 0.001	-1.58	< 0.001	-1.58
CHAC1	ChaC, cation transport regulator homolog 1 (E. coli)	< 0.001	1.76	< 0.001	1.89
CHAC2	ChaC, cation transport regulator homolog 2 (E. coli)	< 0.001	1.68	< 0.001	1.64
CHEK1	CHK1 checkpoint homolog (S. pombe)	0.011	2.20	0.020	2.00
CHID1	chitinase domain containing 1	< 0.001	1.85	< 0.001	2.00
CHODL	chondrolectin	0.003	2.94	0.005	2.77
CHPF	chondroitin polymerizing factor	< 0.001	1.66	< 0.001	1.70
CHRDL1	chordin-like 1	0.002	-3.31	0.024	-2.14
CHST8	carbohydrate (N-acetylgalactosamine 4-0) sulfotransferase 8	0.005	2.35	0.001	3.30
CHSY1	chondroitin sulfate synthase 1	< 0.001	1.53	< 0.001	1.72
CHSY3	chondroitin sulfate synthase 3	0.002	1.90	0.001	2.02
CIRBP	cold inducible RNA binding protein	< 0.001	-1.91	< 0.001	-1.76
CISD2	CDGSH iron sulfur domain 2	< 0.001	2.12	< 0.001	1.94

CKAP2	cytoskeleton associated protein 2	0.013	1.82	0.004	2.11
CKAP4	cytoskeleton-associated protein 4	< 0.001	3.60	< 0.001	2.41
CKS2	CDC28 protein kinase regulatory subunit 2	< 0.001	2.31	< 0.001	1.87
CLDN11	claudin 11	0.009	2.59	0.018	2.29
CLDN16	claudin 16	0.005	-1.81	0.006	-1.78
CLDN8	claudin 8	0.017	-1.58	0.001	-2.29
CLEC2D	C-type lectin domain family 2, member D	0.008	-2.88	0.016	-2.52
CLIC1	chloride intracellular channel 1	< 0.001	1.57	< 0.001	1.84
CLIC5	chloride intracellular channel 5	0.042	-1.74	< 0.001	-3.90
CLIC6	chloride intracellular channel 6	0.011	-2.06	0.005	-2.27
CLK1	CDC-like kinase 1	< 0.001	-1.67	< 0.001	-1.54
CLK4	CDC-like kinase 4	< 0.001	-1.78	< 0.001	-1.65
CLPH	calcium-binding protein, spermatid-specific 1	< 0.001	6.61	0.003	2.74
CLPTM1L	CLPTM1-like	< 0.001	2.21	< 0.001	1.82
CNIH	cornichon homolog (Drosophila)	< 0.001	1.62	< 0.001	1.61
CNNM3	cyclin M3	< 0.001	-1.53	< 0.001	-1.63
CNPY2	canopy 2 homolog (zebrafish)	< 0.001	2.12	< 0.001	2.19
CNPY3	canopy 3 homolog (zebrafish)	< 0.001	1.73	0.001	1.60
CNTFR	ciliary neurotrophic factor receptor	< 0.001	3.27	0.020	1.57
CNTNAP3	contactin associated protein-like 3	0.023	-2.98	0.007	-4.09
COPE	coatomer protein complex, subunit epsilon	< 0.001	1.81	< 0.001	1.64
COX5A	cytochrome c oxidase subunit Va	0.001	1.62	0.002	1.50
COX5B	cytochrome c oxidase subunit Vb	0.006	1.76	0.003	1.91
COX7B	cytochrome c oxidase subunit VIIb	< 0.001	1.63	< 0.001	1.64
CRABP2	cellular retinoic acid binding protein 2	0.004	2.27	0.049	1.60
CRELD1	cysteine-rich with EGF-like domains 1	< 0.001	2.51	< 0.001	2.37
CRELD2	cysteine-rich with EGF-like domains 2	< 0.001	9.98	< 0.001	7.69
CRYBG3	uncharacterized LOC516526	< 0.001	-2.14	< 0.001	-1.94
CS	citrate synthase	< 0.001	1.87	0.001	1.53
CSF1	colony stimulating factor 1 (macrophage)	0.008	-1.98	0.019	-1.76
CSF1R	colony stimulating factor 1 receptor	0.004	-1.75	0.005	-1.71
CTH	cystathionase (cystathionine gamma-lyase)	0.001	1.99	< 0.001	2.30
CTSF	cathepsin F	< 0.001	-1.63	< 0.001	-1.78
CWH43	cell wall biogenesis 43 C-terminal homolog (S. cerevisiae)	0.034	-2.24	0.001	-5.09
CYP2B6	cytochrome P450 subfamily 2B	0.002	-1.80	< 0.001	-2.05
CYP2J2	cytochrome P450, family 2, subfamily J, polypeptide 2	0.004	-1.57	0.003	-1.61
CYP2R1	cytochrome P450, family 2, subfamily R, polypeptide 1	0.002	1.52	0.001	1.62
CYP4V2	cytochrome P450, family 4, subfamily V, polypeptide 2	0.002	-1.73	0.002	-1.72

CYTIP	cytohesin 1 interacting protein	0.008	-1.77	0.014	-1.68
DAPK2	death-associated protein kinase 2	0.005	2.13	0.002	2.48
DBF4	DBF4 homolog (S. cerevisiae)	0.001	1.54	< 0.001	1.83
DBI	diazepam binding inhibitor (GABA receptor modulator, acyl-CoA binding protein)	< 0.001	2.68	< 0.001	1.67
DBP	D site of albumin promoter (albumin D-box) binding protein	< 0.001	-2.07	< 0.001	-1.84
DCLRE1B	DNA cross-link repair 1B	< 0.001	3.03	< 0.001	3.43
DCTPP1	dCTP pyrophosphatase 1	0.001	2.31	0.012	1.71
DDIT3	DNA-damage-inducible transcript 3	< 0.001	2.02	< 0.001	2.08
DDOST	dolichyl-diphosphooligosaccharide--protein glycosyltransferase	< 0.001	1.84	< 0.001	1.67
DDX10	DEAD (Asp-Glu-Ala-Asp) box polypeptide 10	< 0.001	1.67	< 0.001	1.59
DDX21	DEAD (Asp-Glu-Ala-Asp) box polypeptide 21	< 0.001	1.50	< 0.001	1.52
DDX58	DEAD (Asp-Glu-Ala-Asp) box polypeptide 58	0.003	-1.62	< 0.001	-2.36
DENR	density-regulated protein	< 0.001	1.51	< 0.001	1.76
DEPDC1	DEP domain containing 1	0.006	2.58	0.004	2.77
DERL1	derlin 1	< 0.001	1.83	< 0.001	1.76
DERL2	Der1-like domain family, member 2	< 0.001	1.89	< 0.001	1.66
DERL3	Der1-like domain family, member 3	< 0.001	2.69	0.009	1.60
DGKA	diacylglycerol kinase, alpha 80kDa	0.001	-1.75	0.003	-1.53
DHDPSL	4-hydroxy-2-oxoglutarate aldolase 1	< 0.001	1.75	0.001	1.52
DHRS1	dehydrogenase/reductase (SDR family) member 1	0.001	1.87	0.004	1.60
DIAPH3	diaphanous-related formin 3	0.003	1.92	0.005	1.81
DLGAP5	discs, large (Drosophila) homolog-associated protein 5	0.001	3.15	0.012	1.98
DMD	dystrophin	< 0.001	-1.81	< 0.001	-2.08
DNA2	DNA replication helicase 2 homolog (yeast)	0.001	2.14	0.004	1.80
DNAH5	dynein, axonemal, heavy chain 5	< 0.001	-1.57	< 0.001	-1.63
DNAJB11	DnaJ (Hsp40) homolog, subfamily B, member 11	< 0.001	6.92	< 0.001	5.89
DNAJB5	DnaJ (Hsp40) homolog, subfamily B, member 5	< 0.001	2.36	< 0.001	1.78
DNAJB9	DnaJ (Hsp40) homolog, subfamily B, member 9	< 0.001	2.67	< 0.001	2.41
DNAJC10	DnaJ (Hsp40) homolog, subfamily C, member 10	< 0.001	1.57	< 0.001	1.68
DNAJC3	DnaJ (Hsp40) homolog, subfamily C, member 3	< 0.001	2.68	< 0.001	2.18
DNASE1L3	deoxyribonuclease I-like 3	0.011	-2.21	0.019	-2.02
DOCK10	dedicator of cytokinesis 10	0.006	-2.59	0.022	-2.05
DPAGT1	dolichyl-phosphate (UDP-N-acetylglucosamine) N-acetylglucosaminephosphotransferase 1 (GlcNAc-1-P transferase)	< 0.001	2.08	< 0.001	1.52
DPP3	dipeptidyl-peptidase 3	< 0.001	2.43	< 0.001	2.05
DUSP1	dual specificity phosphatase 1	0.022	-1.65	0.020	-1.68
DUSP10	dual specificity phosphatase 10	0.003	-1.51	0.001	-1.60
E2F8	E2F transcription factor 8	< 0.001	3.42	0.001	2.17

EBAG9	estrogen receptor binding site associated, antigen, 9	< 0.001	1.69	< 0.001	1.59
EBNA1BP2	EBNA1 binding protein 2	< 0.001	1.55	< 0.001	1.65
ECT2	epithelial cell transforming sequence 2 oncogene	0.001	2.04	0.007	1.63
EDEM1	ER degradation enhancer, mannosidase alpha-like 1	< 0.001	3.93	< 0.001	2.64
EDEM2	ER degradation enhancer, mannosidase alpha-like 2	< 0.001	1.80	< 0.001	1.60
EGR1	early growth response 1	0.005	-9.13	0.019	-5.35
EHD4	EH-domain containing 4	0.001	1.88	0.001	1.85
EIF2AK3	eukaryotic translation initiation factor 2-alpha kinase 3	< 0.001	1.97	< 0.001	1.53
EIF2S2	eukaryotic translation initiation factor 2, subunit 2 beta, 38kDa	< 0.001	1.87	< 0.001	1.71
EIF4A1	eukaryotic translation initiation factor 4A1	< 0.001	1.51	< 0.001	1.67
EIF5A	eukaryotic translation initiation factor 5A	< 0.001	1.86	< 0.001	1.75
ELL3	elongation factor RNA polymerase II-like 3	0.004	-1.59	0.001	-1.79
ELMO1	engulfment and cell motility 1	0.025	-1.60	0.003	-2.03
ELOVL1	ELOVL fatty acid elongase 1	< 0.001	1.61	< 0.001	1.65
EMILIN1	elastin microfibril interfacer 1	< 0.001	2.49	0.001	1.67
EMP3	epithelial membrane protein 3	0.014	1.71	0.018	1.66
ENTPD7	ectonucleoside triphosphate diphosphohydrolase 7-like	< 0.001	1.76	< 0.001	1.60
EPB41L4B	erythrocyte membrane protein band 4.1 like 4B	< 0.001	-1.53	< 0.001	-1.54
EPHA4	EPH receptor A4	0.004	2.37	< 0.001	3.66
EPHB1	EPH receptor B1	0.001	2.09	< 0.001	2.42
ERCC6L	excision repair cross-complementing rodent repair deficiency, complementation group 6-like	< 0.001	2.17	0.002	1.59
ERH	enhancer of rudimentary homolog (Drosophila)	0.001	2.02	0.004	1.80
ERO1L	ERO1-like (S. cerevisiae)	< 0.001	1.53	< 0.001	1.52
ERO1LB	ERO1-like beta (S. cerevisiae)	< 0.001	1.87	0.001	1.79
ERP29	endoplasmic reticulum protein 29	< 0.001	2.09	< 0.001	1.83
ERP44	endoplasmic reticulum protein 44	< 0.001	1.61	< 0.001	1.71
ESCO2	establishment of cohesion 1 homolog 2 (S. cerevisiae)	< 0.001	2.91	0.001	2.05
ESR2	estrogen receptor 2 (ER beta)	0.006	-1.98	0.001	-2.60
EVI2A	ecotropic viral integration site 2A	0.020	-2.00	0.012	-2.16
EVI2B	ecotropic viral integration site 2B	0.002	-2.99	0.011	-2.30
EXO1	exonuclease 1	0.001	3.61	0.008	2.49
EXOSC4	exosome component 4	0.001	1.59	0.002	1.52
EXT1	exostosin 1	0.010	1.66	< 0.001	2.38
EZH1	enhancer of zeste homolog 1 (Drosophila)	< 0.001	-1.77	< 0.001	-1.67
EZH2	enhancer of zeste homolog 2 (Drosophila)	0.001	2.39	0.002	2.22
F3	coagulation factor III	0.002	2.80	0.020	1.98

FAM174B	family with sequence similarity 174, member B	< 0.001	2.41	0.005	1.52
FAM33A	spindle and kinetochore associated complex subunit 2 (SKA2)	< 0.001	1.68	< 0.001	1.63
FAM54A	family with sequence similarity 54, member A	< 0.001	3.04	< 0.001	2.94
FAM96B	family with sequence similarity 96, member B	< 0.001	1.68	< 0.001	1.75
FANCD2	Fanconi anemia, complementation group D2	< 0.001	2.50	< 0.001	2.47
FASN	fatty acid synthase	< 0.001	3.63	< 0.001	2.04
FBL	fibrillarin	< 0.001	1.99	< 0.001	1.78
FBN1	fibrillin 1	0.028	-1.52	0.002	-2.02
FBP1	fructose-1,6-bisphosphatase 1	0.001	2.83	< 0.001	4.19
FBXL20	F-box and leucine-rich repeat protein 20	< 0.001	-1.73	< 0.001	-1.58
FBXL3	F-box and leucine-rich repeat protein 3	< 0.001	-1.79	< 0.001	-1.52
FBXO32	F-box protein 32	0.002	-1.73	0.001	-1.93
FBXO5	F-box protein 5	0.001	2.14	0.014	1.54
FCGR3A	Fc fragment of IgG, low affinity IIIa, receptor (CD16a)	0.003	-1.89	0.020	-1.57
FDPS	farnesyl diphosphate synthase	< 0.001	3.41	< 0.001	2.01
FGFBP1	fibroblast growth factor binding protein 1	< 0.001	3.82	< 0.001	4.20
FGL2	fibrinogen-like 2	0.001	-1.91	0.003	-1.75
FICD	FIC domain containing	< 0.001	2.85	< 0.001	2.20
FKBP10	FK506 binding protein 10, 65 kDa	< 0.001	2.47	0.001	1.88
FKBP11	FK506 binding protein 11, 19 kDa	< 0.001	4.86	< 0.001	3.51
FKBP2	FK506 binding protein 2, 13kDa	< 0.001	2.76	0.001	2.17
FKBP5	FK506 binding protein 5	0.004	-2.04	0.001	-2.53
FKBP7	FK506 binding protein 7	< 0.001	2.93	< 0.001	2.00
FMOD	fibromodulin	0.016	2.90	0.007	3.56
FNBP1	formin binding protein 1	0.001	-1.69	< 0.001	-1.87
FOS	FBJ murine osteosarcoma viral oncogene homolog	0.002	-8.55	0.012	-4.54
FOXO4	forkhead box O4	< 0.001	-2.11	< 0.001	-2.03
FOXRED2	FAD-dependent oxidoreductase domain containing 2	0.006	2.70	0.003	3.03
FRYL	FRY-like	0.002	-1.69	< 0.001	-2.75
GABARAPL 1	GABA(A) receptor-associated protein like 1	< 0.001	-1.60	< 0.001	-1.55
GALE	UDP-galactose-4-epimerase	< 0.001	3.09	0.001	1.79
GANC	glucosidase, alpha; neutral C	0.002	-1.97	0.006	-1.73
GARS	glycyl-tRNA synthetase	< 0.001	2.07	< 0.001	1.70
GEM	GTP binding protein overexpressed in skeletal muscle	0.041	-1.61	0.009	-1.95
GFAP	glial fibrillary acidic protein	0.007	2.10	< 0.001	8.09
GFPT1	glutamine--fructose-6-phosphate transaminase 1	< 0.001	1.70	0.001	1.51
GGH	gamma-glutamyl hydrolase (conjugase,	0.006	1.97	< 0.001	3.05

	folylpolygammaglutamyl hydrolase)				
GIMAP8	GTPase, IMAP family member 8	0.007	-1.86	0.016	-1.70
GJA8	gap junction protein, alpha 8, 50kDa	0.002	1.72	0.011	1.50
GMDS	GDP-mannose 4,6-dehydratase	0.001	1.73	0.002	1.57
GMPPA	GDP-mannose pyrophosphorylase A	< 0.001	2.12	< 0.001	1.55
GNE	glucosamine (UDP-N-acetyl)-2-epimerase/N-acetylmannosamine kinase	< 0.001	2.37	< 0.001	1.70
GNPNAT1	glucosamine-phosphate N-acetyltransferase 1	< 0.001	2.32	< 0.001	2.05
GOLT1B	golgi transport 1B	< 0.001	1.63	< 0.001	1.54
GOT1	glutamic-oxaloacetic transaminase 1, soluble (aspartate aminotransferase 1)	< 0.001	1.68	< 0.001	1.57
GPR137C	G protein-coupled receptor 137C	< 0.001	2.30	< 0.001	2.26
GPR174	G protein-coupled receptor 174	0.003	-5.15	0.020	-3.04
GPR183	G protein-coupled receptor 183	0.008	-1.93	0.021	-1.71
GPR34	G protein-coupled receptor 34	0.005	-2.29	0.014	-1.94
GRAMD1C	GRAM domain containing 1C	0.003	-1.53	0.002	-1.57
GRB7	growth factor receptor-bound protein 7	0.003	-1.54	< 0.001	-2.12
GREB1	growth regulation by estrogen in breast cancer 1	< 0.001	2.41	< 0.001	2.32
GRID1	glutamate receptor, ionotropic, delta 1	0.002	-1.71	0.002	-1.75
GSR	glutathione reductase	< 0.001	2.47	< 0.001	2.03
GSTM1	glutathione S-transferase M1	0.002	2.42	< 0.001	3.98
GTSE1	G-2 and S-phase expressed 1	< 0.001	2.12	< 0.001	1.90
H4	histone H4	< 0.001	6.30	< 0.001	4.86
HBP1	HMG-box transcription factor 1	< 0.001	-1.72	0.001	-1.67
HEATR4	HEAT repeat containing 4	0.001	-1.60	0.001	-1.67
HECW1	HECT, C2 and WW domain containing E3 ubiquitin protein ligase 1	< 0.001	-2.42	< 0.001	-2.82
HELLS	helicase, lymphoid-specific	0.004	2.43	0.001	3.01
HERC5	hect domain and RLD 5	0.001	-1.86	0.005	-1.60
HERPUD1	homocysteine-inducible, endoplasmic reticulum stress-inducible, ubiquitin-like domain member 1	< 0.001	2.31	< 0.001	1.79
HIST1H1B	histone cluster 1, H1b	0.014	1.73	0.006	1.94
HIST1H1D	histone cluster 1, H1d	0.003	-1.96	0.003	-2.02
HIST1H1E	histone cluster 1, H1e	0.005	-2.50	< 0.001	-1.63
HIST1H2BB	histone cluster 1, H2bb	< 0.001	3.25	0.001	2.10
HJURP	Holliday junction recognition protein	0.005	1.99	0.011	1.80
HM13	histocompatibility (minor) 13	< 0.001	1.66	< 0.001	1.52
HMBS	hydroxymethylbilane synthase	< 0.001	1.93	< 0.001	1.74
HMGCR	3-hydroxy-3-methylglutaryl-CoA reductase	< 0.001	2.70	< 0.001	1.56
HMGCS1	HMGCS1 protein-like	< 0.001	4.96	0.001	1.79
HN1	hematological and neurological expressed 1	< 0.001	2.12	< 0.001	2.21
HNRNPAB	heterogeneous nuclear ribonucleoprotein A/B	0.001	1.53	< 0.001	1.59

HOMER2	homer homolog 2 (Drosophila)	< 0.001	1.95	< 0.001	2.25
HSD17B10	hydroxysteroid (17-beta) dehydrogenase 10	< 0.001	2.01	< 0.001	1.88
HSP90B1	heat shock protein 90kDa beta (Grp94), member 1	< 0.001	1.91	< 0.001	2.02
HSPA5	heat shock 70kDa protein 5 (glucose-regulated protein, 78kDa)	< 0.001	2.54	< 0.001	2.59
HTR2A	5-hydroxytryptamine (serotonin) receptor 2A	0.015	-1.59	0.012	1.63
IDI1	isopentenyl-diphosphate delta isomerase 1	< 0.001	2.61	0.002	1.54
IER2	immediate early response 2	0.009	-2.23	0.031	-1.84
IFI6	interferon, alpha-inducible protein 6	0.017	-1.74	0.017	-1.74
IGF1	insulin-like growth factor 1 (somatomedin C)	0.026	1.54	0.015	1.63
IGF2BP2	insulin-like growth factor 2 mRNA binding protein 2	0.031	-1.56	< 0.001	-2.77
IGFBP6	insulin-like growth factor binding protein 6	0.014	1.97	0.002	2.64
IKZF3	IKAROS family zinc finger 3 (Aiolos)	0.007	-2.67	0.012	-2.42
IL1RAPL2	interleukin 1 receptor accessory protein-like 2	0.016	-1.71	0.010	-1.80
IMPAD1	inositol monophosphatase domain containing 1	< 0.001	1.55	< 0.001	1.53
INO80C	INO80 complex subunit C	< 0.001	1.70	< 0.001	1.84
INSIG1	insulin induced gene 1	< 0.001	6.21	0.001	1.65
INSL5	insulin-like 5	0.015	-1.53	0.011	-1.58
INSR	insulin receptor	< 0.001	-2.07	< 0.001	-1.99
IPCEF1	interaction protein for cytohesin exchange factors 1	0.011	-1.98	0.011	-1.99
IPO4	importin 4	< 0.001	1.65	< 0.001	1.64
IRF1	interferon regulatory factor 1	0.012	-1.70	0.013	-1.68
IRF2BP2	interferon regulatory factor 2 binding protein 2	< 0.001	-1.59	< 0.001	-1.58
IRF9	interferon regulatory factor 9	0.002	-1.72	< 0.001	-2.28
ITPR3	inositol 1,4,5-trisphosphate receptor, type 3	< 0.001	1.91	< 0.001	2.59
KCNB1	potassium voltage-gated channel, Shab-related subfamily, member 1	0.011	-1.78	0.025	-1.61
KCNE3	potassium voltage-gated channel, Isk-related family, member 3	0.041	1.74	0.004	2.51
KCNMB2	potassium large conductance calcium-activated channel, subfamily M, beta member 2	0.025	-1.61	0.013	-1.74
KCNN2	potassium intermediate/small conductance calcium-activated channel, subfamily N, member 2	0.016	-1.77	0.003	-2.19
KIAA0141	KIAA0141 ortholog	< 0.001	1.94	0.002	1.58
KIAA1549	KIAA1549 ortholog	0.002	-1.62	0.001	-1.66
KIF11	kinesin family member 11	0.004	2.89	0.039	1.93
KIF15	kinesin family member 15	< 0.001	2.54	0.007	1.71
KIF20A	kinesin family member 20A	0.004	2.17	0.040	1.61
KIF4A	kinesin family member 4A	< 0.001	2.75	0.001	2.55
KLHL24	kelch-like 24 (Drosophila)	< 0.001	-2.27	< 0.001	-2.09

KLRB1	killer cell lectin-like receptor subfamily B, member 1	0.001	-1.67	< 0.001	-1.94
KLRC1	killer cell lectin-like receptor subfamily C, member 1	0.003	-2.47	0.020	-1.86
KLRD1	killer cell lectin-like receptor subfamily D, member 1	0.001	-2.76	0.002	-2.64
KLRK1	killer cell lectin-like receptor subfamily K, member 1	0.003	-4.09	0.016	-2.84
KNTC1	kinetochore associated 1	0.008	1.74	0.002	2.10
KPNA2	karyopherin alpha 2 (RAG cohort 1, importin alpha 1)	< 0.001	2.15	< 0.001	2.28
KRT23	keratin 23 (histone deacetylase inducible)	0.004	8.40	< 0.001	22.57
KRT5	keratin 5	0.007	1.57	< 0.001	2.23
KRT8	keratin 8	< 0.001	1.77	0.001	1.70
KRTCAP2	keratinocyte associated protein 2	< 0.001	1.76	< 0.001	1.53
LAPTM5	lysosomal protein transmembrane 5	0.003	-3.21	0.011	-2.54
LASS6	ceramide synthase 6	0.003	1.69	0.001	1.87
LDHA	lactate dehydrogenase A	< 0.001	2.22	< 0.001	2.34
LENG8	leukocyte receptor cluster (LRC) member 8	< 0.001	-1.77	0.001	-1.63
LEPREL4	leprecan-like 4	< 0.001	2.68	< 0.001	2.37
LGALS1	lectin, galactoside-binding, soluble, 1	0.033	1.75	0.002	2.64
LGALS9	lectin, galactoside-binding, soluble, 9	0.003	-1.82	0.001	-2.21
LHFPL3	lipoma HMGIC fusion partner-like 3	< 0.001	5.07	< 0.001	4.47
LIPG	endothelial lipase-like	< 0.001	4.34	0.001	3.02
LMAN2	lectin, mannose-binding 2	< 0.001	1.69	< 0.001	1.67
LOC100137 989	mucin-5AC-like	0.010	2.49	0.001	4.10
LOC100138 905	zinc finger protein 347-like	< 0.001	-1.63	0.001	-1.52
LOC100139 208	SP140 nuclear body protein-like	< 0.001	-1.68	< 0.001	-1.77
LOC100140 338	SP100 nuclear antigen	< 0.001	-2.21	< 0.001	-2.17
LOC100141 246	V-set and transmembrane domain containing 5	< 0.001	-2.17	< 0.001	-2.99
LOC100295 228	chromosome 19 open reading frame	< 0.001	1.66	< 0.001	1.55
LOC100295 639	FXYD domain containing ion transport regulator 4	< 0.001	2.54	< 0.001	2.43
LOC100296 605	adhesion molecule with Ig-like domain 3	< 0.001	3.95	< 0.001	2.64
LOC100296 742	beta-defensin 128-like	0.034	4.48	0.018	5.61
LOC100296 782	uncharacterized LOC100296782	< 0.001	-1.65	< 0.001	-1.54
LOC100297 225	uncharacterized LOC100297225	0.008	-1.72	0.001	-2.13
LOC100297	C-type lectin domain family 2 member G-like	0.003	-3.78	0.026	-2.39

676					
LOC100301 247	cyclin O	0.001	2.28	0.007	1.73
LOC100301 340	multiciliate cell differentiation 1	0.002	1.88	0.015	1.52
LOC100335 668	mitochondrial import inner membrane translocase subunit Tim9 pseudogene	0.017	-2.36	0.001	-4.94
LOC100336 476	colony stimulating factor 2 receptor, alpha, low-affinity (granulocyte-macrophage)-like	0.001	-1.60	< 0.001	-2.47
LOC100337 041	sphingosine-1-phosphate phosphatase 2-like	0.019	1.72	0.001	-2.50
LOC100337 183	contactin associated protein-like 3-like	0.008	-3.71	0.003	-4.75
LOC100337 409	disabled homolog 2 interacting protein-like	0.001	1.66	< 0.001	2.05
LOC504200	olfactory receptor, family 5, subfamily AN, member 1-like	0.010	-1.86	0.012	-1.82
LOC504599	histone H3.2	< 0.001	2.46	0.002	1.74
LOC506181	zinc finger protein 709-like	< 0.001	1.64	< 0.001	1.85
LOC507661	MIS18 binding protein 1	0.001	2.49	0.009	1.76
LOC508455	estradiol 17-beta-dehydrogenase 12-like	< 0.001	-1.80	< 0.001	-1.84
LOC511138	chromosome 29 open reading frame, human C11orf80	0.007	-1.81	0.002	-2.17
LOC513508	acyl-CoA dehydrogenase family, member 10-like	< 0.001	-1.66	< 0.001	-1.65
LOC513822	RIKEN cDNA 4832428D23-like	0.012	-1.64	0.007	-1.76
LOC515736	uncharacterized LOC515736	0.001	-2.16	< 0.001	-3.12
LOC523019	Sp4 transcription factor	0.001	-1.57	.	-1.53
LOC527414	olfactory receptor, family 2, subfamily A, member 12-like	0.001	1.55	0.001	1.59
LOC527415	olfactory receptor, family 2, subfamily A, member 5-like	0.023	1.55	< 0.001	2.45
LOC528262	intestinal-type alkaline phosphatase-like	0.027	-1.54	0.003	-1.98
LOC531747	adenylate cyclase type 10-like	< 0.001	-1.77	0.001	-1.57
LOC533818	rCG28728-like	0.027	1.52	0.002	1.96
LOC538402	family with sequence similarity 81, member A	< 0.001	1.55	0.001	1.53
LOC540169	GLTSCR1-like	< 0.001	-1.73	< 0.001	-1.55
LOC540234	family with sequence similarity 46, member A	0.015	1.74	0.013	1.77
LOC540812	armadillo repeat containing 12	0.004	-1.63	0.001	-1.98
LOC540918	family with sequence similarity 13, member C	0.008	-1.80	< 0.001	-3.84
LOC614351	DNA replication complex GINS protein PSF2-like	0.003	2.01	0.003	2.01
LOC614734	transmembrane protein 241	< 0.001	1.98	< 0.001	1.69
LOC615622	spindle and kinetochore-associated protein 2 pseudogene	0.002	1.60	0.001	1.65
LOC615685	maturin, neural progenitor differentiation regulator homolog (Xenopus)	0.006	-1.52	< 0.001	-1.91
LOC615968	equatorin, sperm acrosome associated	< 0.001	-1.85	< 0.001	-1.90

LOC616903	protein CutA homolog-like	< 0.001	-1.52	< 0.001	-1.64
LOC616908	family with sequence similarity 190, member A	< 0.001	-1.62	< 0.001	-1.98
LOC617219	lysozyme C, tracheal isozyme-like	0.040	1.69	0.024	1.82
LOC617981	family with sequence similarity 55, member C-like	0.047	-1.58	< 0.001	-4.92
LOC618247	ZMYM6 neighbor	< 0.001	2.14	< 0.001	2.12
LOC618409	interferon regulatory factor 4-like	< 0.001	-2.11	0.001	-1.62
LOC619083	olfactory receptor, family 5, subfamily AN, member 1-like	< 0.001	-2.41	0.002	-1.96
LOC768255	GTPase, IMAP family member 4-like	0.001	-3.64	0.011	-2.43
LOC777601	uncharacterized LOC777601	< 0.001	-4.37	0.006	-2.57
LOC782545	L-amino-acid oxidase-like	0.017	2.08	< 0.001	4.70
LOC783459	chromosome 14 open reading frame	0.002	1.74	< 0.001	2.04
LOC783553	chromosome 5 open reading frame	< 0.001	5.22	0.006	2.85
LOC783761	enhancer of rudimentary homolog pseudogene	0.001	1.53	< 0.001	1.69
LOC784440	rab11 family-interacting protein 1-like	< 0.001	-1.52	< 0.001	-1.64
LOC784451	C-type lectin domain family 2 member D11-like	0.019	-1.90	0.008	-2.16
LOC785366	embigin	< 0.001	-1.86	< 0.001	-1.79
LOC785866	olfactory receptor, family 5, subfamily AN, member 1-like	0.003	-2.69	0.001	-3.46
LOC785899	olfactory receptor, family 5, subfamily AN, member 1-like	0.006	-2.51	0.003	-2.76
LOC787812	uncharacterized LOC787812	0.001	-1.54	0.001	-1.52
LOC788334	apolipoprotein L3-like	0.021	-1.60	< 0.001	-2.63
LOX	lysyl oxidase	0.003	3.09	0.037	1.94
LPAR3	lysophosphatidic acid receptor 3	0.001	2.30	0.001	2.24
LPL	lipoprotein lipase	0.006	3.09	< 0.001	9.40
LPLUNC1	BPI fold containing family B, member 1 (BPIFB1)	0.001	-2.03	< 0.001	-3.85
LRF	chromosome 20 open reading frame	< 0.001	-2.24	0.001	-1.71
LRP2BP	LRP2 binding protein	0.001	-1.94	0.001	-2.01
LRP8	low density lipoprotein receptor-related protein 8, apolipoprotein e receptor	< 0.001	2.11	< 0.001	1.65
LRRC4C	leucine rich repeat containing 4C	< 0.001	-2.77	0.005	-1.72
LRRC59	leucine rich repeat containing 59	< 0.001	2.27	0.001	1.51
LSM4	LSM4 homolog, U6 small nuclear RNA associated (S. cerevisiae)	0.002	1.90	0.001	2.06
LSM7	LSM7 homolog, U6 small nuclear RNA associated (S. cerevisiae)	< 0.001	1.69	< 0.001	1.77
LSS	lanosterol synthase (2,3-oxidosqualene-lanosterol cyclase)	< 0.001	3.14	< 0.001	1.73
LXN	latexin	0.001	2.75	< 0.001	3.41
LY9	lymphocyte antigen 9	0.005	-2.61	0.024	-1.99
LYSMD3	LysM, putative peptidoglycan-binding, domain containing 3	< 0.001	1.62	< 0.001	1.57
MAGED1	melanoma antigen family D, 1	< 0.001	1.90	< 0.001	1.74

MAK16	MAK16 homolog (S. cerevisiae)	< 0.001	1.62	< 0.001	1.72
MAMSTR	MEF2 activating motif and SAP domain containing transcriptional regulator	0.001	1.72	0.001	1.68
MAN1A1	mannosidase, alpha, class 1A, member 1	0.022	1.55	< 0.001	2.39
MANF	mesencephalic astrocyte-derived neurotrophic factor	< 0.001	6.90	< 0.001	5.85
MAP1LC3C	microtubule-associated protein 1 light chain 3 gamma	< 0.001	2.19	< 0.001	1.81
MAP3K12	mitogen-activated protein kinase kinase kinase 12	< 0.001	1.62	< 0.001	1.77
MAP3K15	mitogen-activated protein kinase kinase kinase 15	< 0.001	-2.81	< 0.001	-2.41
MAP3K5	mitogen-activated protein kinase kinase kinase 5	< 0.001	-1.82	0.002	-1.60
MARCKSL1	MARCKS-like 1	0.013	1.52	< 0.001	2.40
MARS	methionyl-tRNA synthetase	< 0.001	1.92	< 0.001	1.80
MASTL	microtubule associated serine/threonine kinase-like	0.001	2.95	0.004	2.53
MCM10	minichromosome maintenance complex component 10	0.003	2.13	0.004	2.02
MCM2	minichromosome maintenance complex component 2	0.002	2.07	0.006	1.82
MCM3	minichromosome maintenance complex component 3	0.018	1.77	0.026	1.68
MCM6	minichromosome maintenance complex component 6	0.008	1.74	0.005	1.82
MDH1	malate dehydrogenase 1, NAD (soluble)	< 0.001	2.14	< 0.001	1.98
MEGF10	multiple EGF-like-domains 10	0.017	-1.65	< 0.001	-4.81
MELK	maternal embryonic leucine zipper kinase	< 0.001	2.88	< 0.001	2.45
MET	met proto-oncogene (hepatocyte growth factor receptor)	0.006	-2.07	0.002	-2.44
METTL1	methyltransferase like 1	< 0.001	1.65	< 0.001	1.69
METTL12	methyltransferase like 12	0.001	1.52	< 0.001	1.84
MFSD2A	major facilitator superfamily domain containing 2A	< 0.001	6.70	0.021	1.71
MGC128424	putative small membrane protein NID67	< 0.001	1.73	< 0.001	1.79
MGC134282	chromosome 29 open reading frame, human C11orf48	0.011	1.51	0.004	1.64
MGC134574	family with sequence similarity 221, member A	0.005	-1.60	0.010	-1.51
MGC137708	uncharacterized LOC516719	< 0.001	2.10	0.001	1.81
MGC138914	uncharacterized LOC512219	0.024	-2.42	0.009	-3.01
MGC139603	uncharacterized LOC509055	< 0.001	2.16	< 0.001	1.65
MGC151592	chromosome 16 open reading frame, human C1orf95	0.016	1.53	< 0.001	2.39
MGC154956	GTPase, IMAP family member 5-like	0.002	-3.08	0.005	-2.65
MIA	melanoma inhibitory activity	< 0.001	-1.56	< 0.001	-1.71
MID1IP1	MID1 interacting protein 1 (gastrulation specific G12 homolog (zebrafish))	< 0.001	1.90	< 0.001	1.88
MIR1301	microRNA mir-1301	0.001	-1.56	< 0.001	-1.75
MIR2408	microRNA mir-2408	< 0.001	2.29	< 0.001	1.90

MIR2470	microRNA mir-2470	0.001	-1.81	0.001	-1.77
MIR2471	microRNA mir-2471	0.005	-1.81	0.007	-1.74
MIR2475	microRNA mir-2475	0.001	-1.69	0.001	-1.70
MIR2486	microRNA mir-2486	< 0.001	-1.95	< 0.001	-2.07
MIR27B	microRNA mir-27b	0.002	-2.47	< 0.001	-3.56
MIR29B-2	microRNA mir-29b-2	0.002	-2.56	0.034	-1.72
MIR29C	microRNA mir-29c	0.001	-3.29	0.003	-2.67
MIR29D	microRNA mir-29d	0.003	-2.70	0.029	-1.84
MIR449C	microRNA mir-449c	< 0.001	9.20	< 0.001	4.90
MIR95	microRNA mir-95	0.016	-1.76	< 0.001	-3.50
MIS12	MIS12, MIND kinetochore complex component, homolog (S. pombe)	< 0.001	1.99	0.001	1.63
MKI67	antigen identified by monoclonal antibody Ki-67	0.008	2.91	0.018	2.46
MLEC	malectin	0.001	1.81	0.003	1.65
MLLT3	myeloid/lymphoid or mixed-lineage leukemia (trithorax homolog, Drosophila); translocated to, 3	0.001	-1.51	< 0.001	-1.58
MMP2	matrix metallopeptidase 2 (gelatinase A, 72kDa gelatinase, 72kDa type IV collagenase)	< 0.001	1.59	< 0.001	1.87
MMP7	matrix metallopeptidase 7 (matrilysin, uterine)	0.009	-2.83	0.017	2.51
MOBKL2B	MOB kinase activator 3B	< 0.001	-1.62	< 0.001	-1.59
MOCOS	molybdenum cofactor sulfurase	0.011	-1.66	< 0.001	-2.69
MPEG1	macrophage expressed 1	0.007	-2.18	0.034	-1.73
MPV17L2	MPV17 mitochondrial membrane protein-like 2	< 0.001	2.99	< 0.001	2.12
MPZL1	myelin protein zero-like 1	0.007	1.51	< 0.001	2.14
MRPL18	mitochondrial ribosomal protein L18	0.012	1.62	0.025	1.50
MRPL24	mitochondrial ribosomal protein L24	< 0.001	1.64	< 0.001	1.51
MRPL55	mitochondrial ribosomal protein L55	0.004	1.50	0.003	1.54
MRPS21	mitochondrial ribosomal protein S21	< 0.001	1.56	< 0.001	1.55
MSH4	mutS homolog 4 (E. coli)	0.011	-1.78	0.008	-1.85
MSX2	msh homeobox 2	0.007	1.54	< 0.001	2.19
MTHFD2	methylenetetrahydrofolate dehydrogenase (NADP+ dependent) 2, methenyltetrahydrofolate cyclohydrolase	0.001	2.47	0.001	2.25
MUC1	mucin 1, cell surface associated	< 0.001	1.67	< 0.001	1.82
MVK	mevalonate kinase	< 0.001	2.11	0.005	1.53
MYBL1	v-myb myeloblastosis viral oncogene homolog (avian)-like 1	0.020	1.55	0.020	1.55
MYCL1	v-myc avian myelocytomatosis viral oncogene lung carcinoma derived homolog	< 0.001	3.79	0.024	1.61
MYEOV2	myeloma overexpressed 2	< 0.001	1.88	0.001	1.62
MYO5A	myosin VA (heavy chain 12, myoxin)	< 0.001	1.51	< 0.001	1.58
NACC1	nucleus accumbens associated 1, BEN and BTB (POZ) domain containing	0.001	1.68	0.003	1.51
NAIP	NLR family, apoptosis inhibitory protein	0.004	-2.12	0.005	-2.09

NANS	N-acetylneuraminic acid synthase	< 0.001	2.25	< 0.001	2.05
NCAPG	non-SMC condensin I complex, subunit G	< 0.001	2.97	< 0.001	3.73
NCAPH	non-SMC condensin I complex, subunit H	0.001	2.40	0.013	1.78
NCEH1	neutral cholesterol ester hydrolase 1	< 0.001	-2.03	0.001	-1.96
NCF2	neutrophil cytosolic factor 2	< 0.001	-2.26	0.003	-1.63
NDC80	NDC80 homolog, kinetochore complex component (S. cerevisiae)	0.001	3.33	0.002	2.90
NDRG1	N-myc downstream regulated 1	< 0.001	-2.32	< 0.001	-2.39
NDUFA11	NADH dehydrogenase (ubiquinone) 1 alpha subcomplex, 11, 14.7kDa	< 0.001	2.27	< 0.001	2.01
NDUFA13	NADH dehydrogenase (ubiquinone) 1 alpha subcomplex, 13	< 0.001	1.58	< 0.001	1.82
NDUFA3	NADH dehydrogenase (ubiquinone) 1 alpha subcomplex, 3, 9kDa	< 0.001	1.76	0.002	1.52
NDUFA4	NADH dehydrogenase (ubiquinone) 1 alpha subcomplex, 4, 9kDa	< 0.001	1.98	< 0.001	1.87
NDUFAF4	NADH dehydrogenase (ubiquinone) 1 alpha subcomplex, assembly factor 4	< 0.001	1.88	0.003	1.60
NDUFS6	NADH dehydrogenase (ubiquinone) Fe-S protein 6, 13kDa (NADH-coenzyme Q reductase)	< 0.001	1.51	< 0.001	1.56
NDUFS8	NADH dehydrogenase (ubiquinone) Fe-S protein 8, 23kDa (NADH-coenzyme Q reductase)	< 0.001	1.78	< 0.001	1.61
NEDD8	neural precursor cell expressed, developmentally down-regulated 8	0.013	1.51	0.007	1.59
NEK6	NIMA (never in mitosis gene a)-related kinase 6	< 0.001	-1.76	0.001	-1.54
NFAT5	nuclear factor of activated T-cells 5, tonicity-responsive	0.001	-1.59	< 0.001	-2.05
NFE2L3	nuclear factor (erythroid-derived 2)-like 3	< 0.001	1.62	< 0.001	1.51
NHEDC2	solute carrier family 9, subfamily B (cation proton antiporter 2), member 2 (SLC9B2)	< 0.001	3.30	< 0.001	2.92
NHP2	NHP2 ribonucleoprotein homolog (yeast)	< 0.001	2.06	0.003	1.68
NID1	nidogen 1	0.026	-1.61	0.013	1.74
NKD1	naked cuticle homolog 1 (Drosophila)	< 0.001	-1.89	< 0.001	-3.40
NME1-2	non-metastatic cells 1, protein (NM23A) expressed in	0.002	1.64	< 0.001	1.97
NOMO2	NODAL modulator 2	< 0.001	2.15	< 0.001	1.77
NOX4	NADPH oxidase 4	0.001	3.00	0.011	2.15
NPAS3	neuronal PAS domain protein 3	0.001	-1.61	< 0.001	-1.70
NPC1	Niemann-Pick disease, type C1	0.018	-1.51	0.001	-1.94
NPNT	nephronectin	0.044	-1.67	0.005	-2.27
NPR2	natriuretic peptide receptor B/guanylate cyclase B (atrionatriuretic peptide receptor B)	0.001	1.76	< 0.001	2.13
NR3C2	nuclear receptor subfamily 3, group C, member 2	< 0.001	-1.96	< 0.001	-2.00
NSDHL	NAD(P) dependent steroid dehydrogenase-like	< 0.001	3.73	< 0.001	2.13
NT5C	5', 3'-nucleotidase, cytosolic	< 0.001	2.93	< 0.001	2.09
NTS	neurotensin	< 0.001	32.44	< 0.001	21.57

NUB1	negative regulator of ubiquitin-like proteins 1	< 0.001	-1.64	< 0.001	-1.51
NUCB1	nucleobindin 1	< 0.001	2.30	0.004	1.74
NUCB2	nucleobindin 2	< 0.001	2.88	< 0.001	2.85
NUDT16	nudix (nucleoside diphosphate linked moiety X)-type motif 16	0.001	1.52	< 0.001	1.76
NUMA1	nuclear mitotic apparatus protein 1	0.001	-1.58	< 0.001	-1.74
NUS1	nuclear undecaprenyl pyrophosphate synthase 1 homolog (S. cerevisiae)	< 0.001	1.91	< 0.001	1.67
NUSAP1	nucleolar and spindle associated protein 1	< 0.001	2.63	0.009	1.65
NXPH4	neurexophilin 4	0.001	1.58	0.001	1.63
OAS2	2'-5'-oligoadenylate synthetase 2, 69/71kDa	0.012	-2.09	0.037	-1.76
OGT	O-linked N-acetylglucosamine (GlcNAc) transferase	< 0.001	-1.63	0.001	-1.51
OLA1	Obg-like ATPase 1	< 0.001	1.53	< 0.001	1.53
OR51E1	olfactory receptor, family 51, subfamily E, member 1	0.001	-2.50	0.007	-1.97
ORC1	origin recognition complex, subunit 1	0.001	2.91	0.005	2.29
OSTC	oligosaccharyltransferase complex subunit	< 0.001	2.35	< 0.001	2.14
OTUD1	OTU domain containing 1	0.001	-1.77	< 0.001	-2.04
OXCT1	3-oxoacid CoA transferase 1	< 0.001	1.64	< 0.001	1.76
P2RY6	pyrimidinergic receptor P2Y, G-protein coupled, 6	0.006	-1.69	0.014	-1.56
P4HA2	prolyl 4-hydroxylase, alpha polypeptide II	0.001	1.61	< 0.001	1.88
P4HB	prolyl 4-hydroxylase, beta polypeptide	< 0.001	2.34	< 0.001	1.92
PAF	KIAA0101 ortholog	< 0.001	3.46	0.003	1.96
PAK3	p21 protein (Cdc42/Rac)-activated kinase 3	< 0.001	1.96	< 0.001	2.04
PAQR5	progestin and adipoQ receptor family member V	0.012	-1.81	< 0.001	-3.39
PARP9	poly (ADP-ribose) polymerase family, member 9	0.001	-1.72	0.001	-1.74
PBK	PDZ binding kinase	0.001	3.02	0.004	2.47
PCK2	phosphoenolpyruvate carboxykinase 2 (mitochondrial)	0.001	2.05	0.007	1.62
PCMTD1	protein-L-isoaspartate (D-aspartate) O-methyltransferase domain containing 1	< 0.001	-2.02	< 0.001	-1.67
PCMTD2	protein-L-isoaspartate (D-aspartate) O-methyltransferase domain containing 2	< 0.001	-1.72	< 0.001	-1.73
PCNA	proliferating cell nuclear antigen	0.001	2.23	0.002	1.96
PCSK7	proprotein convertase subtilisin/kexin type 7	0.001	1.59	0.001	1.54
PDE12	phosphodiesterase 12	< 0.001	2.10	< 0.001	1.84
PDE6C	phosphodiesterase 6C, cGMP-specific, cone, alpha prime	< 0.001	-1.50	< 0.001	-1.72
PDE8B	phosphodiesterase 8B	< 0.001	1.84	< 0.001	1.77
PDIA4	protein disulfide isomerase family A, member 4	< 0.001	6.19	< 0.001	4.81
PDIA5	protein disulfide isomerase family A, member 5	< 0.001	2.70	< 0.001	2.60
PDIA6	protein disulfide isomerase family A, member 6	0.001	1.88	< 0.001	2.01
PDK4	pyruvate dehydrogenase kinase, isozyme 4	0.001	-3.93	0.019	-2.24

PDLIM4	PDZ and LIM domain 4	< 0.001	2.33	0.001	1.78
PDZD11	PDZ domain containing 11	0.001	1.75	0.001	1.75
PEBP1	phosphatidylethanolamine binding protein 1	0.002	1.53	0.001	1.57
PELI2	pellino E3 ubiquitin protein ligase family member 2	0.002	-1.65	0.006	-1.50
PEMT	phosphatidylethanolamine N-methyltransferase	< 0.001	2.86	< 0.001	3.36
PFKFB3	6-phosphofructo-2-kinase/fructose-2,6-biphosphatase 3	0.007	-1.75	0.001	-2.16
PFN1	profilin 1	0.004	1.73	0.011	1.56
PGM3	phosphoglucomutase 3	0.001	1.68	0.001	1.65
PGP	phosphoglycolate phosphatase	< 0.001	2.87	< 0.001	1.95
PGR	progesterone receptor-like	0.008	1.84	< 0.001	1.92
PHLDA2	pleckstrin homology-like domain, family A, member 2	0.028	1.62	0.005	2.02
PHPT1	phosphohistidine phosphatase 1	0.006	1.64	0.009	1.59
PHYHD1	phytanoyl-CoA dioxygenase domain containing 1	< 0.001	-1.52	< 0.001	-1.60
PIGB	phosphatidylinositol glycan anchor biosynthesis, class B	< 0.001	1.95	< 0.001	2.33
PIGK	phosphatidylinositol glycan anchor biosynthesis, class K	< 0.001	1.55	< 0.001	1.52
PIGW	phosphatidylinositol glycan anchor biosynthesis, class W	0.017	1.50	0.007	1.62
PIM1	pim-1 oncogene	0.001	-1.71	0.002	-1.54
PINX1	PIN2/TERF1 interacting, telomerase inhibitor 1	0.002	1.64	< 0.001	1.87
PKHD1	polycystic kidney and hepatic disease 1 (autosomal recessive)	0.001	-2.70	< 0.001	-3.00
PKIA	protein kinase (cAMP-dependent, catalytic) inhibitor alpha	0.038	1.65	0.004	2.26
PLA2G10	phospholipase A2, group X	0.001	2.52	0.002	2.08
PLA2G4D	phospholipase A2, group IVD (cytosolic)	< 0.001	6.61	< 0.001	7.64
PLA2G4F	phospholipase A2, group IVF	0.001	3.66	< 0.001	6.85
PLAC8	placenta-specific 8	0.006	-2.79	0.040	-1.98
PLAG1	pleiomorphic adenoma gene 1	0.002	-2.45	0.023	-1.76
PLEKHG7	pleckstrin homology domain containing, family G (with RhoGef domain) member 7	0.042	1.97	0.016	2.36
PLEKHM3	pleckstrin homology domain containing, family M, member 3	< 0.001	-1.53	< 0.001	-1.57
PLK4	polo-like kinase 4	0.004	2.10	0.026	1.66
PLXNA2	plexin A2	0.001	-1.60	0.002	-1.53
PNO1	partner of NOB1 homolog (S. cerevisiae)	0.001	1.63	< 0.001	1.71
PNPO	pyridoxamine 5'-phosphate oxidase	< 0.001	1.58	< 0.001	1.56
POLG2	polymerase (DNA directed), gamma 2, accessory subunit	< 0.001	-1.51	< 0.001	-1.53
POLQ	polymerase (DNA directed), theta	0.001	1.54	< 0.001	1.73
POLR3K	polymerase (RNA) III (DNA directed) polypeptide K, 12.3 kDa	< 0.001	1.59	< 0.001	1.56

POP5	processing of precursor 5, ribonuclease P/MRP subunit (S. cerevisiae)	< 0.001	1.91	0.003	1.58
PPA1	pyrophosphatase (inorganic) 1	0.001	2.86	< 0.001	3.15
PPAP2B	phosphatidic acid phosphatase type 2B	< 0.001	-2.00	< 0.001	-1.98
PPAPDC1B	phosphatidic acid phosphatase type 2 domain containing 1B	< 0.001	2.39	< 0.001	1.53
PPIA	peptidylprolyl isomerase A (cyclophilin A)	< 0.001	1.84	< 0.001	1.79
PPIB	peptidylprolyl isomerase B (cyclophilin B)	< 0.001	2.11	< 0.001	2.04
PPIC	peptidylprolyl isomerase C (cyclophilin C)	< 0.001	2.32	< 0.001	2.50
PRC1	protein regulator of cytokinesis 1	0.001	2.45	0.006	1.83
PRDX1	peroxiredoxin 1	< 0.001	1.79	< 0.001	1.80
PRDX4	peroxiredoxin 4	< 0.001	1.71	< 0.001	1.63
PRELID1	PRELI domain containing 1	< 0.001	1.72	< 0.001	1.78
PRKCB	protein kinase C, beta	0.001	3.53	0.004	2.73
PRKCDBP	protein kinase C, delta binding protein	< 0.001	4.54	0.001	2.17
PRKD1	protein kinase D1	< 0.001	-1.75	< 0.001	-2.07
PRND	prion protein 2 (dublet)	< 0.001	18.30	< 0.001	12.36
PROX1	prospero homeobox 1	0.009	-1.50	0.008	-1.51
PRR11	proline rich 11	0.004	2.36	0.019	1.88
PRRG1	proline rich Gla (G-carboxyglutamic acid) 1	0.010	-1.51	< 0.001	-2.89
PRUNE2	prune homolog 2 (Drosophila)	0.002	-1.59	0.003	-1.55
PSAT1	phosphoserine aminotransferase 1	< 0.001	3.42	< 0.001	4.03
PSMA1	proteasome (prosome, macropain) subunit, alpha type, 1	< 0.001	1.69	< 0.001	1.62
PSMB5	proteasome (prosome, macropain) subunit, beta type, 5	< 0.001	1.65	< 0.001	1.66
PSMC5	proteasome (prosome, macropain) 26S subunit, ATPase, 5	< 0.001	1.53	< 0.001	1.53
PSMD14	proteasome (prosome, macropain) 26S subunit, non-ATPase, 14	< 0.001	1.73	< 0.001	1.71
PSMG2	proteasome (prosome, macropain) assembly chaperone 2	< 0.001	1.79	< 0.001	1.80
PSPH	phosphoserine phosphatase	< 0.001	2.77	0.001	2.55
PTCH1	patched 1	< 0.001	-1.76	< 0.001	-2.14
PTK2B	PTK2B protein tyrosine kinase 2 beta	0.001	-1.63	0.001	-1.60
PTPN14	protein tyrosine phosphatase, non-receptor type 14	0.001	-1.56	< 0.001	-1.64
PTPRR	protein tyrosine phosphatase, receptor type, R	0.021	-1.59	< 0.001	-2.52
PYCR1	pyrroline-5-carboxylate reductase 1	< 0.001	3.41	< 0.001	3.41
RAB33A	RAB33A, member RAS oncogene family	0.001	2.16	0.014	-1.56
RABAC1	Rab acceptor 1 (prenylated)	< 0.001	2.14	< 0.001	1.72
RABGGTB	Rab geranylgeranyltransferase, beta subunit	< 0.001	1.51	< 0.001	1.55
RAN	RAN, member RAS oncogene family	0.001	1.57	0.001	1.69
RASAL1	RAS protein activator like 1 (GAP1 like)	< 0.001	-2.23	< 0.001	-3.05

RASGRP1	RAS guanyl releasing protein 1 (calcium and DAG-regulated)	0.009	-2.34	0.017	-2.09
RASSF9	Ras association (RalGDS/AF-6) domain family (N-terminal) member 9	< 0.001	2.08	0.001	1.71
RBM43	RNA binding motif protein 43	0.001	-2.00	< 0.001	-2.30
RCC2	regulator of chromosome condensation 2	< 0.001	1.67	< 0.001	1.62
RCN1	reticulocalbin 1, EF-hand calcium binding domain	< 0.001	1.93	< 0.001	2.42
RCN2	reticulocalbin 2, EF-hand calcium binding domain	< 0.001	3.26	< 0.001	2.31
RCN3	reticulocalbin 3, EF-hand calcium binding domain	< 0.001	1.88	< 0.001	1.77
RDH11	retinol dehydrogenase 11 (all-trans/9-cis/11-cis)	< 0.001	2.66	< 0.001	1.59
RELL1	RELT-like 1	< 0.001	1.90	< 0.001	1.56
REPS1	RALBP1 associated Eps domain containing 1	< 0.001	-1.91	< 0.001	-2.08
REXO2	REX2, RNA exonuclease 2 homolog (S. cerevisiae)	< 0.001	2.09	< 0.001	2.34
RFC4	replication factor C (activator 1) 4, 37kDa	0.009	1.85	0.016	1.72
RGS4	regulator of G-protein signaling 4	0.006	2.64	0.024	2.07
RHOBTB3	Rho-related BTB domain containing 3	< 0.001	-1.61	< 0.001	-1.92
RHOC	ras homolog gene family, member C	0.002	1.65	0.001	1.72
RIMKLB	ribosomal modification protein rimK-like family member B	< 0.001	-1.74	< 0.001	-1.57
RNASE4	ribonuclease, RNase A family, 4	0.004	-2.04	0.016	-1.71
RNASEL	ribonuclease L (2',5'-oligoisoadenylate synthetase-dependent)	0.003	-1.73	0.004	-1.70
RND1	Rho family GTPase 1	< 0.001	3.56	< 0.001	2.40
RNF125	ring finger protein 125	< 0.001	-1.54	< 0.001	-1.74
ROBLD3	late endosomal/lysosomal adaptor, MAPK and MTOR activator 2 (LAMTOR2)	< 0.001	1.72	0.001	1.54
RORC	RAR-related orphan receptor C	< 0.001	-2.04	< 0.001	-2.03
RP2	retinitis pigmentosa 2 (X-linked recessive)	< 0.001	1.70	< 0.001	1.88
RPL27	ribosomal protein L27	< 0.001	1.62	< 0.001	1.51
RPL36A	ribosomal protein L36a-like	0.003	1.69	0.006	1.59
RPL7	ribosomal protein L7	< 0.001	1.74	< 0.001	1.70
RPN1	ribophorin I	< 0.001	1.84	< 0.001	1.74
RPN2	ribophorin II	< 0.001	1.92	< 0.001	1.78
RPS10	ribosomal protein S10	< 0.001	1.86	< 0.001	1.94
RPS25	ribosomal protein S25	0.001	1.75	0.001	1.78
RPS4Y1	ribosomal protein S4, Y-linked 1	< 0.001	1.52	< 0.001	1.62
RPS6KA6	ribosomal protein S6 kinase, 90kDa, polypeptide 6	< 0.001	-1.59	< 0.001	-1.55
RRM2	ribonucleotide reductase M2	< 0.001	6.52	0.001	4.92
RUNDC3B	RUN domain containing 3B	< 0.001	-3.17	< 0.001	-2.98
SAE1	SUMO1 activating enzyme subunit 1	< 0.001	1.67	< 0.001	1.59
SAMD9	sterile alpha motif domain containing 9	0.007	-2.17	0.004	-2.36
SAR1A	SAR1 homolog A (S. cerevisiae)	< 0.001	1.66	< 0.001	1.67

SARS	seryl-tRNA synthetase	< 0.001	1.61	< 0.001	1.74
SAT1	spermidine/spermine N1-acetyltransferase 1	< 0.001	-1.87	< 0.001	-1.85
SC5DL	sterol-C5-desaturase (ERG3 delta-5-desaturase homolog, S. cerevisiae)-like	< 0.001	1.60	< 0.001	1.52
SCARB1	scavenger receptor class B, member 1	0.017	1.69	0.039	1.54
SCD	stearoyl-CoA desaturase (delta-9-desaturase)	< 0.001	2.19	< 0.001	1.73
SCP2	sterol carrier protein 2	0.003	-1.70	< 0.001	-2.06
SCRN1	secernin 1	< 0.001	-1.73	< 0.001	-1.85
SCRN3	secernin 3	0.004	-1.79	0.013	-1.60
SDF2L1	stromal cell-derived factor 2-like 1	< 0.001	9.29	< 0.001	7.31
SEC11C	SEC11 homolog C (S. cerevisiae)	< 0.001	3.21	0.003	1.64
SEC24D	SEC24 family, member D (S. cerevisiae)	< 0.001	2.23	< 0.001	1.96
SEC61B	Sec61 beta subunit	< 0.001	2.02	< 0.001	1.85
SEC61G	Sec61 gamma subunit	< 0.001	1.99	< 0.001	1.70
SELK	selenoprotein K	< 0.001	2.60	< 0.001	2.27
SELL	selectin L	0.001	-2.88	0.003	-2.42
SELM	selenoprotein M	0.005	1.86	0.002	2.12
SELS	VCP-interacting membrane protein (VIMP)	< 0.001	2.61	< 0.001	2.21
SERF1A	small EDRK-rich factor 1A (telomeric)	0.001	1.58	< 0.001	1.81
SERINC2	serine incorporator 2	0.001	1.71	0.002	1.59
SERINC3	serine incorporator 3	< 0.001	-1.60	< 0.001	-1.52
SERINC5	serine incorporator 5	0.002	-1.54	0.002	-1.57
SERPINA5	serpin peptidase inhibitor, clade A (alpha-1 antiproteinase, antitrypsin), member 5	0.004	2.67	0.006	2.46
SERPINE3	serpin peptidase inhibitor, clade E (nexin, plasminogen activator inhibitor type 1), member 3	< 0.001	6.54	0.013	2.43
SERPINH1	serpin peptidase inhibitor, clade H (heat shock protein 47), member 1, (collagen binding protein 1)	< 0.001	3.27	< 0.001	3.03
SFXN2	sideroflexin 2	< 0.001	1.82	0.002	1.55
SGOL1	shugoshin-like 1 (S. pombe)	< 0.001	2.48	0.001	1.98
SHCBP1	SHC SH2-domain binding protein 1	< 0.001	3.29	0.003	2.00
SHISA2	shisa homolog 2 (Xenopus laevis)	< 0.001	2.39	< 0.001	1.86
SHISA5	shisa homolog 5 (Xenopus laevis)	< 0.001	1.87	< 0.001	1.62
SHMT2	serine hydroxymethyltransferase 2 (mitochondrial)	< 0.001	1.90	< 0.001	1.63
SIGMAR1	sigma non-opioid intracellular receptor 1	< 0.001	2.04	0.001	1.75
SIL1	SIL1 homolog, endoplasmic reticulum chaperone (S. cerevisiae)	< 0.001	1.73	< 0.001	1.78
SLC15A1	solute carrier family 15 (oligopeptide transporter), member 1	0.045	-1.57	0.001	-2.55
SLC16A2	solute carrier family 16, member 2 (monocarboxylic acid transporter 8)	0.021	1.53	0.015	1.58
SLC16A9	solute carrier family 16, member 9 (monocarboxylic acid transporter 9)	0.001	-1.70	0.004	-1.54

SLC17A9	solute carrier family 17, member 9	< 0.001	5.02	< 0.001	3.88
SLC19A1	solute carrier family 19 (folate transporter), member 1	< 0.001	1.82	< 0.001	1.83
SLC1A1	solute carrier family 1 (neuronal/epithelial high affinity glutamate transporter, system Xag), member 1	0.005	-1.87	0.001	-2.42
SLC1A5	solute carrier family 1 (neutral amino acid transporter), member 5	0.001	2.48	0.013	1.68
SLC25A15	solute carrier family 25 (mitochondrial carrier; ornithine transporter) member 15	< 0.001	2.01	< 0.001	1.94
SLC25A28	solute carrier family 25, member 28	0.001	-1.63	0.001	-1.64
SLC25A35	solute carrier family 25, member 35	< 0.001	3.61	< 0.001	3.03
SLC25A5	solute carrier family 25 (mitochondrial carrier; adenine nucleotide translocator), member 5	< 0.001	2.26	< 0.001	2.06
SLC2A10	solute carrier family 2 (facilitated glucose transporter), member 10	< 0.001	9.54	< 0.001	6.69
SLC30A4	solute carrier family 30 (zinc transporter), member 4	0.004	2.34	0.039	1.68
SLC30A7	solute carrier family 30 (zinc transporter), member 7	< 0.001	1.67	< 0.001	1.67
SLC33A1	solute carrier family 33 (acetyl-CoA transporter), member 1	< 0.001	1.69	< 0.001	1.58
SLC35B1	solute carrier family 35, member B1	< 0.001	3.93	< 0.001	2.69
SLC35F1	solute carrier family 35, member F1	< 0.001	2.12	0.001	1.57
SLC41A2	solute carrier family 41, member 2	< 0.001	2.97	0.004	1.66
SLC6A15	solute carrier family 6 (neutral amino acid transporter), member 15	0.006	-2.61	0.015	-2.21
SLC6A9	solute carrier family 6 (neurotransmitter transporter, glycine), member 9	0.006	-1.81	< 0.001	-2.72
SLC7A11	solute carrier family 7 (anionic amino acid transporter light chain, xc- system), member 11	< 0.001	6.09	< 0.001	8.92
SLC7A5	solute carrier family 7 (amino acid transporter light chain, L system), member 5	< 0.001	3.37	< 0.001	2.42
SLITRK5	SLIT and NTRK-like family, member 5	0.004	-2.04	0.009	-1.88
SMAD3	SMAD family member 3	< 0.001	-1.66	< 0.001	-2.19
SMC2	structural maintenance of chromosomes 2	0.003	1.65	0.008	1.51
SMOX	spermine oxidase	0.034	-1.52	0.005	-1.91
SMTNL2	smoothelin-like 2	0.005	1.53	0.005	1.54
SMYD2	SET and MYND domain containing 2	0.001	1.70	< 0.001	1.95
SNAI2	snail homolog 2 (Drosophila)	< 0.001	2.07	< 0.001	2.56
SNRNP25	small nuclear ribonucleoprotein 25kDa (U11/U12)	< 0.001	2.29	< 0.001	2.01
SNRPD1	small nuclear ribonucleoprotein D1 polypeptide 16kDa	0.003	1.71	0.003	1.68
SNRPG	small nuclear ribonucleoprotein polypeptide G	< 0.001	1.85	< 0.001	1.72
SNTB1	syntrophin, beta 1 (dystrophin-associated protein A1, 59kDa, basic component 1)	0.010	-1.74	0.002	-2.16
SOCS2	suppressor of cytokine signaling 2	0.004	1.52	< 0.001	1.83
SPAG5	sperm associated antigen 5	< 0.001	2.98	< 0.001	2.19

SPATA1	spermatogenesis associated 1	0.002	-1.61	< 0.001	-1.86
SPC25	SPC25, NDC80 kinetochore complex component, homolog (S. cerevisiae)	< 0.001	2.35	0.008	1.70
SPCS1	signal peptidase complex subunit 1 homolog (S. cerevisiae)	< 0.001	1.59	< 0.001	1.86
SPCS3	signal peptidase complex subunit 3 homolog (S. cerevisiae)	< 0.001	1.89	< 0.001	1.91
SPRY1	sprouty homolog 1, antagonist of FGF signaling (Drosophila)	0.001	-2.27	< 0.001	-2.92
SQLE	squalene epoxidase	< 0.001	3.75	0.005	1.63
SRM	spermidine synthase	< 0.001	3.08	< 0.001	2.23
SRPR	signal recognition particle receptor (docking protein)	< 0.001	1.70	< 0.001	1.52
SSR3	signal sequence receptor, gamma (translocon-associated protein gamma)	< 0.001	1.67	< 0.001	1.79
SSR4	signal sequence receptor, delta	< 0.001	1.66	< 0.001	1.79
ST3GAL5	ST3 beta-galactoside alpha-2,3-sialyltransferase 5	0.007	1.53	< 0.001	2.86
ST3GAL6	ST3 beta-galactoside alpha-2,3-sialyltransferase 6	< 0.001	-2.18	< 0.001	-2.72
ST6GAL2	ST6 beta-galactosamide alpha-2,6-sialyltranferase 2	0.013	2.15	0.003	2.70
ST6GALNAC2	ST6 (alpha-N-acetyl-neuraminyl-2,3-beta-galactosyl-1,3)-N-acetylgalactosaminide alpha-2,6-sialyltransferase 2	< 0.001	1.89	< 0.001	2.69
ST7	suppression of tumorigenicity 7	< 0.001	1.54	< 0.001	1.51
STEAP1	six transmembrane epithelial antigen of the prostate 1	< 0.001	3.21	0.036	1.56
STMN1	stathmin 1	0.001	2.31	0.009	1.83
STRA13	stimulated by retinoic acid 13 homolog (mouse)	< 0.001	1.90	0.004	1.53
STRA6	stimulated by retinoic acid gene 6 homolog (mouse)	0.018	3.26	< 0.001	11.22
STT3A	STT3, subunit of the oligosaccharyltransferase complex, homolog A (S. cerevisiae)	< 0.001	1.59	< 0.001	1.56
STX17	syntaxin 17	0.001	-1.50	< 0.001	-1.66
STX19	syntaxin 19	< 0.001	-1.98	0.001	-1.65
SUCLG1	succinate-CoA ligase, alpha subunit	< 0.001	1.53	< 0.001	1.54
SUFU	suppressor of fused homolog (Drosophila)	< 0.001	-1.56	< 0.001	-1.52
SUMF2	sulfatase modifying factor 2	0.001	1.72	0.001	1.82
SUN2	Sad1 and UNC84 domain containing 2	0.001	-1.64	< 0.001	-1.94
SUOX	sulfite oxidase	< 0.001	1.91	< 0.001	1.59
SURF4	surfeit 4	< 0.001	1.94	< 0.001	1.72
SYT17	synaptotagmin XVII	0.009	1.81	0.012	1.76
SYVN1	synovial apoptosis inhibitor 1, synoviolin	< 0.001	2.34	< 0.001	1.57
TACC1	transforming, acidic coiled-coil containing protein 1	0.001	-1.97	< 0.001	-2.51
TACR3	tachykinin receptor 3	< 0.001	-3.27	< 0.001	-2.45
TAGAP	T-cell activation RhoGTPase activating protein	0.022	-1.80	0.019	-1.83

TARS	threonyl-tRNA synthetase	< 0.001	1.68	< 0.001	2.09
TARSL2	threonyl-tRNA synthetase-like 2	0.002	-1.83	0.014	-1.51
TBC1D8B	TBC1 domain family, member 8B (with GRAM domain)	< 0.001	1.82	0.001	1.59
TCF19	transcription factor 19	< 0.001	1.94	0.008	1.50
TCP11L2	t-complex 11 (mouse)-like 2	< 0.001	-1.95	< 0.001	-1.74
TET2	tet methylcytosine dioxygenase 2	< 0.001	-1.53	< 0.001	-1.51
TFF3	trefoil factor 3 (intestinal)	0.045	-1.60	< 0.001	-4.98
TFRC	transferrin receptor (p90, CD71)	< 0.001	1.58	< 0.001	1.59
THOC4	THO complex 4 (ALYREF)	< 0.001	1.67	< 0.001	1.65
THRA	thyroid hormone receptor, alpha	< 0.001	-1.58	< 0.001	-1.59
THTPA	thiamine triphosphatase	0.009	-1.53	0.004	-1.66
THUMPD2	THUMP domain containing 2	< 0.001	-1.56	< 0.001	-1.54
TIGAR	chromosome 5 open reading frame, human C12orf5	< 0.001	1.75	< 0.001	1.58
TIMD4	T-cell immunoglobulin and mucin domain containing 4	0.004	-1.85	0.010	-1.67
TIMM50	translocase of inner mitochondrial membrane 50 homolog (S. cerevisiae)	< 0.001	1.50	< 0.001	1.51
TIMP1	TIMP metallopeptidase inhibitor 1	< 0.001	2.34	0.007	1.61
TKT	transketolase	< 0.001	2.07	< 0.001	1.88
TLCD1	TLC domain containing 1	< 0.001	2.13	< 0.001	1.72
TLR1	toll-like receptor 1	< 0.001	-1.93	< 0.001	-1.93
TLR6	toll-like receptor 6	0.002	-1.98	0.003	-1.83
TLR8	toll-like receptor 8	0.004	-1.71	0.003	-1.75
TMED3	transmembrane emp24 protein transport domain containing 3	< 0.001	2.20	< 0.001	2.07
TMED9	transmembrane emp24 protein transport domain containing 9	< 0.001	2.39	< 0.001	1.83
TMEM111	ER membrane protein complex subunit 3	< 0.001	1.54	< 0.001	1.78
TMEM120A	transmembrane protein 120A	< 0.001	3.91	0.008	1.73
TMEM126A	transmembrane protein 126A	0.002	1.61	0.001	1.73
TMEM135	transmembrane protein 135	0.001	1.55	< 0.001	1.59
TMEM138	transmembrane protein 138	0.002	-2.06	0.006	-1.80
TMEM160	transmembrane protein 160	< 0.001	2.17	< 0.001	1.88
TMEM167A	transmembrane protein 167A	< 0.001	1.64	< 0.001	1.54
TMEM183A	transmembrane protein 183A	< 0.001	1.87	< 0.001	1.51
TMEM196	transmembrane protein 196	0.018	1.91	0.004	2.38
TMEM214	transmembrane protein 214	< 0.001	2.42	< 0.001	1.75
TMEM41B	transmembrane protein 41B	< 0.001	2.34	< 0.001	2.07
TMEM45A	transmembrane protein 45A	0.004	10.69	0.004	10.56
TMEM50B	transmembrane protein 50B	< 0.001	1.92	< 0.001	1.73
TMEM54	transmembrane protein 54	0.032	1.56	0.038	1.53

TMEM71	transmembrane protein 71	0.022	-1.66	0.001	-2.57
TMEM88B	transmembrane protein 88B	0.001	1.69	< 0.001	2.07
TMEM93	transmembrane protein 93	0.001	1.70	0.002	1.52
TMEM97	transmembrane protein 97	< 0.001	3.01	< 0.001	2.17
TMOD1	tropomodulin 1	0.005	3.76	0.040	2.35
TNFSF10	tumor necrosis factor (ligand) superfamily, member 10	0.001	-2.80	0.030	-1.75
TNFSF13B	tumor necrosis factor (ligand) superfamily, member 13b	0.022	-1.61	0.010	-1.75
TNIK	TRAF2 and NCK interacting kinase	0.002	-1.51	< 0.001	-1.95
TNS1	tensin 1	0.002	-1.81	0.001	-2.03
TP53I3	tumor protein p53 inducible protein 3	< 0.001	1.52	< 0.001	1.59
TPCN1	two pore segment channel 1	< 0.001	-1.78	< 0.001	-2.61
TPM3	tropomyosin 3	0.001	1.93	< 0.001	2.37
TPX2	TPX2, microtubule-associated, homolog (Xenopus laevis)	0.003	2.65	0.002	2.94
TRAIN-A	ribonuclease, RNase A family, 10 (non-active)	0.002	-2.16	< 0.001	-2.74
TRIB1	tribbles homolog 1 (Drosophila)	0.001	-2.41	0.010	-1.81
TRPM3	transient receptor potential cation channel, subfamily M, member 3	0.016	3.13	0.047	2.43
TSHZ2	teashirt zinc finger homeobox 2	< 0.001	-2.02	0.001	-1.77
TSSK3	testis-specific serine kinase 3	< 0.001	2.17	< 0.001	2.08
TSTA3	tissue specific transplantation antigen P35B	< 0.001	2.22	0.002	1.64
TTC33	tetratricopeptide repeat domain 33	< 0.001	-1.76	< 0.001	-1.68
TUBA1B	tubulin, alpha 1b	< 0.001	2.23	< 0.001	2.35
TUBA3E	tubulin, alpha 3e	< 0.001	1.52	< 0.001	1.76
TUBA4A	tubulin, alpha 4a	< 0.001	3.54	< 0.001	3.37
TUBA8	tubulin, alpha 8	< 0.001	2.03	< 0.001	2.27
TUBB2A	tubulin, beta 2B class IIb	0.012	1.68	< 0.001	2.50
TUBB2B	tubulin, beta 2B class IIb	< 0.001	2.81	< 0.001	2.31
TUBB3	tubulin, beta 3 class III	< 0.001	1.75	< 0.001	1.63
TUBG1	tubulin, gamma 1	< 0.001	1.83	< 0.001	1.60
TXK	TXK tyrosine kinase	< 0.001	-1.86	< 0.001	-2.00
TXNDC17	thioredoxin domain containing 17	0.001	2.12	0.017	1.55
TXNDC5	thioredoxin domain containing 5 (endoplasmic reticulum)	< 0.001	2.57	< 0.001	1.79
UBE2C	ubiquitin-conjugating enzyme E2C	0.001	2.42	0.028	1.60
UBIAD1	UbiA prenyltransferase domain containing 1	0.002	-1.55	0.002	-1.57
UCHL3	ubiquitin carboxyl-terminal esterase L3 (ubiquitin thiolesterase)	0.005	1.90	0.003	2.01
UFC1	ubiquitin-fold modifier conjugating enzyme 1	< 0.001	1.78	< 0.001	1.83
UFM1	ubiquitin-fold modifier 1	< 0.001	1.65	< 0.001	1.72
UGDH	UDP-glucose 6-dehydrogenase	< 0.001	1.94	0.001	1.83

UGGT1	UDP-glucose glycoprotein glucosyltransferase 1	< 0.001	2.01	< 0.001	1.82
UHRF1	ubiquitin-like with PHD and ring finger domains 1	< 0.001	3.11	0.002	2.26
ULK2	unc-51-like kinase 2 (C. elegans)	< 0.001	-1.56	< 0.001	-1.53
UPF0639	pleckstrin homology domain containing, family D (with coiled-coil domains) member 1	0.001	1.53	< 0.001	1.66
UPK1B	uroplakin 1B	0.034	2.15	0.009	2.79
UPK3B	uroplakin 3B	0.026	1.86	0.001	3.13
UPRT	uracil phosphoribosyltransferase (FUR1) homolog (S. cerevisiae)	< 0.001	1.70	< 0.001	1.68
UQCR11	ubiquinol-cytochrome c reductase, complex III subunit XI	< 0.001	1.91	< 0.001	1.89
UQCRQ	ubiquinol-cytochrome c reductase, complex III subunit VII, 9.5kDa	0.001	1.66	< 0.001	1.80
USP28	ubiquitin specific peptidase 28	0.004	-1.60	0.005	-1.55
UXT	ubiquitously-expressed transcript	< 0.001	1.95	< 0.001	1.90
VAMP1	vesicle-associated membrane protein 1 (synaptobrevin 1)	< 0.001	-1.92	< 0.001	-2.19
VCAM1	vascular cell adhesion molecule 1	< 0.001	-7.11	0.015	-2.28
VDR	vitamin D (1,25- dihydroxyvitamin D3) receptor	0.002	-1.62	0.005	-1.54
VIPR2	vasoactive intestinal peptide receptor 2	0.014	-1.83	< 0.001	-3.99
VSIG4	V-set and immunoglobulin domain containing 4	0.004	-2.04	0.003	-2.10
WBSCR22	Williams Beuren syndrome chromosome region 22	< 0.001	1.61	< 0.001	1.62
WDHD1	WD repeat and HMG-box DNA binding protein 1	< 0.001	2.43	< 0.001	2.70
WDR12	WD repeat domain 12	0.001	1.57	< 0.001	1.72
WDYHV1	WDYHV motif containing 1	0.001	1.63	0.002	1.52
WIPF3	WAS/WASL interacting protein family, member 3	< 0.001	-1.72	< 0.001	-3.39
XYLB	xylulokinase homolog (H. influenzae)	0.005	1.54	0.001	1.83
YARS	tyrosyl-tRNA synthetase	< 0.001	1.60	< 0.001	1.72
YBX1	Y box binding protein 1	< 0.001	1.58	< 0.001	1.52
YKT6	YKT6 v-SNARE homolog (S. cerevisiae)	< 0.001	2.02	< 0.001	1.78
YPEL3	yippee-like 3 (Drosophila)	< 0.001	-1.87	< 0.001	-2.20
ZBTB16	zinc finger and BTB domain containing 16	0.001	-4.62	< 0.001	-5.91
ZBTB20	zinc finger and BTB domain containing 20	< 0.001	-1.86	< 0.001	-1.65
ZCCHC12	zinc finger, CCHC domain containing 12	0.010	1.52	0.001	1.83
ZFP36L2	zinc finger protein 36, C3H type-like 2	< 0.001	-1.85	< 0.001	-2.66
ZKSCAN1	zinc finger with KRAB and SCAN domains 1	< 0.001	-1.57	< 0.001	-1.56
ZMAT1	zinc finger, matrin-type 1	< 0.001	-2.19	0.002	-1.69
ZNF180	zinc finger protein 180	< 0.001	-1.62	< 0.001	-1.57
ZNF226	zinc finger protein 226	< 0.001	-1.51	< 0.001	-1.58
ZNF547	zinc finger protein 547	< 0.001	-1.78	0.003	-1.53
ZNF821	zinc finger protein 821	0.001	-1.64	< 0.001	-2.33
ZPBP	zona pellucida binding protein	0.014	1.64	0.001	2.13
