# Supplementary material for: A transcriptomal analysis of bovine oviductal epithelial cells collected during the follicular phase versus the luteal phase of the estrous cycle
Source: Reprod Biol Endocrinol. 2015 Aug 5;13:84. doi: 10.1186/s12958-015-0077-1 (PMC4524109; doi:10.1186/s12958-015-0077-1)
Supplement: Additional file 5: — Supplementary Table 3. Enriched gene ontology classifications from up-regulated DEGS within epithelial cells of the ampulla in follicular versus luteal phase groups (P < 0.05). [file 12958_2015_77_MOESM5_ESM.pdf]

Supplementary Table 3. Enriched gene ontology classifications from up-regulated DEGS within epithelial cells of the ampulla in follicular versus luteal phase groups (P < 0.05).

Biological Processes			
**GO ID**	**Description**	**% of significant genes falling into category [a]**	**Enrichment P-value**
7049	cell cycle	18.78	< 0.001
6695	cholesterol biosynthetic process	70.59	< 0.001
51301	cell division	20.83	< 0.001
7067	mitosis	20.83	< 0.001
6457	protein folding	16.34	< 0.001
18208	peptidyl-proline modification	54.55	< 0.001
413	protein peptidyl-prolyl isomerization	54.55	< 0.001
30970	retrograde protein transport, ER to cytosol	100.00	< 0.001
22900	electron transport chain	15.91	< 0.001
8652	cellular amino acid biosynthetic process	35.00	< 0.001
6260	DNA replication	15.56	< 0.001
6810	transport	9.09	< 0.001
30433	ER-associated protein catabolic process	50.00	< 0.001
8299	isoprenoid biosynthetic process	35.29	< 0.001
16192	vesicle-mediated transport	12.21	< 0.001
6270	DNA-dependent DNA replication initiation	41.67	< 0.001
34501	protein localization to kinetochore	100.00	< 0.001
30968	endoplasmic reticulum unfolded protein response	38.46	< 0.001
6986	response to unfolded protein	38.46	< 0.001
7059	chromosome segregation	27.27	< 0.001
15031	protein transport	9.13	< 0.001
71569	protein ufmylation	75.00	< 0.001
9306	protein secretion	75.00	< 0.001
6662	glycerol ether metabolic process	31.25	< 0.001
6633	fatty acid biosynthetic process	17.02	< 0.001
6488	dolichol-linked oligosaccharide biosynthetic process	36.36	< 0.001
51258	protein polymerization	17.50	0.001
7399	nervous system development	15.38	0.001
18279	protein N-linked glycosylation via asparagine	50.00	0.001
16254	preassembly of GPI anchor in ER membrane	50.00	0.001
6099	tricarboxylic acid cycle	21.74	0.001
31647	regulation of protein stability	42.86	0.002
6915	apoptosis	8.86	0.002
45454	cell redox homeostasis	12.50	0.002
48205	COPI coating of Golgi vesicle	37.50	0.002
6891	intra-Golgi vesicle-mediated transport	33.33	0.004

6465	signal peptide processing	33.33	0.004
32981	mitochondrial respiratory chain complex I assembly	33.33	0.004
45047	protein targeting to ER	66.67	0.004
6563	L-serine metabolic process	66.67	0.004
6275	regulation of DNA replication	66.67	0.004
31116	positive regulation of microtubule polymerization	66.67	0.004
6564	L-serine biosynthetic process	66.67	0.004
21680	cerebellar Purkinje cell layer development	66.67	0.004
6065	UDP-glucuronate biosynthetic process	66.67	0.004
46653	tetrahydrofolate metabolic process	66.67	0.004
7492	endoderm development	66.67	0.004
40008	regulation of growth	21.05	0.005
6641	triglyceride metabolic process	30.00	0.005
44262	cellular carbohydrate metabolic process	27.27	0.007
6541	glutamine metabolic process	27.27	0.007
86	G2/M transition of mitotic cell cycle	27.27	0.007
6979	response to oxidative stress	12.77	0.007
6621	protein retention in ER lumen	50.00	0.008
6102	isocitrate metabolic process	50.00	0.008
6544	glycine metabolic process	50.00	0.008
10890	positive regulation of sequestering of triglyceride	50.00	0.008
45445	myoblast differentiation	50.00	0.008
6888	ER to Golgi vesicle-mediated transport	18.18	0.008
51603	proteolysis involved in cellular protein catabolic process	17.39	0.009
15986	ATP synthesis coupled proton transport	17.39	0.009
6890	retrograde vesicle-mediated transport, Golgi to ER	23.08	0.011
1942	hair follicle development	23.08	0.011
51437	positive regulation of ubiquitin-protein ligase activity involved in mitotic cell cycle	13.16	0.012
45787	positive regulation of cell cycle	40.00	0.013
48147	negative regulation of fibroblast proliferation	40.00	0.013
42761	very long-chain fatty acid biosynthetic process	40.00	0.013
45116	protein neddylation	40.00	0.013
48286	lung alveolus development	21.43	0.013
31145	anaphase-promoting complex-dependent proteasomal ubiquitin-dependent protein catabolic process	12.82	0.014
32436	positive regulation of proteasomal ubiquitin-dependent protein catabolic process	20.00	0.016
30855	epithelial cell differentiation	20.00	0.016
34644	cellular response to UV	33.33	0.018
8634	negative regulation of survival gene product expression	33.33	0.018
51297	centrosome organization	33.33	0.018
45109	intermediate filament organization	33.33	0.018

6829	zinc ion transport	33.33	0.018
6400	tRNA modification	33.33	0.018
7080	mitotic metaphase plate congression	33.33	0.018
8643	carbohydrate transport	18.75	0.019
30307	positive regulation of cell growth	18.75	0.019
6913	nucleocytoplasmic transport	18.75	0.019
8380	RNA splicing	7.81	0.020
6486	protein glycosylation	10.17	0.021
43123	positive regulation of I-kappaB kinase/NF-kappaB cascade	10.00	0.022
6468	protein phosphorylation	10.00	0.022
6886	intracellular protein transport	7.02	0.024
42110	T cell activation	28.57	0.025
60325	face morphogenesis	28.57	0.025
1958	endochondral ossification	28.57	0.025
6103	2-oxoglutarate metabolic process	28.57	0.025
42744	hydrogen peroxide catabolic process	28.57	0.025
187	activation of MAPK activity	16.67	0.027
6414	translational elongation	10.64	0.028
6916	anti-apoptosis	9.38	0.030
30308	negative regulation of cell growth	12.12	0.032
9566	fertilization	25.00	0.033
34394	protein localization at cell surface	25.00	0.033
51085	chaperone mediated protein folding requiring cofactor	25.00	0.033
6626	protein targeting to mitochondrion	25.00	0.033
16044	cellular membrane organization	10.20	0.033
6974	response to DNA damage stimulus	11.76	0.035
51436	negative regulation of ubiquitin-protein ligase activity involved in mitotic cell cycle	11.76	0.035
1666	response to hypoxia	11.43	0.038
42632	cholesterol homeostasis	14.29	0.040
7411	axon guidance	14.29	0.040
9968	negative regulation of signal transduction	14.29	0.040
45599	negative regulation of fat cell differentiation	22.22	0.041
1836	release of cytochrome c from mitochondria	22.22	0.041
6783	heme biosynthetic process	22.22	0.041
34599	cellular response to oxidative stress	22.22	0.041
70328	triglyceride homeostasis	22.22	0.041
45840	positive regulation of mitosis	22.22	0.041
8286	insulin receptor signaling pathway	22.22	0.041
7018	microtubule-based movement	7.95	0.043
30162	regulation of proteolysis	13.64	0.045
1974	blood vessel remodeling	20.00	0.050

Cellular Components			
5783	endoplasmic reticulum	24.81	< 0.001
5789	endoplasmic reticulum membrane	23.36	< 0.001
5737	cytoplasm	8.15	< 0.001
5739	mitochondrion	10.57	< 0.001
5788	endoplasmic reticulum lumen	42.22	< 0.001
16020	membrane	6.51	< 0.001
5829	cytosol	10.70	< 0.001
5634	nucleus	6.42	< 0.001
30176	integral to endoplasmic reticulum membrane	42.86	< 0.001
5743	mitochondrial inner membrane	13.84	< 0.001
42470	melanosome	27.27	< 0.001
5793	endoplasmic reticulum-Golgi intermediate compartment	52.94	< 0.001
5654	nucleoplasm	12.82	< 0.001
5856	cytoskeleton	9.85	< 0.001
5794	Golgi apparatus	9.34	< 0.001
139	Golgi membrane	13.71	< 0.001
5876	spindle microtubule	42.86	< 0.001
5694	chromosome	11.30	< 0.001
5792	microsome	16.90	< 0.001
32133	chromosome passenger complex	100.00	< 0.001
70469	respiratory chain	15.63	< 0.001
777	condensed chromosome kinetochore	24.00	< 0.001
8250	oligosaccharyltransferase complex	60.00	< 0.001
5730	nucleolus	8.61	0.001
5640	nuclear outer membrane	50.00	0.001
33116	endoplasmic reticulum-Golgi intermediate compartment membrane	50.00	0.001
15630	microtubule cytoskeleton	22.73	0.001
775	chromosome, centromeric region	22.73	0.001
5689	U12-type spliceosomal complex	28.57	0.001
48471	perinuclear region of cytoplasm	9.63	0.001
45263	proton-transporting ATP synthase complex, coupling factor F(o)	42.86	0.002
42555	MCM complex	42.86	0.002
5813	centrosome	12.70	0.002
33185	dolichol-phosphate-mannose synthase complex	66.67	0.004
31262	Ndc80 complex	66.67	0.004
5746	mitochondrial respiratory chain	30.00	0.005
5874	microtubule	8.70	0.005
5625	soluble fraction	13.64	0.005
31410	cytoplasmic vesicle	8.94	0.006
30126	COPI vesicle coat	27.27	0.007

5787	signal peptidase complex	50.00	0.008
940	condensed chromosome outer kinetochore	50.00	0.008
31616	spindle pole centrosome	50.00	0.008
31965	nuclear membrane	14.29	0.009
5747	mitochondrial respiratory chain complex I	14.29	0.009
785	chromatin	16.67	0.011
30496	midbody	13.51	0.011
5815	microtubule organizing center	12.82	0.014
34362	low-density lipoprotein particle	33.33	0.018
16442	RNA-induced silencing complex	33.33	0.018
502	proteasome complex	14.29	0.018
228	nuclear chromosome	28.57	0.025
71564	npBAF complex	28.57	0.025
5763	mitochondrial small ribosomal subunit	28.57	0.025
43234	protein complex	10.64	0.028
5740	mitochondrial envelope	25.00	0.033
299	integral to membrane of membrane fraction	25.00	0.033
5624	membrane fraction	8.33	0.035
5637	nuclear inner membrane	22.22	0.041
16021	integral to membrane	4.15	0.049
776	kinetochore	20.00	0.050
276	mitochondrial proton-transporting ATP synthase complex, coupling factor F(o)	20.00	0.050
34361	very-low-density lipoprotein particle	20.00	0.050
5811	lipid particle	20.00	0.050
5665	DNA-directed RNA polymerase II, core complex	20.00	0.050
**Molecular Functions**			
3756	protein disulfide isomerase activity	83.33	< 0.001
5528	FK506 binding	54.55	< 0.001
3755	peptidyl-prolyl cis-trans isomerase activity	20.45	< 0.001
16491	oxidoreductase activity	7.31	< 0.001
4579	dolichyl-diphosphooligosaccharide-protein glycotransferase activity	50.00	< 0.001
16740	transferase activity	8.06	< 0.001
16538	cyclin-dependent protein kinase regulator activity	75.00	< 0.001
30544	Hsp70 protein binding	60.00	< 0.001
15035	protein disulfide oxidoreductase activity	20.69	0.001
49	tRNA binding	33.33	0.001
42277	peptide binding	50.00	0.001
16787	hydrolase activity	6.08	0.001
166	nucleotide binding	5.06	0.001
5524	ATP binding	5.18	0.001

48037	cofactor binding	25.00	0.002
16874	ligase activity	8.59	0.003
9055	electron carrier activity	8.54	0.003
51082	unfolded protein binding	10.31	0.003
51787	misfolded protein binding	66.67	0.004
4768	stearoyl-CoA 9-desaturase activity	66.67	0.004
46923	ER retention sequence binding	66.67	0.004
4450	isocitrate dehydrogenase (NADP+) activity	66.67	0.004
16671	oxidoreductase activity, acting on a sulfur group of donors, disulfide as acceptor	66.67	0.004
4582	dolichyl-phosphate beta-D-mannosyltransferase activity	66.67	0.004
43022	ribosome binding	23.08	0.011
4656	procollagen-proline 4-dioxygenase activity	40.00	0.013
16765	transferase activity, transferring alkyl or aryl (other than methyl) groups	40.00	0.013
70628	proteasome binding	40.00	0.013
42301	phosphate binding	40.00	0.013
4862	cAMP-dependent protein kinase inhibitor activity	40.00	0.013
15078	hydrogen ion transmembrane transporter activity	15.38	0.014
16616	oxidoreductase activity, acting on the CH-OH group of donors, NAD or NADP as acceptor	14.81	0.016
50699	WW domain binding	33.33	0.018
4620	phospholipase activity	33.33	0.018
8121	ubiquinol-cytochrome-c reductase activity	28.57	0.025
5159	insulin-like growth factor receptor binding	28.57	0.025
287	magnesium ion binding	7.63	0.030
31593	polyubiquitin binding	25.00	0.033
50681	androgen receptor binding	25.00	0.033
16717	oxidoreductase activity, acting on paired donors, with oxidation of a pair of donors resulting in the reduction of molecular oxygen to two molecules of water	25.00	0.033
50750	low-density lipoprotein particle receptor binding	25.00	0.033
4860	protein kinase inhibitor activity	25.00	0.033
4298	threonine-type endopeptidase activity	15.00	0.035
42393	histone binding	14.29	0.040
51287	NAD binding	11.11	0.042
4129	cytochrome-c oxidase activity	10.81	0.046

[a] Indicates the percent of genes in that GO category that were differentially expressed within our dataset.
