# Supplementary material for: A transcriptomal analysis of bovine oviductal epithelial cells collected during the follicular phase versus the luteal phase of the estrous cycle
Source: Reprod Biol Endocrinol. 2015 Aug 5;13:84. doi: 10.1186/s12958-015-0077-1 (PMC4524109; doi:10.1186/s12958-015-0077-1)
Supplement: Additional file 6: — Supplementary Table 4. Enriched gene ontology classifications from down-regulated DEGS within epithelial cells of the ampulla in follicular versus luteal phase groups (P < 0.05). [file 12958_2015_77_MOESM6_ESM.pdf]

Supplementary Table 4. Enriched gene ontology classifications from down-regulated DEGS within epithelial cells of the ampulla in follicular versus luteal phase groups (P < 0.05).

Biological Processes			
**GO ID**	**Description**	**% of significant genes falling into category [a]**	**Enrichment P-value**
45087	innate immune response	12.28	< 0.001
35094	response to nicotine	66.67	0.001
51450	myoblast proliferation	66.67	0.001
43407	negative regulation of MAP kinase activity	25.00	0.001
30509	BMP signaling pathway	21.43	0.001
7165	signal transduction	3.73	0.001
48511	rhythmic process	50.00	0.001
35025	positive regulation of Rho protein signal transduction	50.00	0.001
16338	calcium-independent cell-cell adhesion	50.00	0.001
61036	positive regulation of cartilage development	50.00	0.001
43066	negative regulation of apoptosis	7.32	0.002
42493	response to drug	11.76	0.002
43123	positive regulation of I-kappaB kinase/NF-kappaB cascade	8.33	0.002
188	inactivation of MAPK activity	40.00	0.002
70374	positive regulation of ERK1 and ERK2 cascade	16.67	0.002
187	activation of MAPK activity	16.67	0.002
8104	protein localization	15.00	0.003
1759	organ induction	33.33	0.003
48535	lymph node development	33.33	0.003
14065	phosphatidylinositol 3-kinase cascade	33.33	0.003
8585	female gonad development	33.33	0.003
32967	positive regulation of collagen biosynthetic process	33.33	0.003
60395	SMAD protein signal transduction	33.33	0.003
6954	inflammatory response	7.04	0.004
8285	negative regulation of cell proliferation	6.85	0.005
33077	T cell differentiation in thymus	25.00	0.006
2244	hemopoietic progenitor cell differentiation	25.00	0.006
32496	response to lipopolysaccharide	11.54	0.007
51781	positive regulation of cell division	11.54	0.007
30335	positive regulation of cell migration	11.54	0.007
1525	angiogenesis	7.84	0.007
1836	release of cytochrome c from mitochondria	22.22	0.008
50853	B cell receptor signaling pathway	22.22	0.008
48008	platelet-derived growth factor receptor signaling pathway	22.22	0.008
7595	lactation	22.22	0.008
48873	homeostasis of number of cells within a tissue	22.22	0.008

48662	negative regulation of smooth muscle cell proliferation	20.00	0.009
8633	activation of pro-apoptotic gene products	20.00	0.009
1889	liver development	20.00	0.009
32757	positive regulation of interleukin-8 production	18.18	0.011
17148	negative regulation of translation	18.18	0.011
48661	positive regulation of smooth muscle cell proliferation	18.18	0.011
6974	response to DNA damage stimulus	8.82	0.015
30218	erythrocyte differentiation	15.38	0.016
7565	female pregnancy	15.38	0.016
1570	vasculogenesis	15.38	0.016
6916	anti-apoptosis	6.25	0.016
6470	protein dephosphorylation	8.11	0.018
7389	pattern specification process	14.29	0.018
45786	negative regulation of cell cycle	14.29	0.018
30168	platelet activation	14.29	0.018
33138	positive regulation of peptidyl-serine phosphorylation	14.29	0.018
6958	complement activation, classical pathway	14.29	0.018
32436	positive regulation of proteasomal ubiquitin-dependent protein catabolic process	13.33	0.021
43433	negative regulation of sequence-specific DNA binding transcription factor activity	13.33	0.021
42475	odontogenesis of dentine-containing tooth	13.33	0.021
45669	positive regulation of osteoblast differentiation	13.33	0.021
1568	blood vessel development	12.50	0.024
9887	organ morphogenesis	11.76	0.027
7605	sensory perception of sound	11.76	0.027
6917	induction of apoptosis	6.98	0.027
7519	skeletal muscle tissue development	11.11	0.030
7204	elevation of cytosolic calcium ion concentration	11.11	0.030
16525	negative regulation of angiogenesis	10.53	0.033
51607	defense response to virus	10.53	0.033
1934	positive regulation of protein phosphorylation	10.00	0.036
50731	positive regulation of peptidyl-tyrosine phosphorylation	9.52	0.040
1558	regulation of cell growth	9.09	0.043
1503	ossification	9.09	0.043
6366	transcription from RNA polymerase II promoter	9.09	0.043
71260	cellular response to mechanical stimulus	9.09	0.043
7179	transforming growth factor beta receptor signaling pathway	9.09	0.043
1822	kidney development	9.09	0.043
90090	negative regulation of canonical Wnt receptor signaling pathway	9.09	0.043
6754	ATP biosynthetic process	5.77	0.044
45663	positive regulation of myoblast differentiation	33.33	0.045

48513	organ development	33.33	0.045
40037	negative regulation of fibroblast growth factor receptor signaling pathway	33.33	0.045
42994	cytoplasmic sequestering of transcription factor	33.33	0.045
46632	alpha-beta T cell differentiation	33.33	0.045
45742	positive regulation of epidermal growth factor receptor signaling pathway	33.33	0.045
45576	mast cell activation	33.33	0.045
70371	ERK1 and ERK2 cascade	33.33	0.045
32926	negative regulation of activin receptor signaling pathway	33.33	0.045
43171	peptide catabolic process	33.33	0.045
42953	lipoprotein transport	33.33	0.045
45356	positive regulation of interferon-alpha biosynthetic process	33.33	0.045
6788	heme oxidation	33.33	0.045
19217	regulation of fatty acid metabolic process	33.33	0.045
30193	regulation of blood coagulation	33.33	0.045
32091	negative regulation of protein binding	33.33	0.045
60754	positive regulation of mast cell chemotaxis	33.33	0.045
35066	positive regulation of histone acetylation	33.33	0.045
6911	phagocytosis, engulfment	33.33	0.045
1906	cell killing	33.33	0.045
50892	intestinal absorption	33.33	0.045
60058	positive regulation of apoptosis involved in mammary gland involution	33.33	0.045
21904	dorsal/ventral neural tube patterning	33.33	0.045
32331	negative regulation of chondrocyte differentiation	33.33	0.045
45603	positive regulation of endothelial cell differentiation	33.33	0.045
48593	camera-type eye morphogenesis	33.33	0.045
60272	embryonic skeletal joint morphogenesis	33.33	0.045
60687	regulation of branching involved in prostate gland morphogenesis	33.33	0.045
70244	negative regulation of thymocyte apoptosis	33.33	0.045
72205	metanephric collecting duct development	33.33	0.045
9948	anterior/posterior axis specification	33.33	0.045
45103	intermediate filament-based process	33.33	0.045
15908	fatty acid transport	33.33	0.045
15820	leucine transport	33.33	0.045
48013	ephrin receptor signaling pathway	33.33	0.045
50770	regulation of axonogenesis	33.33	0.045
19216	regulation of lipid metabolic process	33.33	0.045
46890	regulation of lipid biosynthetic process	33.33	0.045
10518	positive regulation of phospholipase activity	33.33	0.045
51045	negative regulation of membrane protein ectodomain proteolysis	33.33	0.045

7262	STAT protein import into nucleus	33.33	0.045
45892	negative regulation of transcription, DNA-dependent	4.49	0.046
8150	biological_process	8.70	0.047
**Cellular Components**			
5886	plasma membrane	4.01	< 0.001
5737	cytoplasm	2.81	< 0.001
5576	extracellular region	3.09	< 0.001
5829	cytosol	3.06	0.004
5923	tight junction	9.30	0.004
10494	stress granule	25.00	0.006
9898	internal side of plasma membrane	20.00	0.009
5634	nucleus	2.04	0.013
30054	cell junction	3.83	0.015
5901	caveola	14.29	0.018
16023	cytoplasmic membrane-bounded vesicle	7.69	0.021
5792	microsome	5.63	0.023
34707	chloride channel complex	10.00	0.036
5624	membrane fraction	4.76	0.039
5730	nucleolus	3.35	0.040
31264	death-inducing signaling complex	33.33	0.045
31362	anchored to external side of plasma membrane	33.33	0.045
31252	cell leading edge	33.33	0.045
**Molecular Functions**			
4522	pancreatic ribonuclease activity	25.00	< 0.001
4888	transmembrane signaling receptor activity	11.63	< 0.001
4719	protein-L-isoaspartate (D-aspartate) O-methyltransferase activity	66.67	0.001
17171	serine hydrolase activity	66.67	0.001
43325	phosphatidylinositol-3,4-bisphosphate binding	66.67	0.001
3707	steroid hormone receptor activity	9.80	0.001
16504	peptidase activator activity	50.00	0.001
5178	integrin binding	17.65	0.002
4091	carboxylesterase activity	15.00	0.003
4519	endonuclease activity	9.52	0.004
42802	identical protein binding	5.34	0.004
3727	single-stranded RNA binding	28.57	0.005
5096	GTPase activator activity	6.41	0.007
4857	enzyme inhibitor activity	22.22	0.008
15293	symporter activity	7.55	0.008
8134	transcription factor binding	7.55	0.008
16805	dipeptidase activity	20.00	0.009
42056	chemoattractant activity	20.00	0.009

8430	selenium binding	18.18	0.011
8083	growth factor activity	4.69	0.013
5529	sugar binding	5.00	0.018
17124	SH3 domain binding	8.11	0.018
5484	SNAP receptor activity	12.50	0.024
19899	enzyme binding	7.32	0.024
31625	ubiquitin protein ligase binding	7.32	0.024
19901	protein kinase binding	5.48	0.025
8144	drug binding	11.76	0.027
8022	protein C-terminus binding	6.98	0.027
3690	double-stranded DNA binding	10.53	0.033
5520	insulin-like growth factor binding	10.00	0.036
16772	transferase activity, transferring phosphorus-containing groups	2.97	0.042
5102	receptor binding	4.60	0.043
43565	sequence-specific DNA binding	2.64	0.044
55106	ubiquitin-protein ligase regulator activity	33.33	0.045
4887	thyroid hormone receptor activity	33.33	0.045
30296	protein tyrosine kinase activator activity	33.33	0.045
51434	BH3 domain binding	33.33	0.045
4999	vasoactive intestinal polypeptide receptor activity	33.33	0.045
5283	sodium:amino acid symporter activity	33.33	0.045
8195	phosphatidate phosphatase activity	33.33	0.045
51378	serotonin binding	33.33	0.045
4392	heme oxygenase (decyclizing) activity	33.33	0.045
70700	BMP receptor binding	33.33	0.045
46875	ephrin receptor binding	33.33	0.045
8329	pattern recognition receptor activity	33.33	0.045
1965	G-protein alpha-subunit binding	33.33	0.045
30675	Rac GTPase activator activity	33.33	0.045
5547	phosphatidylinositol-3,4,5-trisphosphate binding	33.33	0.045
16820	hydrolase activity, acting on acid anhydrides, catalyzing transmembrane movement of substances	5.56	0.048

[a] Indicates the percent of genes in that GO category that were differentially expressed within our dataset.
