# Supplementary material for: A transcriptomal analysis of bovine oviductal epithelial cells collected during the follicular phase versus the luteal phase of the estrous cycle
Source: Reprod Biol Endocrinol. 2015 Aug 5;13:84. doi: 10.1186/s12958-015-0077-1 (PMC4524109; doi:10.1186/s12958-015-0077-1)
Supplement: Additional file 7: — Supplementary Table 5. Canonical pathway analysis of up-regulated DEGS within epithelial cells of the ampulla in the follicular versus luteal phase groups (P < 0.05). [file 12958_2015_77_MOESM7_ESM.pdf]

Supplementary Table 5. Canonical pathway analysis of up-regulated DEGS within epithelial cells of the ampulla in the follicular versus luteal phase groups (P < 0.05).

Canonical Pathways	P-value	Differentially expressed genes within pathway
Superpathway of Cholesterol Biosynthesis	< 0.001	MVD,SQLE,NSDHL,ACAT2,IDI1,MVK,MSMO1,TM7SF2,FDPS,FDFT1,DHCR7,EBP,DHCR24,LSS,HMGCR,HMGCS1,CYP51A1
Cholesterol Biosynthesis I	< 0.001	SQLE,FDFT1,EBP,NSDHL,DHCR7,DHCR24,MSMO1,LSS,TM7SF2,CYP51A1
Cholesterol Biosynthesis II (via 24,25-dihydrolanosterol)	< 0.001	FDFT1,SQLE,EBP,NSDHL,DHCR7,DHCR24,MSMO1,LSS,TM7SF2,CYP51A1
Cholesterol Biosynthesis III (via Desmosterol)	< 0.001	SQLE,FDFT1,EBP,NSDHL,DHCR7,DHCR24,MSMO1,LSS,TM7SF2,CYP51A1
Oxidative Phosphorylation	< 0.001	NDUFA4,ATP5J,COX7B,NDUFA7,UQCR11,CYCS,COX7A1,NDUFA13,NDUFB11,NDUFS8,NDUFA11,ATP5G1,COX5A,NDUFS6,NDUFA3,UQCRQ,ATP5G3
Mitochondrial Dysfunction	< 0.001	NDUFA4,HSD17B10,ATP5J,COX7B,NDUFA7,CASP3,UQCR11,CYCS,DHODH,COX7A1,NDUFA13,GSR,NDUFB11,NDUFS8,ATP5G1,NDUFA11,COX5A,NDUFS6,NDUFA3,GPX4,ATP5G3,UQCRQ
Colanic Acid Building Blocks Biosynthesis	< 0.001	TSTA3,UGDH,GMPPA,GMDS,GALT,GALE,PMM2
Endoplasmic Reticulum Stress Pathway	< 0.001	CALR,HSP90B1,CASP3,DDIT3,DNAJC3,ATF4,HSPA5,EIF2AK3
Superpathway of Geranylgeranyldiphosphate Biosynthesis I (via Mevalonate)	< 0.001	MVD,FDPS,ACAT2,IDI1,MVK,HMGCR,HMGCS1
Mevalonate Pathway I	< 0.001	MVD,ACAT2,IDI1,MVK,HMGCR,HMGCS1
Dolichyl-diphosphooligosaccharide Biosynthesis	< 0.001	ALG3,ALG5,DPM2,DPAGT1,DPM3
Zymosterol Biosynthesis	< 0.001	NSDHL,MSMO1,TM7SF2,CYP51A1
Estrogen-mediated S-phase Entry	< 0.001	CCNA2,CCNE2,E2F4,CCNE1,E2F1,CDK1,CDC25A
Superpathway of Serine and Glycine Biosynthesis I	< 0.001	PSAT1,PSPH,PHGDH,SHMT2
Cell Cycle Control of Chromosomal Replication	< 0.001	MCM5,MCM3,MCM6,MCM2,CDC6,DBF4,ORC1
Breast Cancer Regulation by Stathmin1	< 0.001	TUBA1B,CCNE2,TUBB3,E2F4,TUBG1,TUBA4A,GNG13,GNG3,CDK1,TUBB2B,GNG10,PIK3R3,SHC1,STMN1,CCNE1,TUBA8,TUBB6,E2F1,ITPR3,PRKACA,PRKCB
tRNA Charging	< 0.001	YARS,GARS,TARS,AARS,SARS,VARS,MARS,FARSA
ATM Signaling	0.001	CDC25C,SMC2,FANCD2,ATF4,BID,CREB3L4,BLM,CDK1,CDC25A,CHEK1
Oleate Biosynthesis II (Animals)	0.001	SCD,FADS2,PTPRT,FADS1

Mismatch Repair in Eukaryotes	0.001	PCNA,MSH2,RFC4,SLC19A1,EXO1
Remodeling of Epithelial Adherens Junctions	0.001	TUBA1B,TUBB3,TUBB6,TUBA8,ACTA2,ARPC5L,TUBG1,TUBA4A,ARPC3,TUBB2B
NRF2-mediated Oxidative Stress Response	0.001	GSTM1,PPIB,PRDX1,DNAJC3,HERPUD1,DNAJB2,DNAJB9,PIK3R3,GSR,ERP29,ACTA2,SCARB1,DNAJB11,VCP,ATF4,MAP2K3,EIF2AK3,DNAJB5,PRKCB
Chondroitin and Dermatan Biosynthesis	0.002	CHSY3,CHPF,CHSY1
Serine Biosynthesis	0.002	PSAT1,PSPH,PHGDH
Role of CHK Proteins in Cell Cycle Checkpoint Control	0.002	PCNA,CDC25C,E2F4,RFC4,E2F1,SLC19A1,CDK1,CDC25A,CHEK1
Hereditary Breast Cancer Signaling	0.002	CDC25C,TUBG1,CDK1,CHEK1,POLR2G,PIK3R3,FANCB,FANCD2,MSH2,RFC4,POLR2E,E2F1,SLC19A1,BLM
UDP-N-acetyl-D-glucosamine Biosynthesis II	0.003	GNPNAT1,GFPT1,PGM3
Epoxysqualene Biosynthesis	0.003	SQLE,FDFT1
GDP-L-fucose Biosynthesis I (from GDP-D-mannose)	0.003	TSTA3,GMDS
Role of BRCA1 in DNA Damage Response	0.004	FANCB,E2F4,FANCD2,MSH2,RFC4,E2F1,SLC19A1,BLM,CHEK1
Cell Cycle: G2/M DNA Damage Checkpoint Regulation	0.005	CDC25C,CKS2,CKS1B,BORA,AURKA,CDK1,CHEK1
Ketolysis	0.005	BDH1,ACAT2,OXCT1
Mitotic Roles of Polo-Like Kinase	0.006	CDC25C,HSP90B1,PLK4,CDC20,PRC1,CDK1,KIF11,CDC25A
γ-linolenate Biosynthesis II (Animals)	0.007	ACSL3,FADS2,SLC27A4,FADS1
Stearate Biosynthesis I (Animals)	0.008	ACSL3,DHCR24,FASN,ELOVL1,SLC27A4,ELOVL6
Trans, trans-farnesyl Diphosphate Biosynthesis	0.009	FDPS,IDI1
Granzyme B Signaling	0.010	CASP3,CYCS,BID,LMNB2
Hypoxia Signaling in the Cardiovascular System	0.010	P4HB,HSP90B1,UBE2J1,UBE2M,ATF4,CREB3L4,LDHA,UBE2C
UDP-N-acetyl-D-galactosamine Biosynthesis II	0.011	GNPNAT1,PGM3,GALE
14-3-3-mediated Signaling	0.013	PIK3R3,TUBA1B,TUBB3,TUBB6,TUBA8,PDIA3,TUBG1,TUBA4A,GFAP,BAX,TUBB2B,PRKCB
Ketogenesis	0.015	BDH1,ACAT2,HMGCS1
GADD45 Signaling	0.015	PCNA,CCNE2,CCNE1,CDK1
Gap Junction	0.015	TUBA1B,TUBB3,CSNK1G2,PDIA3,TUBG1,TUBA4A,TUBB2B,PIK3R3

Signaling		,TUBB6,TUBA8,ACTA2,ITPR3,PRKACA,PRKCB
dTMP De Novo Biosynthesis	0.017	DHFR,SHMT2
CCR3 Signaling in Eosinophils	0.023	PIK3R3,PLA2G4D,CFL1,PAK3,PLA2G10,ITPR3,GNG13,PLA2G4F,GNG3,GNG10,PRKCB
Germ Cell-Sertoli Cell Junction Signaling	0.025	TUBA1B,TUBB3,CFL1,RHOC,TUBG1,TUBA4A,TUBB2B,PIK3R3,MAP3K12,TUBB6,TUBA8,PAK3,ACTA2,MAP2K3
Antigen Presentation Pathway	0.025	CALR,PSMB5,PDIA3,PSMB6
CMP-N-acetylneuraminate Biosynthesis I (Eukaryotes)	0.027	GNE,NANS
Galactose Degradation I (Leloir Pathway)	0.027	GALT,GALE
Acetate Conversion to Acetyl-CoA	0.027	ACSL3,ACSS2
RAN Signaling	0.031	KPNB1,KPNA2,RAN
Isoleucine Degradation I	0.031	HSD17B10,ACAT2,BCAT2
Glycolysis I	0.035	PGAM1,FBP1,PFKL,ALDOC
Gluconeogenesis I	0.035	PGAM1,FBP1,MDH1,ALDOC
Myc Mediated Apoptosis Signaling	0.035	PIK3R3,SHC1,IGF1,CASP3,CYCS,BID,BAX
GDP-glucose Biosynthesis	0.039	PGM3,PGM1
Aspartate Degradation II	0.039	GOT1,MDH1
GDP-mannose Biosynthesis	0.039	GMPPA,PMM2
Tumoricidal Function of Hepatic Natural Killer Cells	0.040	CASP3,CYCS,BID,BAX
Huntington's Disease Signaling	0.040	ATP5J,YKT6,CASP3,CYCS,GNG13,GNG3,CREB3L4,BAX,HSPA5,GNG10,POLR2G,PIK3R3,SHC1,ARFIP2,IGF1,POLR2E,ATF4,PRKCB
DNA damage-induced 14-3-3σ Signaling	0.046	CCNE2,CCNE1,CDK1
p53 Signaling	0.048	PIK3R3,PCNA,SNAI2,E2F1,BAX,TNFRSF10A,BIRC5,CHEK1,TP53I3
Docosahexaenoic Acid (DHA) Signaling	0.049	PIK3R3,CASP3,CYCS,BID,BAX
