# Supplementary material for: A transcriptomal analysis of bovine oviductal epithelial cells collected during the follicular phase versus the luteal phase of the estrous cycle
Source: Reprod Biol Endocrinol. 2015 Aug 5;13:84. doi: 10.1186/s12958-015-0077-1 (PMC4524109; doi:10.1186/s12958-015-0077-1)

Supplementary Figure 3. Top 6 Canonical pathways from up- and down-regulated differentially expressed genes within epithelial cells of the ampulla in the follicular versus luteal phase. Ingenuity Pathway Analysis software was used to determine significant pathways based on the number of significant genes expressed within the pathway using Fischer's Exact test ($P < 0.05$).

A) Ampulla: Up-regulated pathways in the follicular versus luteal phases

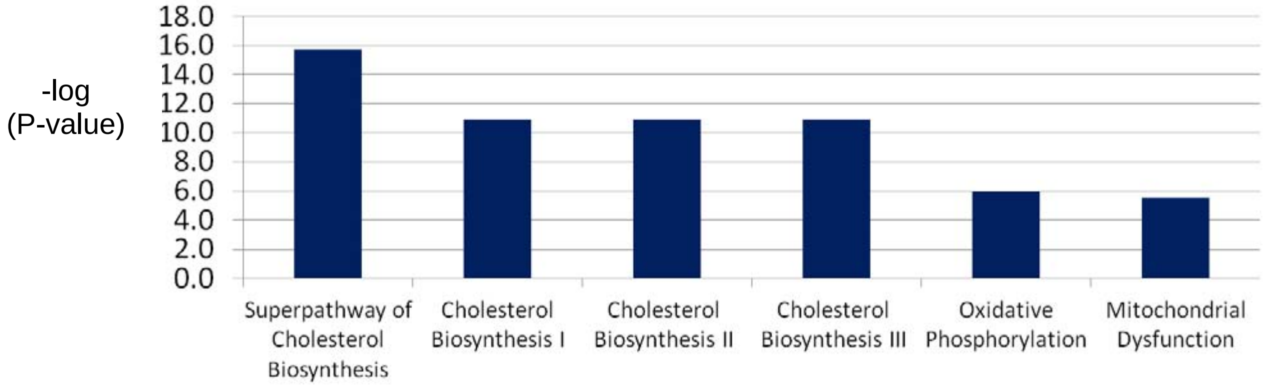

B) Ampulla: Down-regulated pathways in the follicular versus luteal phases

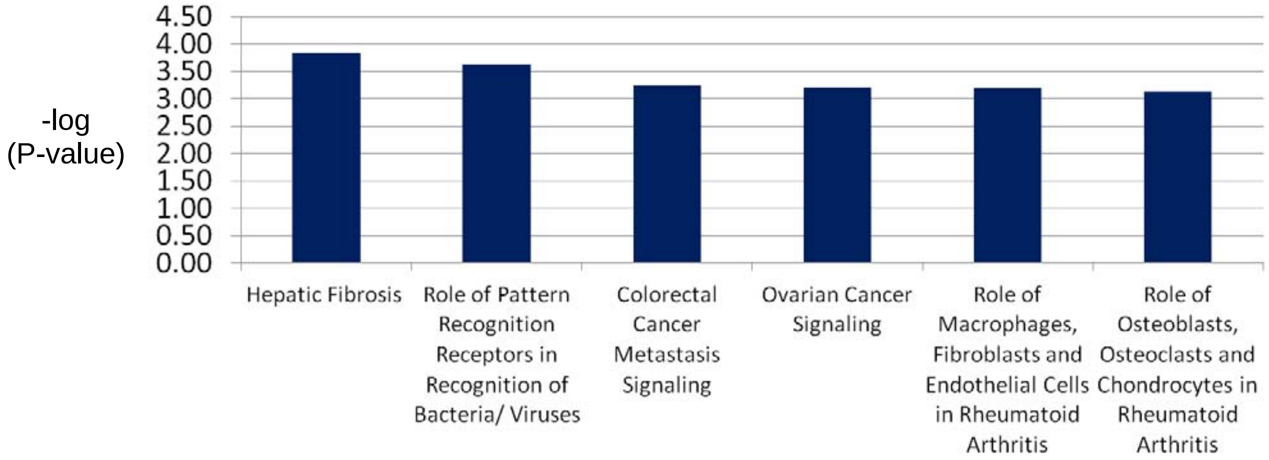

Supplement: Additional file 8: — Supplementary Figure 3. Top 6 Canonical pathways from up- and down-regulated differentially expressed genes within epithelial cells of the ampulla in the follicular versus luteal phase. Ingenuity Pathway Analysis software was used to determine significant pathways based on the number of significant genes expressed within the pathway using Fischer’s Exact test (P <0.05). A) Ampulla: Up-regulated pathways in the follicular versus luteal phases. B) Ampulla: Down-regulated pathways in the follicular versus luteal phases. [file 12958_2015_77_MOESM8_ESM.pdf]
