# Supplementary material for: A transcriptomal analysis of bovine oviductal epithelial cells collected during the follicular phase versus the luteal phase of the estrous cycle
Source: Reprod Biol Endocrinol. 2015 Aug 5;13:84. doi: 10.1186/s12958-015-0077-1 (PMC4524109; doi:10.1186/s12958-015-0077-1)
Supplement: Additional file 9: — Supplementary Table 6. Canonical pathway analysis of down-regulated DEGS within epithelial cells of the ampulla in the follicular versus luteal phase groups (P < 0.05). [file 12958_2015_77_MOESM9_ESM.pdf]

Supplementary Table 6. Canonical pathway analysis of down-regulated DEGS within epithelial cells of the ampulla in the follicular versus luteal phase groups (P < 0.05).

Canonical Pathways	P-value	Differentially expressed genes within pathway
Hepatic Fibrosis / Hepatic Stellate Cell Activation	< 0.001	VCAM1,IL1RAPL2,SMAD3,TNFSF10,PDGFC,FAS,BCL2,TGFBR2,MET,CSF1,HGF,IGFBP3,FIGF,TNFSF13B,EGFR
Role of Pattern Recognition Receptors in Recognition of Bacteria and Viruses	< 0.001	CLEC7A,OAS2,DDX58,TLR6,TLR8,TLR7,C1QA,C1QB,RNASEL,C3AR1,PRKD1
Colorectal Cancer Metastasis Signaling	0.001	MMP7,SMAD3,TLR8,PDGFC,CCND1,TGFBR2,FOS,ARRB1,FZD4,TLR6,SMO,TLR7,FIGF,FNBP1,EGFR,FZD7
Ovarian Cancer Signaling	0.001	MMP7,ARRB1,FZD4,FGF9,SMO,FIGF,CCND1,PDGFC,FZD7,EGFR,BCL2
Role of Macrophages, Fibroblasts and Endothelial Cells in Rheumatoid Arthritis	0.001	VCAM1,IL1RAPL2,IL15,TLR8,PDGFC,CCND1,FOS,FZD4,NFAT5,CSF1,TLR6,SMO,TLR7,FIGF,TRAF5,TNFSF13B,PRKD1,FZD7
Role of Osteoblasts, Osteoclasts and Chondrocytes in Rheumatoid Arthritis	0.001	NAIP,BMP4,IL1RAPL2,PTK2B,MAP3K5,CSF1R,BCL2,FOS,NFAT5,FZD4,FOXO1,CSF1,SMO,TRAF5,FZD7
Complement System	0.001	SERPING1,CD59,C1QA,C1QB,C3AR1
Molecular Mechanisms of Cancer	0.002	NAIP,BMP4,SMAD3,PTCH1,CDK6,MAP3K5,BMPR1B,CCND1,FAS,BCL2,TGFBR2,FOS,FZD4,FOXO1,SUFU,RASGRP1,SMO,FNBP1,PRKD1,FZD7
Granulocyte Adhesion and Diapedesis	0.002	HRH1,CLDN10,SELL,MMP7,VCAM1,IL1RAPL2,CLDN8,CLDN1,CLDN16,CXCL2,SELPLG
Leukocyte Extravasation Signaling	0.002	CLDN10,TIMP3,MMP7,VCAM1,PTK2B,SELPLG,CLDN8,CLDN1,CLDN16,VAV3,RASGRP1,NCF2,PRKD1
Macropinocytosis Signaling	0.003	MET,CSF1,HGF,ITGB6,CSF1R,PDGFC,PRKD1
Altered T Cell and B Cell Signaling in Rheumatoid Arthritis	0.004	CSF1,TLR6,IL15,TLR8,TLR7,TNFSF13B,FAS
Spermine and Spermidine Degradation I	0.005	SMOX,SAT1
IL-8 Signaling	0.005	HMOX1,FOS,VCAM1,PTK2B,NCF2,FIGF,CCND1,PDGFC,FNBP1,PRKD1,EGFR,BCL2
B Cell Receptor Signaling	0.006	BLNK,MAP3K15,NFAT5,FOXO1,PTK2B,DAPP1,PAG1,VAV3,EGR1,MAP3K5,BCL6
HER-2 Signaling in Breast Cancer	0.006	FOXO1,CDK6,MAP3K5,ITGB6,CCND1,PRKD1,EGFR
Bladder Cancer Signaling	0.006	DAPK1,MMP7,FGF9,FIGF,CCND1,PDGFC,EGFR
Basal Cell Carcinoma Signaling	0.009	BMP4,FZD4,SUFU,PTCH1,SMO,FZD7
Crosstalk between Dendritic Cells and Natural Killer Cells	0.009	KLRD1,TYROBP,IL15,TLR7,TNFSF10,FAS

Agranulocyte Adhesion and Diapedesis	0.009	HRH1,CLDN10,SELL,MMP7,VCAM1,CLDN8,CLDN1,CLDN16,CXCL2,SELPLG
Pancreatic Adenocarcinoma Signaling	0.009	TGFBR2,HMOX1,SMAD3,FIGF,CCND1,PDGFC,EGFR,BCL2
TGF-β Signaling	0.011	TGFBR2,FOS,BMP4,SMAD3,VDR,BMPR1B,BCL2
Factors Promoting Cardiogenesis in Vertebrates	0.012	TGFBR2,BMP4,FZD4,SMO,BMPR1B,PRKD1,FZD7
Death Receptor Signaling	0.012	NAIP,PARP8,TNFSF10,MAP3K5,PARP9,FAS,BCL2
NF-κB Signaling	0.015	TGFBR2,BMP4,TLR6,TLR8,TLR7,TRAF5,INSR,BMPR1B,TNFSF13B,EGFR
Natural Killer Cell Signaling	0.016	KLRD1,TYROBP,VAV3,KLRB1,CD244,PRKD1,KLRC1
STAT3 Pathway	0.017	TGFBR2,PIM1,INSR,BMPR1B,EGFR,BCL2
PTEN Signaling	0.017	TGFBR2,FOXO4,FOXO1,INSR,BMPR1B,CCND1,EGFR,BCL2
Communication between Innate and Adaptive Immune Cells	0.018	TLR6,IL15,TLR8,TLR7,TNFSF13B
RAR Activation	0.018	FOS,DUSP1,SDR16C5,SMAD3,PNRC1,IGFBP3,MAP3K5,ZBTB16,PNPLA4,PRKD1
Glioblastoma Multiforme Signaling	0.020	FZD4,FOXO1,CDK6,SMO,CCND1,PDGFC,FNBP1,FZD7,EGFR
Regulation of the Epithelial-Mesenchymal Transition Pathway	0.022	MET,TGFBR2,FZD4,EGR1,SMAD3,HGF,FGF9,SMO,FZD7,EGFR
B Cell Activating Factor Signaling	0.022	FOS,NFAT5,TRAF5,TNFSF13B
IL-15 Production	0.022	PTK2B,IL15,IRF1
Human Embryonic Stem Cell Pluripotency	0.024	TGFBR2,BMP4,FZD4,FOXO1,SMAD3,SMO,PDGFC,FZD7
HGF Signaling	0.025	MET,MAP3K15,FOS,HGF,MAP3K5,CCND1,PRKD1
Retinoic acid Mediated Apoptosis Signaling	0.030	PARP8,TNFSF10,PARP9,IRF1
Thio-molybdenum Cofactor Biosynthesis	0.030	MOCOS
p38 MAPK Signaling	0.032	TGFBR2,RPS6KA6,IL1RAPL2,DUSP1,DUSP10,MAP3K5,FAS
Hematopoiesis from Multipotent Stem Cells	0.033	CSF1,IL15
Amyotrophic Lateral Sclerosis Signaling	0.034	NAIP,CAPN6,GRID1,FIGF,PDGFC,BCL2
Role of Tissue Factor in Cancer	0.034	RPS6KA6,ARRB1,PTK2B,CSF1,EGR1,CYR61,EGFR
Sertoli Cell-Sertoli Cell Junction Signaling	0.038	MAP3K15,CLDN10,CLDN8,CLDN1,CLDN16,PPAP2B,CGN,CAV1,MAP3K5
Sonic Hedgehog Signaling	0.040	SUFU,PTCH1,SMO
Interferon Signaling	0.048	IRF9,IRF1,BCL2

Glioma Signaling	0.049	CAMK1D,CDK6,CCND1,PDGFC,PRKD1,EGFR
T Cell Receptor Signaling	0.049	FOS,NFAT5,GRAP2,PAG1,VAV3,RASGRP1
