# Supplementary material for: A transcriptomal analysis of bovine oviductal epithelial cells collected during the follicular phase versus the luteal phase of the estrous cycle
Source: Reprod Biol Endocrinol. 2015 Aug 5;13:84. doi: 10.1186/s12958-015-0077-1 (PMC4524109; doi:10.1186/s12958-015-0077-1)
Supplement: Additional file 10: — Supplementary Table 7. Enriched gene ontology classifications from up-regulated DEGS within epithelial cells of the isthmus in follicular versus luteal phase groups (P < 0.05). [file 12958_2015_77_MOESM10_ESM.pdf]

Supplementary Table 7. Enriched gene ontology classifications from up-regulated DEGS within epithelial cells of the isthmus in follicular versus luteal phase groups (P < 0.05).

Biological Processes			
**GO ID**	**Description**	**% of significant genes falling into category [a]**	**Enrichment P-value**
6457	protein folding	15.03	< 0.001
7049	cell cycle	13.26	< 0.001
51301	cell division	15.00	< 0.001
7067	mitosis	15.63	< 0.001
22900	electron transport chain	15.91	< 0.001
6810	transport	8.77	< 0.001
6695	cholesterol biosynthetic process	35.29	< 0.001
18208	peptidyl-proline modification	45.45	< 0.001
413	protein peptidyl-prolyl isomerization	45.45	< 0.001
18279	protein N-linked glycosylation via asparagine	66.67	< 0.001
6687	glycosphingolipid metabolic process	100.00	< 0.001
6986	response to unfolded protein	38.46	< 0.001
10629	negative regulation of gene expression	38.46	< 0.001
6662	glycerol ether metabolic process	31.25	< 0.001
32981	mitochondrial respiratory chain complex I assembly	44.44	< 0.001
30970	retrograde protein transport, ER to cytosol	75.00	< 0.001
90398	cellular senescence	75.00	< 0.001
6486	protein glycosylation	15.25	< 0.001
8299	isoprenoid biosynthetic process	29.41	< 0.001
30433	ER-associated protein catabolic process	40.00	< 0.001
51437	positive regulation of ubiquitin-protein ligase activity involved in mitotic cell cycle	18.42	< 0.001
31145	anaphase-promoting complex-dependent proteasomal ubiquitin-dependent protein catabolic process	17.95	< 0.001
86	G2/M transition of mitotic cell cycle	36.36	< 0.001
8652	cellular amino acid biosynthetic process	25.00	< 0.001
9314	response to radiation	50.00	0.001
7059	chromosome segregation	22.73	0.001
30968	endoplasmic reticulum unfolded protein response	30.77	0.001
51436	negative regulation of ubiquitin-protein ligase activity involved in mitotic cell cycle	17.65	0.001
15986	ATP synthesis coupled proton transport	21.74	0.001
6260	DNA replication	11.11	0.001
45665	negative regulation of neuron differentiation	37.50	0.002
51258	protein polymerization	15.00	0.002
6465	signal peptide processing	33.33	0.003
8285	negative regulation of cell proliferation	10.96	0.003

35234	germ cell programmed cell death	66.67	0.003
6563	L-serine metabolic process	66.67	0.003
6275	regulation of DNA replication	66.67	0.003
34501	protein localization to kinetochore	66.67	0.003
43103	hypoxanthine salvage	66.67	0.003
6641	triglyceride metabolic process	30.00	0.004
43123	positive regulation of I-kappaB kinase/NF-kappaB cascade	11.67	0.004
6633	fatty acid biosynthetic process	12.77	0.005
44262	cellular carbohydrate metabolic process	27.27	0.005
45454	cell redox homeostasis	10.94	0.006
1666	response to hypoxia	14.29	0.006
7224	smoothened signaling pathway	50.00	0.007
71569	protein ufmylation	50.00	0.007
460	maturation of 5.8S rRNA	50.00	0.007
45445	myoblast differentiation	50.00	0.007
48146	positive regulation of fibroblast proliferation	25.00	0.007
45931	positive regulation of mitotic cell cycle	25.00	0.007
6270	DNA-dependent DNA replication initiation	25.00	0.007
51603	proteolysis involved in cellular protein catabolic process	17.39	0.007
9636	response to toxin	23.08	0.009
45740	positive regulation of DNA replication	21.43	0.011
10977	negative regulation of neuron projection development	40.00	0.011
50768	negative regulation of neurogenesis	40.00	0.011
60766	negative regulation of androgen receptor signaling pathway	40.00	0.011
48541	Peyer's patch development	40.00	0.011
6950	response to stress	12.20	0.012
7080	mitotic metaphase plate congression	33.33	0.016
16254	preassembly of GPI anchor in ER membrane	33.33	0.016
16925	protein sumoylation	33.33	0.016
2088	lens development in camera-type eye	33.33	0.016
6166	purine ribonucleoside salvage	33.33	0.016
30879	mammary gland development	33.33	0.016
7420	brain development	13.79	0.016
6979	response to oxidative stress	10.64	0.021
42110	T cell activation	28.57	0.022
71363	cellular response to growth factor stimulus	28.57	0.022
40008	regulation of growth	15.79	0.025
6974	response to DNA damage stimulus	11.76	0.027
72593	reactive oxygen species metabolic process	25.00	0.028
1975	response to amphetamine	25.00	0.028
19370	leukotriene biosynthetic process	25.00	0.028

6297	nucleotide-excision repair, DNA gap filling	25.00	0.028
45668	negative regulation of osteoblast differentiation	25.00	0.028
42326	negative regulation of phosphorylation	25.00	0.028
7265	Ras protein signal transduction	15.00	0.029
7399	nervous system development	9.62	0.031
42632	cholesterol homeostasis	14.29	0.033
7411	axon guidance	14.29	0.033
9968	negative regulation of signal transduction	14.29	0.033
1836	release of cytochrome c from mitochondria	22.22	0.035
9396	folic acid-containing compound biosynthetic process	22.22	0.035
8277	regulation of G-protein coupled receptor protein signaling pathway	22.22	0.035
70328	triglyceride homeostasis	22.22	0.035
33344	cholesterol efflux	22.22	0.035
45840	positive regulation of mitosis	22.22	0.035
8584	male gonad development	13.64	0.037
30162	regulation of proteolysis	13.64	0.037
7050	cell cycle arrest	10.53	0.039
10951	negative regulation of endopeptidase activity	13.04	0.042
6099	tricarboxylic acid cycle	13.04	0.042
1974	blood vessel remodeling	20.00	0.043
6915	apoptosis	6.33	0.043
10628	positive regulation of gene expression	12.50	0.046
**cellular Components**			
5783	endoplasmic reticulum	19.30	< 0.001
5789	endoplasmic reticulum membrane	17.21	< 0.001
5739	mitochondrion	9.74	< 0.001
5737	cytoplasm	6.79	< 0.001
5788	endoplasmic reticulum lumen	37.78	< 0.001
16020	membrane	5.81	< 0.001
5743	mitochondrial inner membrane	13.39	< 0.001
5829	cytosol	8.83	< 0.001
42470	melanosome	23.64	< 0.001
5793	endoplasmic reticulum-Golgi intermediate compartment	41.18	< 0.001
5634	nucleus	4.96	< 0.001
8250	oligosaccharyltransferase complex	80.00	< 0.001
70469	respiratory chain	17.19	< 0.001
5654	nucleoplasm	9.23	< 0.001
5747	mitochondrial respiratory chain complex I	20.00	< 0.001
30176	integral to endoplasmic reticulum membrane	21.43	< 0.001

5856	cytoskeleton	7.16	< 0.001
5794	Golgi apparatus	6.87	0.001
15630	microtubule cytoskeleton	22.73	0.001
30173	integral to Golgi membrane	17.65	0.001
45263	proton-transporting ATP synthase complex, coupling factor F(o)	42.86	0.001
307	cyclin-dependent protein kinase holoenzyme complex	42.86	0.001
5576	extracellular region	5.26	0.001
777	condensed chromosome kinetochore	20.00	0.001
5604	basement membrane	20.00	0.001
5730	nucleolus	7.66	0.002
5874	microtubule	8.70	0.003
5792	microsome	11.27	0.003
139	Golgi membrane	8.87	0.003
5764	lysosome	10.67	0.004
5787	signal peptidase complex	50.00	0.007
5832	chaperonin-containing T-complex	50.00	0.007
785	chromatin	16.67	0.008
5886	plasma membrane	4.77	0.010
5689	U12-type spliceosomal complex	21.43	0.011
5876	spindle microtubule	21.43	0.011
5640	nuclear outer membrane	33.33	0.016
30659	cytoplasmic vesicle membrane	13.79	0.016
5625	soluble fraction	11.36	0.016
16021	integral to membrane	3.98	0.016
5578	proteinaceous extracellular matrix	7.76	0.017
5813	centrosome	9.52	0.020
43234	protein complex	10.64	0.021
178	exosome (RNase complex)	28.57	0.022
42555	MCM complex	28.57	0.022
31410	cytoplasmic vesicle	7.32	0.024
5912	adherens junction	25.00	0.028
5657	replication fork	22.22	0.035
31966	mitochondrial membrane	10.81	0.036
30496	midbody	10.81	0.036
775	chromosome, centromeric region	13.64	0.037
5694	chromosome	6.21	0.039

48471	perinuclear region of cytoplasm	6.67	0.041
5746	mitochondrial respiratory chain	20.00	0.043
276	mitochondrial proton-transporting ATP synthase complex, coupling factor F(o)	20.00	0.043
34361	very-low-density lipoprotein particle	20.00	0.043
5811	lipid particle	20.00	0.043
	**Molecular Functions**		
16740	transferase activity	8.66	< 0.001
3755	peptidyl-prolyl cis-trans isomerase activity	20.45	< 0.001
5528	FK506 binding	45.45	< 0.001
3756	protein disulfide isomerase activity	66.67	< 0.001
16853	isomerase activity	13.13	< 0.001
4579	dolichyl-diphosphooligosaccharide-protein glycotransferase activity	50.00	< 0.001
51082	unfolded protein binding	11.34	< 0.001
42277	peptide binding	50.00	0.001
4129	cytochrome-c oxidase activity	16.22	0.001
15035	protein disulfide oxidoreductase activity	17.24	0.003
51787	misfolded protein binding	66.67	0.003
4768	stearoyl-CoA 9-desaturase activity	66.67	0.003
16671	oxidoreductase activity, acting on a sulfur group of donors, disulfide as acceptor	66.67	0.003
16874	ligase activity	7.98	0.004
16491	oxidoreductase activity	5.85	0.004
30332	cyclin binding	50.00	0.007
49	tRNA binding	25.00	0.007
43130	ubiquitin binding	25.00	0.007
16787	hydrolase activity	5.19	0.009
4656	procollagen-proline 4-dioxygenase activity	40.00	0.011
4488	methylenetetrahydrofolate dehydrogenase (NADP+) activity	40.00	0.011
4862	cAMP-dependent protein kinase inhibitor activity	40.00	0.011
15078	hydrogen ion transmembrane transporter activity	15.38	0.011
31625	ubiquitin protein ligase binding	12.20	0.012
42802	identical protein binding	7.63	0.014

5506	iron ion binding	9.21	0.015
4280<br>3	protein homodimerization activity	6.98	0.015
5385	zinc ion transmembrane transporter activity	33.33	0.016
5069<br>9	WW domain binding	33.33	0.016
4620	phospholipase activity	33.33	0.016
5149	interleukin-1 receptor binding	33.33	0.016
8121	ubiquinol-cytochrome-c reductase activity	28.57	0.022
5192<br>0	peroxiredoxin activity	28.57	0.022
5159	insulin-like growth factor receptor binding	28.57	0.022
8137	NADH dehydrogenase (ubiquinone) activity	12.50	0.022
9055	electron carrier activity	6.71	0.024
8373	sialyltransferase activity	15.79	0.025
166	nucleotide binding	4.24	0.027
5068<br>1	androgen receptor binding	25.00	0.028
1671<br>7	oxidoreductase activity, acting on paired donors, with oxidation of a pair of donors resulting in the reduction of molecular oxygen to two molecules of water	25.00	0.028
5075<br>0	low-density lipoprotein particle receptor binding	25.00	0.028
4298	threonine-type endopeptidase activity	15.00	0.029
4239<br>3	histone binding	14.29	0.033
4698<br>2	protein heterodimerization activity	8.33	0.035
5198	structural molecule activity	5.80	0.043
5524	ATP binding	4.21	0.046

[a] Indicates the percent of genes in that GO category that were differentially expressed within our dataset.
