# Supplementary material for: A transcriptomal analysis of bovine oviductal epithelial cells collected during the follicular phase versus the luteal phase of the estrous cycle
Source: Reprod Biol Endocrinol. 2015 Aug 5;13:84. doi: 10.1186/s12958-015-0077-1 (PMC4524109; doi:10.1186/s12958-015-0077-1)
Supplement: Additional file 11: — Supplementary Table 8. Enriched gene ontology classifications from down-regulated DEGS within epithelial cells of the isthmus in follicular versus luteal phase groups (P < 0.05). [file 12958_2015_77_MOESM11_ESM.pdf]

Supplementary Table 8. Enriched gene ontology classifications from down-regulated DEGS within epithelial cells of the isthmus in follicular versus luteal phase groups (P < 0.05).

Biological Processes

GO ID	Description	% of significant genes falling into category [a]	Enrichment P-value
40037	negative regulation of fibroblast growth factor receptor signaling pathway	100.00	< 0.001
16338	calcium-independent cell-cell adhesion	75.00	< 0.001
188	inactivation of MAPK activity	60.00	< 0.001
50873	brown fat cell differentiation	30.77	< 0.001
60395	SMAD protein signal transduction	50.00	< 0.001
7165	signal transduction	4.98	< 0.001
7605	sensory perception of sound	23.53	< 0.001
70374	positive regulation of ERK1 and ERK2 cascade	22.22	< 0.001
42493	response to drug	14.71	0.001
8285	negative regulation of cell proliferation	9.59	0.001
45087	innate immune response	10.53	0.001
46632	alpha-beta T cell differentiation	66.67	0.001
70373	negative regulation of ERK1 and ERK2 cascade	27.27	0.001
43407	negative regulation of MAP kinase activity	25.00	0.002
7275	multicellular organismal development	4.90	0.002
45893	positive regulation of transcription, DNA-dependent	7.41	0.002
48745	smooth muscle tissue development	50.00	0.002
46902	regulation of mitochondrial membrane permeability	50.00	0.002
45737	positive regulation of cyclin-dependent protein kinase activity	50.00	0.002
32956	regulation of actin cytoskeleton organization	50.00	0.002
61036	positive regulation of cartilage development	50.00	0.002
43507	positive regulation of JUN kinase activity	50.00	0.002
10759	positive regulation of macrophage chemotaxis	50.00	0.002
48286	lung alveolus development	21.43	0.003
6958	complement activation, classical pathway	21.43	0.003
30509	BMP signaling pathway	21.43	0.003
43065	positive regulation of apoptosis	10.64	0.003
6814	sodium ion transport	8.70	0.003
1525	angiogenesis	9.80	0.004
16567	protein ubiquitination	12.50	0.004
46580	negative regulation of Ras protein signal transduction	40.00	0.004
8016	regulation of heart contraction	40.00	0.004
2053	positive regulation of mesenchymal cell proliferation	40.00	0.004
70498	interleukin-1-mediated signaling pathway	40.00	0.004
10862	positive regulation of pathway-restricted SMAD protein phosphorylation	40.00	0.004

9887	organ morphogenesis	17.65	0.005
51897	positive regulation of protein kinase B signaling cascade	17.65	0.005
16055	Wnt receptor signaling pathway	9.09	0.005
7519	skeletal muscle tissue development	16.67	0.005
1759	organ induction	33.33	0.006
10575	positive regulation vascular endothelial growth factor production	33.33	0.006
6003	fructose 2,6-bisphosphate metabolic process	33.33	0.006
48754	branching morphogenesis of a tube	33.33	0.006
9790	embryo development	10.81	0.007
8104	protein localization	15.00	0.007
45766	positive regulation of angiogenesis	15.00	0.007
7254	JNK cascade	28.57	0.008
6865	amino acid transport	28.57	0.008
9611	response to wounding	28.57	0.008
7219	Notch signaling pathway	14.29	0.009
45892	negative regulation of transcription, DNA-dependent	6.74	0.010
186	activation of MAPKK activity	25.00	0.011
51090	regulation of sequence-specific DNA binding transcription factor activity	25.00	0.011
9086	methionine biosynthetic process	25.00	0.011
43537	negative regulation of blood vessel endothelial cell migration	25.00	0.011
9953	dorsal/ventral pattern formation	25.00	0.011
6917	induction of apoptosis	9.30	0.011
46777	protein autophosphorylation	12.50	0.012
122	negative regulation of transcription from RNA polymerase II promoter	7.35	0.013
6835	dicarboxylic acid transport	22.22	0.014
1890	placenta development	22.22	0.014
1836	release of cytochrome c from mitochondria	22.22	0.014
71456	cellular response to hypoxia	22.22	0.014
7595	lactation	22.22	0.014
40007	growth	12.00	0.014
42472	inner ear morphogenesis	20.00	0.017
46034	ATP metabolic process	20.00	0.017
1933	negative regulation of protein phosphorylation	20.00	0.017
45727	positive regulation of translation	18.18	0.020
1937	negative regulation of endothelial cell proliferation	18.18	0.020
51592	response to calcium ion	18.18	0.020
9749	response to glucose stimulus	18.18	0.020
46330	positive regulation of JNK cascade	18.18	0.020
30501	positive regulation of bone mineralization	18.18	0.020
7420	brain development	10.34	0.021

8360	regulation of cell shape	10.00	0.023
60070	canonical Wnt receptor signaling pathway	16.67	0.024
43066	negative regulation of apoptosis	6.10	0.026
30036	actin cytoskeleton organization	7.14	0.027
30218	erythrocyte differentiation	15.38	0.028
165	MAPKKK cascade	15.38	0.028
6821	chloride transport	8.82	0.032
7389	pattern specification process	14.29	0.032
209	protein polyubiquitination	14.29	0.032
45786	negative regulation of cell cycle	14.29	0.032
33138	positive regulation of peptidyl-serine phosphorylation	14.29	0.032
2755	MyD88-dependent toll-like receptor signaling pathway	8.57	0.034
45944	positive regulation of transcription from RNA polymerase II promoter	5.00	0.037
32436	positive regulation of proteasomal ubiquitin-dependent protein catabolic process	13.33	0.037
43433	negative regulation of sequence-specific DNA binding transcription factor activity	13.33	0.037
31323	regulation of cellular metabolic process	13.33	0.037
43154	negative regulation of caspase activity	13.33	0.037
8219	cell death	13.33	0.037
45669	positive regulation of osteoblast differentiation	13.33	0.037
6470	protein dephosphorylation	8.11	0.039
6469	negative regulation of protein kinase activity	12.50	0.041
9966	regulation of signal transduction	12.50	0.041
30307	positive regulation of cell growth	12.50	0.041
1568	blood vessel development	12.50	0.041
1938	positive regulation of endothelial cell proliferation	11.76	0.046
6874	cellular calcium ion homeostasis	11.76	0.046
**cellular Components**			
5737	cytoplasm	3.98	< 0.001
5886	plasma membrane	4.20	< 0.001
5829	cytosol	4.41	< 0.001
5576	extracellular region	3.51	0.002
5923	tight junction	11.63	0.002
31234	extrinsic to internal side of plasma membrane	18.75	0.004
16023	cytoplasmic membrane-bounded vesicle	10.26	0.008
31526	brush border membrane	28.57	0.008
307	cyclin-dependent protein kinase holoenzyme complex	28.57	0.008
16323	basolateral plasma membrane	10.00	0.009
16020	membrane	2.66	0.009
299	integral to membrane of membrane fraction	25.00	0.011
10494	stress granule	25.00	0.011

9897	external side of plasma membrane	9.09	0.012
16324	apical plasma membrane	8.70	0.014
5887	integral to plasma membrane	5.77	0.020
30027	lamellipodium	10.00	0.023
5834	heterotrimeric G-protein complex	10.00	0.023
5902	microvillus	16.67	0.024
5578	proteinaceous extracellular matrix	5.17	0.032
5901	caveola	14.29	0.032
5768	endosome	5.68	0.034
5765	lysosomal membrane	7.89	0.042
5925	focal adhesion	7.89	0.042
5615	extracellular space	3.48	0.044
5911	cell-cell junction	11.76	0.046
**Molecular Functions**			
42802	identical protein binding	8.40	< 0.001
4091	carboxylesterase activity	20.00	0.001
4719	protein-L-isoaspartate (D-aspartate) O-methyltransferase activity	66.67	0.001
17171	serine hydrolase activity	66.67	0.001
31821	metabotropic serotonin receptor binding	66.67	0.001
70700	BMP receptor binding	66.67	0.001
4888	transmembrane signaling receptor activity	11.63	0.002
16504	peptidase activator activity	50.00	0.002
46982	protein heterodimerization activity	8.33	0.003
4522	pancreatic ribonuclease activity	18.75	0.004
3873	6-phosphofructo-2-kinase activity	33.33	0.006
16787	hydrolase activity	3.56	0.006
3690	double-stranded DNA binding	15.79	0.006
30165	PDZ domain binding	15.00	0.007
43498	cell surface binding	28.57	0.008
4331	fructose-2,6-bisphosphate 2-phosphatase activity	28.57	0.008
42803	protein homodimerization activity	5.23	0.009
19899	enzyme binding	9.76	0.010
44212	transcription regulatory region DNA binding	13.64	0.010
17153	sodium:dicarboxylate symporter activity	25.00	0.011
5109	frizzled binding	25.00	0.011
4860	protein kinase inhibitor activity	25.00	0.011
42813	Wnt-activated receptor activity	25.00	0.011
8237	metallopeptidase activity	8.89	0.013
8301	DNA bending activity	22.22	0.014
8083	growth factor activity	5.47	0.016
19901	protein kinase binding	6.85	0.017

16805	dipeptidase activity	20.00	0.017
3707	steroid hormone receptor activity	7.84	0.020
15293	symporter activity	7.55	0.023
8134	transcription factor binding	7.55	0.023
17017	MAP kinase tyrosine/serine/threonine phosphatase activity	16.67	0.024
51020	GTPase binding	16.67	0.024
31683	G-protein beta/gamma-subunit complex binding	16.67	0.024
4437	inositol or phosphatidylinositol phosphatase activity	15.38	0.028
43499	eukaryotic cell surface binding	15.38	0.028
3705	sequence-specific distal enhancer binding RNA polymerase II transcription factor activity	14.29	0.032
8013	beta-catenin binding	14.29	0.032
17124	SH3 domain binding	8.11	0.039
5247	voltage-gated chloride channel activity	12.50	0.041
5484	SNAP receptor activity	12.50	0.041
16740	transferase activity	3.58	0.043
5178	integrin binding	11.76	0.046
19001	guanyl nucleotide binding	11.76	0.046
43565	sequence-specific DNA binding	3.30	0.047

[a] Indicates the percent of genes in that GO category that were differentially expressed within our dataset.
