# Supplementary material for: A transcriptomal analysis of bovine oviductal epithelial cells collected during the follicular phase versus the luteal phase of the estrous cycle
Source: Reprod Biol Endocrinol. 2015 Aug 5;13:84. doi: 10.1186/s12958-015-0077-1 (PMC4524109; doi:10.1186/s12958-015-0077-1)
Supplement: Additional file 12: — Supplementary Table 9. Canonical pathway analysis of up-regulated DEGS within epithelial cells of the isthmus in the follicular versus luteal phase groups (P < 0.05). [file 12958_2015_77_MOESM12_ESM.pdf]

Supplementary Table 9. Canonical pathway analysis of up-regulated DEGS within epithelial cells of the isthmus in the follicular versus luteal phase groups (P < 0.05).

**Canonical Pathways**	**P-value**	**Differentially expressed genes within pathway**
Oxidative Phosphorylation	< 0.001	NDUFA4,ATP5J,COX6B1,COX7B,UQCR11,NDUFB5,NDUFA13,COX11,NDUFS,NDUFA11,ATP5G1,NDUFS6,COX5A,NDUFA12,NDUFA3,UQCRQ,ATP5G3,NDUFS4
Mitochondrial Dysfunction	< 0.001	NDUFA4,HSD17B10,ATP5J,COX6B1,COX7B,UQCR11,PRDX5,CPT1B,NDUFB5,NDUFA13,GSR,COX11,NDUFS8,ATP5G1,NDUFA11,COX5A,NDUFS6,NDUFA12,NDUFA3,ATP5G3,UQCRQ,NDUFS4
Superpathway of Cholesterol Biosynthesis	< 0.001	FDPS,SQLE,NSDHL,ACAT2,IDI1,MVK,LSS,HMGCR,HMGCS1
Superpathway of Geranylgeranyldiphosphate Biosynthesis I (via Mevalonate)	< 0.001	FDPS,ACAT2,IDI1,MVK,HMGCR,HMGCS1
Mevalonate Pathway I	< 0.001	ACAT2,IDI1,MVK,HMGCR,HMGCS1
Cell Cycle Control of Chromosomal Replication	< 0.001	MCM3,MCM6,RPA3,MCM2,CDC6,DBF4,ORC1
Endoplasmic Reticulum Stress Pathway	< 0.001	CALR,HSP90B1,DDIT3,DNAJC3,HSPA5,EIF2AK3
Colanic Acid Building Blocks Biosynthesis	0.001	TSTA3,UGDH,GMPPA,GMDS,GALE
NRF2-mediated Oxidative Stress Response	0.001	GSTM1,PPIB,RRAS,PRDX1,DNAJC19,HSPB8,HERPUD1,DNAJC3,DNAJB9,GSR,ERP29,ACTA2,SCARB1,DNAJB11,CCT7,TXN,EIF2AK3,DNAJB5,PRKCB
Mitotic Roles of Polo-Like Kinase	0.001	CDC25C,HSP90B1,PLK4,CDC20,PPP2R2B,PRC1,PPP2R2C,CDK1,KIF11
Role of CHK Proteins in Cell Cycle Checkpoint Control	0.001	PCNA,CDC25C,RFC4,CDKN1A,PPP2R2B,SLC19A1,PPP2R2C,CDK1,CHEK1
Chondroitin and Dermatan Biosynthesis	0.001	CHSY3,CHPF,CHSY1
CMP-N-acetylneuraminate Biosynthesis I (Eukaryotes)	0.001	GNE,CMAS,NANS
GADD45 Signaling	0.002	PCNA,CCNE1,CCND2,CDKN1A,CDK1
UDP-N-acetyl-D-glucosamine Biosynthesis II	0.003	GNPNAT1,GFPT1,PGM3
Remodeling of Epithelial Adherens Junctions	0.003	TUBA1B,TUBB3,TUBA8,ACTA2,ARPC5L,TUBG1,TUBA4A,ARPC3,TUBB2B
GDP-L-fucose Biosynthesis I (from GDP-D-mannose)	0.003	TSTA3,GMDS
Superpathway of Serine and Glycine	0.004	PSAT1,PSPH,SHMT2

Biosynthesis I		
Ketolysis	0.004	BDH1,ACAT2,OXCT1
Estrogen-mediated S-phase Entry	0.006	CCNA2,CCNE1,CCNA1,CDKN1A,CDK1
Mismatch Repair in Eukaryotes	0.006	PCNA,RFC4,SLC19A1,EXO1
Protein Ubiquitination Pathway	0.008	PSMB3,UCHL3,CRYAB,PSMB5,CDC20,UBE2N,DNAJC19,HSPB8,PSMD6,DNAJC3,PSMA1,USP2,HSPA5,DNAJB9,PSMC5,HSP90B1,DNAJB11,HSPE1,PSMD14,DNAJB5,UBE2C
Spermidine Biosynthesis I	0.008	SRM,AMD1
Trans, trans-farnesyl Diphosphate Biosynthesis	0.008	FDPS,IDI1
Cyclins and Cell Cycle Regulation	0.009	CCNA2,CCNE1,CCND2,CCNA1,CDKN1A,PPP2R2B,TGFB3,PPP2R2C,CDK1
tRNA Charging	0.009	WARS,YARS,GARS,TARS,SARS,MARS
Aryl Hydrocarbon Receptor Signaling	0.009	GSTM1,BAX,CYP1B1,CHEK1,CCNA2,HSP90B1,CCNE1,ALDH1A1,CCND2,CCNA1,NEDD8,CDKN1A,TGFB3
Calcium Transport I	0.010	ATP2B1,ATP2C2,ATP2B2
UDP-N-acetyl-D-galactosamine Biosynthesis II	0.010	GNPNAT1,PGM3,GALE
Folate Transformations I	0.010	MTHFD1L,MTHFD2,SHMT2
Ketogenesis	0.013	BDH1,ACAT2,HMGCS1
Dolichyl-diphosphooligosaccharide Biosynthesis	0.013	ALG3,ALG5,DPAGT1
Cell Cycle: G2/M DNA Damage Checkpoint Regulation	0.015	CDC25C,CKS2,CDKN1A,BORA,CDK1,CHEK1
Sertoli Cell-Sertoli Cell Junction Signaling	0.015	TUBA1B,TUBB3,CLDN11,RRAS,TUBG1,TUBA4A,JAM2,TUBB2B,MPP6,MAP3K12,TUBA8,ACTA2,JAM3,TGFB3,PVRL1
Adenine and Adenosine Salvage III	0.015	ADA,HPRT1
Cholesterol Biosynthesis I	0.022	SQLE,NSDHL,LSS
Cholesterol Biosynthesis II (via 24,25-dihydrolanosterol)	0.022	SQLE,NSDHL,LSS
Cholesterol Biosynthesis III (via Desmosterol)	0.022	SQLE,NSDHL,LSS
14-3-3-mediated Signaling	0.023	TUBA1B,TUBB3,PLCE1,TUBA8,RRAS,TUBG1,TUBA4A,GFAP,BAX,TUBB2B,PRKCB
Arginine Biosynthesis IV	0.025	ASS1,OAT
Tetrahydrofolate Salvage from 5,10-methenyltetrahydrofolate	0.025	MTHFD1L,MTHFD2

Serine Biosynthesis	0.025	PSAT1,PSPH
Folate Polyglutamylation	0.025	MTHFD1L,SHMT2
Regulation of Actin-based Motility by Rho	0.026	PFN1,RHOQ,PAK3,ACTA2,ARPC5L,RHOC,ARPC3,ARHGDIA,MYL12B
Isoleucine Degradation I	0.028	HSD17B10,ACAT2,BCAT2
STAT3 Pathway	0.030	MAP3K12,NTRK2,RRAS,NTRK3,CDKN1A,CISH,SOCS2,NDUFA13
Aldosterone Signaling in Epithelial Cells	0.030	CRYAB,DNAJC19,HSPB8,DNAJC3,DNAJB9,HSPA5,HSP90B1,PLCE1,DNAJB11,HSPE1,ITPR3,DNAJB5,PRKCB
Epithelial Adherens Junction Signaling	0.032	TUBA1B,TUBB3,SNAI2,TUBA8,ACTA2,ARPC5L,RRAS,TUBG1,TUBA4A,ARPC3,PVRL1,TUBB2B
Breast Cancer Regulation by Stathmin1	0.035	TUBA1B,TUBB3,RRAS,TUBG1,TUBA4A,CDK1,TUBB2B,STMN1,CCNE1,TUBA8,CDKN1A,PPP2R2B,ITPR3,PPP2R2C,PRKCB
Proline Biosynthesis II (from Arginine)	0.035	OAT,PYCR1
Arginine Degradation VI (Arginase 2 Pathway)	0.035	OAT,PYCR1
Aspartate Degradation II	0.035	GOT1,MDH1
Germ Cell-Sertoli Cell Junction Signaling	0.037	TUBA1B,TUBB3,RRAS,RHOC,TUBG1,TUBA4A,TUBB2B,MAP3K12,RHOQ,TUBA8,PAK3,ACTA2,TGFB3
p53 Signaling	0.038	PCNA,CCND2,SNAI2,CDKN1A,GNL3,BAX,BIRC5,CHEK1,TP53I3
Antiproliferative Role of TOB in T Cell Signaling	0.039	CCNA2,CCNE1,CCNA1,TGFB3
Histidine Degradation III	0.048	MTHFD1L,MTHFD2
Gap Junction Signaling	0.049	TUBA1B,TUBB3,PLCE1,TUBA8,ACTA2,RRAS,ITPR3,TUBG1,TUBA4A,TUBB2B,HTR2A,PRKCB
