# Supplementary material for: A transcriptomal analysis of bovine oviductal epithelial cells collected during the follicular phase versus the luteal phase of the estrous cycle
Source: Reprod Biol Endocrinol. 2015 Aug 5;13:84. doi: 10.1186/s12958-015-0077-1 (PMC4524109; doi:10.1186/s12958-015-0077-1)
Supplement: Additional file 14: — Supplementary Table 10. Canonical pathways of down-regulated DEGs within epithelial cells of the isthmus in the follicular versus luteal phase groups (P < 0.05). [file 12958_2015_77_MOESM14_ESM.pdf]

Supplementary Table 10. Canonical pathways of down-regulated DEGs within epithelial cells of the isthmus in the follicular versus luteal phase groups (P < 0.05).

Canonical Pathways	P-value	Differentially expressed genes within pathway
Molecular Mechanisms of Cancer	< 0.001	FZD10,BMP4,SMAD3,BMP2,BMPR1B,MAP3K5,CCND1,BCL2,CCND3,SUFU,PRKCE,PLCB1,ARHGEF2,PRKD1,PTCH2,NAIP,LRP5,CDK7,PTCH1,PIK3C2G,GNAI1,ADCY6,RHOJ,APC,ADCY9,FOS,RASGRP1,GNAO1,IHH,FZD5,LEF1,NOTCH1,FNBP1,GNAL,WNT5A
Basal Cell Carcinoma Signaling	< 0.001	FZD10,BMP4,SUFU,BMP2,PTCH1,LEF1,FZD5,TCF7L1,APC,PTCH2,WNT5A
Role of Osteoblasts, Osteoclasts and Chondrocytes in Rheumatoid Arthritis	< 0.001	NAIP,FZD10,LRP5,BMP4,PTK2B,IL1RAPL2,BMP2,PIK3C2G,ACP5,TCF7L1,MAP3K5,GSN,CSF1R,APC,BCL2,FOS,NFAT5,CSF1,FZD5,LEF1,WNT5A
Role of Macrophages, Fibroblasts and Endothelial Cells in Rheumatoid Arthritis	< 0.001	FZD10,PDGFA,TLR8,CCND1,TRAF3IP2,NFAT5,PRKCE,PLCB1,TLR3,PRKD1,TNFSF13B,LRP5,VCAM1,IL1RAPL2,IL6R,PIK3C2G,TCF7L1,APC,FOS,CSF1,GNAO1,TLR6,LEF1,FZD5,WNT5A
Corticotropin Releasing Hormone Signaling	< 0.001	NOS1,GUCY2C,GUCY1A3,PTCH1,GNAI1,ADCY6,ADCY9,FOS,GNAO1,CAV1,PRKCE,PTCH2,PRKD1
Factors Promoting Cardiogenesis in Vertebrates	< 0.001	FZD10,LRP5,BMP4,BMP2,PRKCE,LEF1,FZD5,TCF7L1,BMPR1B,ACVR1C,APC,PRKD1
Axonal Guidance Signaling	< 0.001	FZD10,ERAP2,LRRC4C,BMP4,PDGFA,BMP2,UNC5B,ROBO1,NTN1,SEMA4C,SEMA6D,NFAT5,SUFU,ADAM28,PRKCE,PLCB1,SRGAP2,PRKD1,ACE,PTCH2,SEMA5A,PTCH1,PIK3C2G,GNAI1,EFNA4,MET,GNAO1,SEMA4G,FZD5,GNAL,WNT5A
Spermine and Spermidine Degradation I	< 0.001	SMOX,SAT1,SAT2
Role of Pattern Recognition Receptors in Recognition of Bacteria and Viruses	< 0.001	OAS2,IFNW1,TLR8,PIK3C2G,C1QA,C1QB,RNASEL,DDX58,TLR6,PRKCE,TLR3,C3AR1,PRKD1
Colorectal Cancer Metastasis Signaling	0.001	FZD10,LRP5,SMAD3,IL6R,PIK3C2G,TLR8,ADCY6,RHOJ,TCF7L1,CCND1,APC,FOS,ADCY9,TLR6,MSH6,FZD5,LEF1,TLR3,FNBP1,WNT5A
Endothelin-1 Signaling	0.001	NOS1,GUCY2C,GUCY1A3,GNAI1,ADCY6,PIK3C2G,PLD1,FOS,PLA2G6,ADCY9,GNAO1,CAV1,PLCB1,PRKCE,GNAL,PRKD1
Role of Wnt/GSK-3β Signaling in the Pathogenesis of Influenza	0.001	FZD10,IFNW1,NCOA1,LEF1,FZD5,TCF7L1,APC,WNT5A
β-alanine Degradation I	0.002	ABAT,ALDH6A1
Protein Kinase A Signaling	0.002	PTK2B,AKAP8,PTPN14,SMAD3,DUSP6,NTN1,NFAT5,DUSP10,PRKCE,PLCB1,PRKD1,PTCH2,AKAP5,PTCH1,PDE6C,GNAI1,ADCY6,TCF7L1,PTPRM,ADCY9,HIST1H1E,DUSP1,IHH,LEF1,PTPRR
CXCR4 Signaling	0.003	EGR1,GNAI1,ADCY6,PIK3C2G,RHOJ,FOS,ADCY9,GNAO1,PRKCE,PLCB1,ELMO1,PRKD1,FNBP1,GNAL
Human Embryonic Stem Cell Pluripotency	0.004	FZD10,BMP4,PDGFA,SMAD3,BMP2,PIK3C2G,LEF1,FZD5,FGFRL1,TCF7L1,APC,WNT5A
Synaptic Long Term	0.004	NOS1,PLA2G6,GUCY2C,GUCY1A3,GRID1,GNAO1,CAV1,GNAI1,P

Depression		RKCE,PLCB1,GNAL,PRKD1
Macropinocytosis Signaling	0.004	MET,CSF1,PDGFA,PIK3C2G,PRKCE,ITGB4,CSF1R,PRKD1
Wnt/β-catenin Signaling	0.005	FZD10,LRP5,APPL2,MARK2,TCF7L1,CCND1,APC,SOX6,GNAO1,LEF1,FZD5,ACVR1C,WNT5A
TGF-β Signaling	0.007	ZFYVE9,FOS,BMP4,SMAD3,BMP2,VDR,BMPR1B,ACVR1C,BCL2
Regulation of the Epithelial-Mesenchymal Transition Pathway	0.007	FZD10,EGR1,SMAD3,PIK3C2G,TCF7L1,APC,FGF13,MET,LEF1,FZD5,FGFRL1,NOTCH1,CLDN3,WNT5A
Role of NANOG in Mammalian Embryonic Stem Cell Pluripotency	0.008	LIFR,FZD10,BMP4,BMP2,PIK3C2G,FZD5,TCF7L1,BMPR1B,APC,WNT5A
GNRH Signaling	0.008	MAP3K15,ADCY9,FOS,EGR1,MAP3K13,GNAI1,ADCY6,PRKCE,PLCB1,MAP3K5,PRKD1
Sphingosine-1-phosphate Signaling	0.010	ADCY9,PTK2B,PDGFA,ACER2,GNAI1,ADCY6,PIK3C2G,PLCB1,RHOJ,FNBP1
Hepatic Fibrosis / Hepatic Stellate Cell Activation	0.010	VCAM1,IL1RAPL2,PDGFA,SMAD3,IL6R,BAMBI,TNFSF10,MYH11,COL6A6,BCL2,MET,CSF1,COL25A1,TNFSF13B
Glioblastoma Multiforme Signaling	0.011	FZD10,TSC1,PDGFA,PIK3C2G,PLCB1,RHOJ,LEF1,FZD5,CCND1,FNBP1,APC,WNT5A
Sertoli Cell-Sertoli Cell Junction Signaling	0.011	NOS1,MAP3K15,GUCY1A3,MAP3K13,MAP3K5,OCLN,CLDN4,CLDN8,CLDN16,PPAP2B,CGN,CAV1,CLDN3
IL-8 Signaling	0.012	VCAM1,PTK2B,GNAI1,PIK3C2G,RHOJ,CCND1,PLD1,BCL2,FOS,CCND3,NCF2,PRKCE,PRKD1,FNBP1
RAR Activation	0.013	SMAD3,BMP2,CDK7,ADCY6,MAP3K5,ADCY9,FOS,LRAT,DUSP1,NCOA1,PRKCE,ZBTB16,PRKD1
Tec Kinase Signaling	0.015	TEC,FOS,PTK2B,GNAO1,GNAI1,PIK3C2G,PRKCE,TNFSF10,RHOJ,FNBP1,GNAL,PRKD1
P2Y Purigenic Receptor Signaling Pathway	0.015	P2RY2,ADCY9,FOS,P2RY6,GNAI1,ADCY6,PIK3C2G,PRKCE,PLCB1,PRKD1
eNOS Signaling	0.015	ADCY9,GUCY1A3,CAV1,ADCY6,PIK3C2G,PRKCE,AQP4,ESR2,BDKRB1,NOSTRIN,PRKD1
Acetate Conversion to Acetyl-CoA	0.015	ACSS1,ACSL1
Leukocyte Extravasation Signaling	0.016	VCAM1,PTK2B,GNAI1,PIK3C2G,TEC,CLDN4,CLDN8,RASGRP1,CLDN16,NCF2,PRKCE,DLC1,CLDN3,PRKD1
Putrescine Degradation III	0.018	SMOX,SAT1,SAT2
HGF Signaling	0.019	MET,MAP3K15,FOS,MAP3K13,PIK3C2G,PRKCE,MAP3K5,CCND1,PRKD1
Ovarian Cancer Signaling	0.021	FZD10,MSH6,PIK3C2G,LEF1,FZD5,TCF7L1,CCND1,APC,WNT5A,BCL2
Salvage Pathways of Pyrimidine Deoxyribonucleotides	0.022	APOBEC3B,CDA
Citrulline-Nitric Oxide Cycle	0.022	NOS1,CAV1
Androgen Signaling	0.023	SMAD3,CDK7,GNAO1,NCOA1,GNAI1,PRKCE,CCND1,GNAL,PRKD1
Relaxin Signaling	0.025	ADCY9,FOS,GUCY2C,GUCY1A3,GNAO1,PDE6C,GNAI1,ADCY6,PI

		K3C2G,GNAL
Interferon Signaling	0.026	MX1,IRF9,IRF1,BCL2
Mouse Embryonic Stem Cell Pluripotency	0.028	LIFR,FZD10,BMP4,PIK3C2G,LEF1,FZD5,TCF7L1,APC
Thrombin Signaling	0.030	GATA5,GNAI1,ADCY6,PIK3C2G,RHOJ,ADCY9,GNAO1,PRKCE,PLCB1,ARHGEF2,FNBP1,GNAL,PRKD1
Natural Killer Cell Signaling	0.030	KLRD1,KLRB1,CD244,PIK3C2G,PRKCE,INPP5K,PRKD1,KLRC1
HER-2 Signaling in Breast Cancer	0.032	TSC1,PIK3C2G,PRKCE,ITGB4,MAP3K5,CCND1,PRKD1
Production of Nitric Oxide and Reactive Oxygen Species in Macrophages	0.036	MAP3K15,FOS,MAP3K13,NCF2,PIK3C2G,PRKCE,RHOJ,MAP3K5,FNBP1,IRF1,PRKD1,APOD
Superpathway of Melatonin Degradation	0.037	SMOX,CYP2J2,CYP2B6,UGT1A1
Melatonin Signaling	0.039	GNAO1,GNAI1,PRKCE,PLCB1,RORC,PRKD1
Cardiomyocyte Differentiation via BMP Receptors	0.041	BMP4,BMP2,BMPR1B
Thio-molybdenum Cofactor Biosynthesis	0.041	MOCOS
2-amino-3-carboxymuconate Semialdehyde Degradation to Glutaryl-CoA	0.041	ACMSD
Xanthine and Xanthosine Salvage	0.041	PNP
RANK Signaling in Osteoclasts	0.049	MAP3K15,FOS,PTK2B,MAP3K13,PIK3C2G,MAP3K5,GSN
Gap Junction Signaling	0.049	ADCY9,GUCY2C,GUCY1A3,CAV1,GNAI1,ADCY6,PIK3C2G,PRKCE,PLCB1,PRKD1
